# Synthesis and Antifungal Activity of *Chimonanthus praecox* Derivatives

**DOI:** 10.3390/molecules27175570

**Published:** 2022-08-30

**Authors:** Jinfeng Chen, Yimou Yang, Yujie Zhou, Yang Wei, Rui Zhu, Shaojun Zheng

**Affiliations:** School of Environmental and Chemical Engineering, Jiangsu University of Science and Technology, Zhenjiang 212003, China

**Keywords:** *Chimonanthus praecox* derivative, synthesis, antifungal activity, structure–activity relationship

## Abstract

To search for efficient agricultural antifungal lead compounds, 39 *Chimonanthus praecox* derivatives were designed, synthesized, and evaluated for their antifungal activities. The structures of target compounds were fully characterized by ^1^H NMR, ^13^C NMR, and MS spectra. The preliminary bioassays revealed that some compounds exhibited excellent antifungal activities in vitro. For example, the minimum inhibitory concentration (MIC) of compound **b15** against *Phytophthora infestans* was 1.95 µg mL^−1^, and the minimum inhibitory concentration (MIC) of compound **b17** against *Sclerotinia sclerotiorum* was 1.95 µg mL^−1^. Therefore, compounds **b15** and **b17** were identified as the most promising candidates for further study.

## 1. Introduction

One billion tons of crops in the world are destroyed by diseases and insect pests every year according to statistics, resulting in a 20~30% reduction in crop production [1]. The traditional chemical pesticides play a great role in food protection, but they also lead to serious ecological and environmental problems. The development of new pesticides with active natural products as lead compounds can not only find analogues with better activity but also help the products meet the needs of environmental protection and national development requirements of carbon peak and carbon neutralization [2].

**Figure 1:**
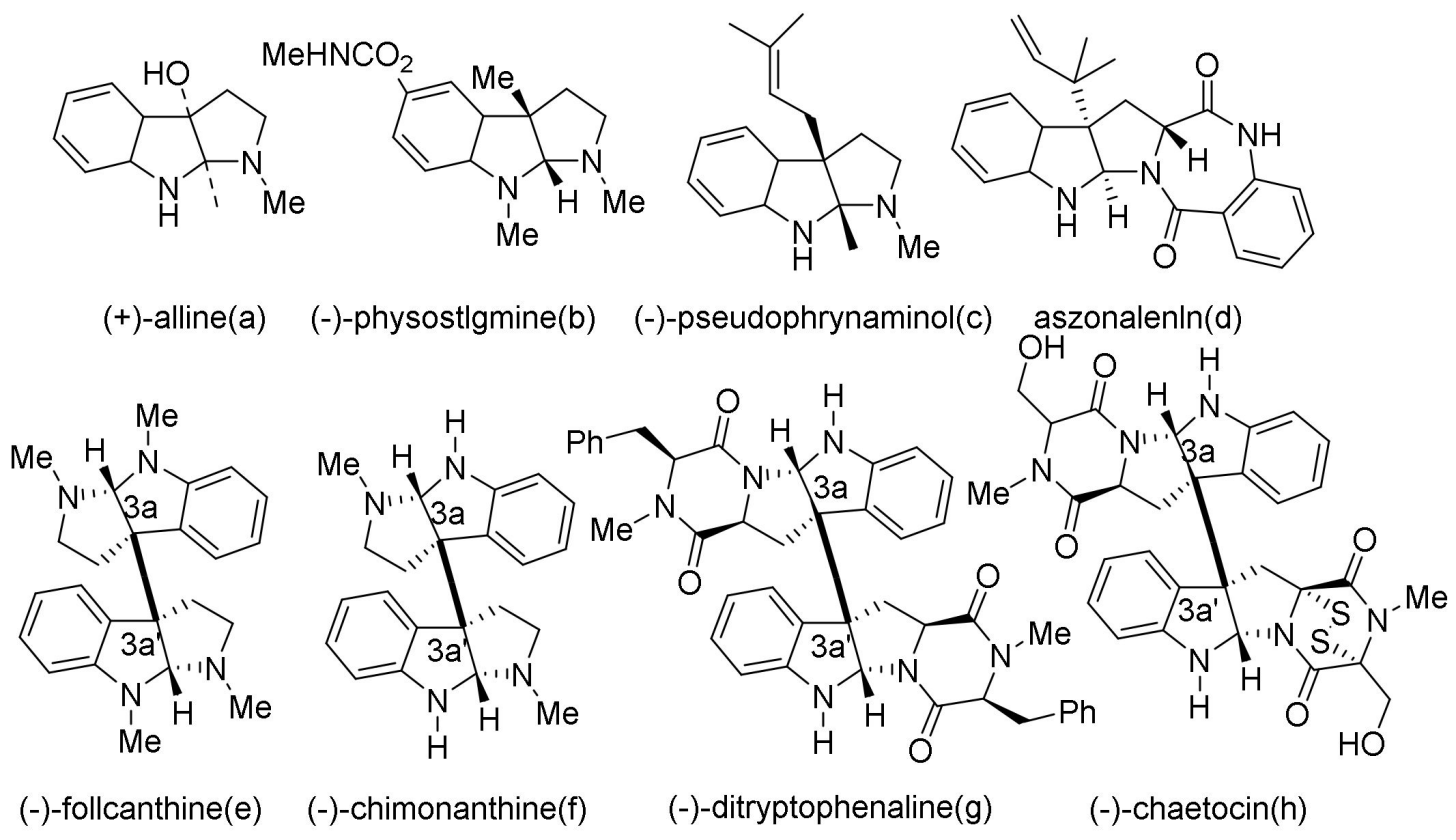
*Chimonanthus praecox* alkaloids.

*Chimonanthus praecox* alkaloids (Figure 1) are widely distributed in plants, microorganisms, and marine organisms. Their biogenic source is considered to be tryptophan through multi-step transformation [3,4]. These natural products showed a variety of biological activities [3]: *(-)-physistigmine* is a cholinesterase inhibitor that is clinically used to treat Alzheimer’s disease, myasthenia gravis, postural hypotension, delayed gastric emptying, glaucoma, and other diseases [5]; *(-)-folicanthine* has antibacterial activity [6]; *(-)-win-64745* is an excellent neurokinin antagonist [7]; *(-)-asperdimin* possess antiviral activity; and so on[8].

**Figure 2:**
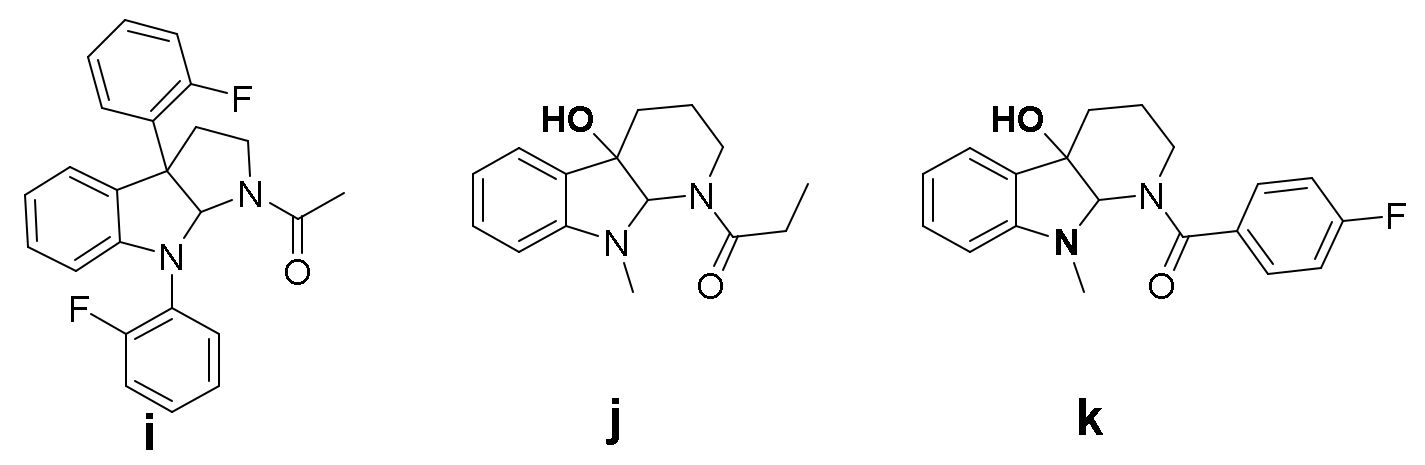
Representative *Chimonanthus praecox* compounds found by our research group.

The research on the synthesis and activity of *Chimonanthus praecox* alkaloids focuses mainly on the research and development of medicine and less on the antifungal activity of agriculture. However, pesticide research and pharmaceutical research have been learning from each other, and they have many similarities. Pesticide researchers have found that *Chimonanthus praecox* has significant inhibitory activity against *Watermelon fusariumwil*, *Fusarium oxysportium*, *Bipolaris maydis*, *Exserohilum turcium and* *Alternaria solanit* [9,10,11,12,13]. Because of their broad spectrum of biological properties, a number of studies aimed at the synthesis and antimicrobial activity of *Chimonanthus praecox* alkaloids have been reported [14,15,16,17,18]. In the past few years, our research group has been committed to the synthesis of *Chimonanthus praecox* alkaloids and research on their activity against plant pathogens. The biological testing has shown that several of the synthesized compounds have exhibited diverse and promising bioactivities (Figure 2) [19,20,21,22,23,24]; for instance, compound **i** performed better against *Verticillium dahlia* compared with chlorothalonil, with the minimum inhibitory concentration (MIC) value of 7.81 µg ml^−1^, and compounds **j** and **k** revealed potent activity against *acetylcholinesterase*, with IC_50_ values of 0.01 and 0.1 ng ml^−1^, respectively [23]. These findings inspired us to further combine the structure of *Chimonanthus praecox*-based natural product with nicotine functional group so as to acquire potential agrochemical leads for plant disease control.

## 2. Results and Discussion

### 2.1. Chemistry

**Scheme 1:**
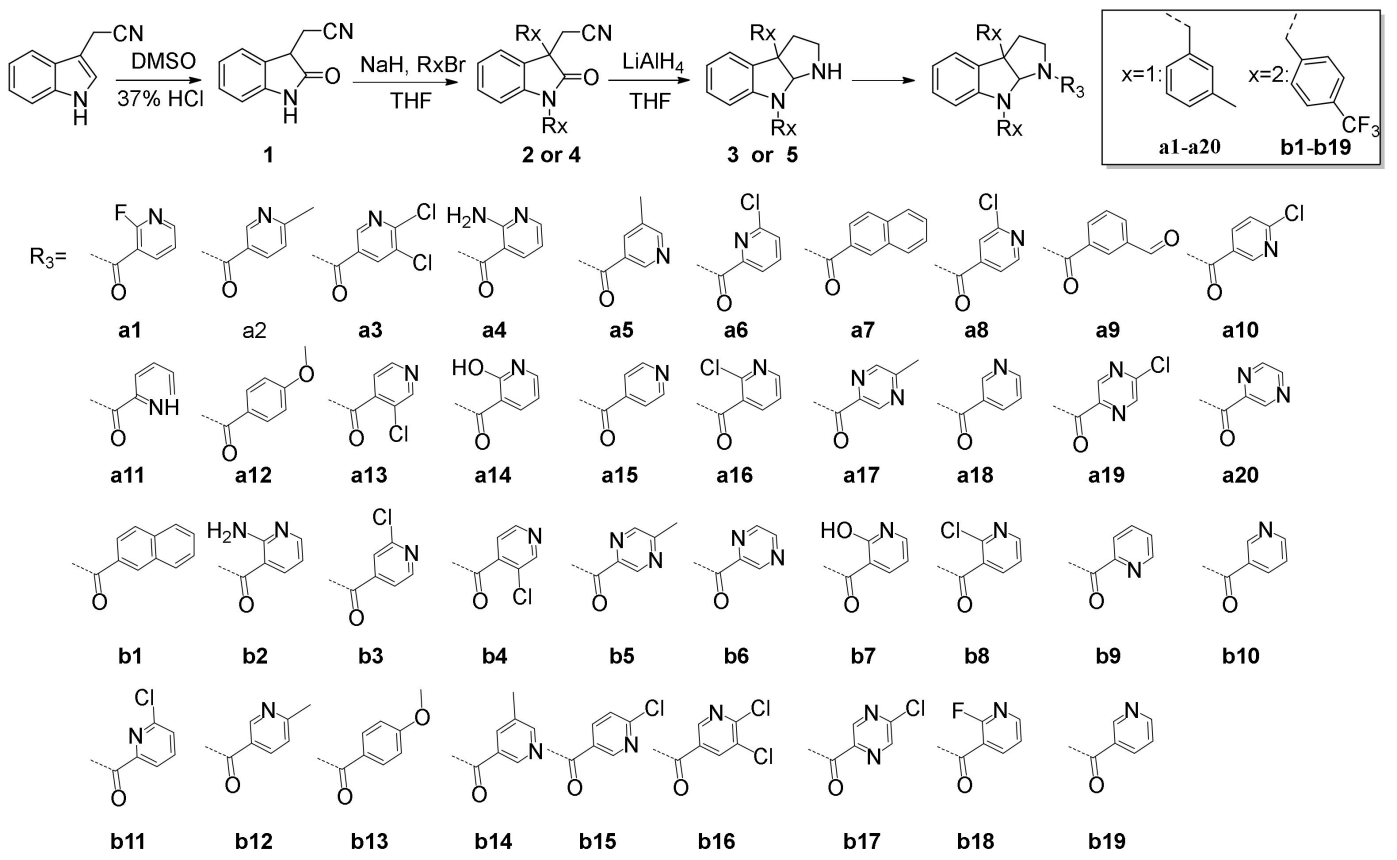
Synthesis of *Chimonanthus praecox* derivatives.

A series of *Chimonanthus praecox* analogues have been efficiently synthesized based on the methods developed in our previous work. The synthetic route to the *Chimonanthus praecox* analogues is shown in Scheme 1. Compound **1** was obtained using indole-3-acetonitrile as the starting material. Compound **1** reacted with an excess of R_1_Br or R_2_Br at the *N*-position and C-3 position using tetrahydrofuran (THF) as the solvent and sodium hydride(NaH) as a base to obtain compound **2 or 4**, respectively. Compound **3** or **5** was synthesized from **2** or **4** using lithium aluminum hydride (LiAlH_4_) as the reducing agent. Then, the expected 39 compounds were obtained based on intermediate **3** or **5.** The synthesized compounds were characterized by ^1^H NMR, ^13^C NMR, and MS. The spectra of all compounds are in the Supplementary Materials.

### 2.2. Antifungal Acitivity

The inhibitory effects of the *Chimonanthus praecox* analogues towards six plant pathogen fungi are outlined in Table 1. The MIC values were evaluated with Carbendazim and Amphotericin B as the positive controls to assay the activity of the prepared *Chimonanthus praecox* analogues against *Sclerotinia sclerotiorum*, *Altenaria solani*, *Verticillium dahliae*, *Colletotrichum orbiculare*, *Cytospora juglandis*, and *Curvularia lunata.* The preliminary bioassays showed that most of the synthesized compounds exhibited fungicidal activity. Compound **b15** exhibited significant antifungal activity against *A.* *solani*, with a MIC value of 1.95 µg mL^−1^. Compound **b17** showed the strongest antifungal activity against *S. sclerotiorum*, with a MIC value of 1.95 µg mL^−1^. Compounds **b12**, **b13**, and **b17** exhibited significant antifungal activities against *A. solani*, with the same MIC value of 3.91 µg mL^−1^. Compounds **a5** and **b10** revealed improved activity against *F. oxysporum* compared with Carbendazim and Amphotericin B, with the same MIC value of 15.16 µg mL^−1^. Compounds **b15**, **b16**, **b17**, and **b19** manifested much more activity against *C. lunata* than Carbendazim and Amphotericin B, all with the same MIC value of 15.16 µg mL^−1^. The activity of compounds **a6**, **b8**, and **b10** was more potent than Carbendazim and Amphotericin B against *A. solani,* all with the same MIC value of 15.63 µg mL^−1^. The activity of compound **b11** was more potent than Carbendazim and Amphotericin B against *V. dahliae,* with a MIC value of 15.63 µg mL^−1^. Compounds **a2**, **a3**, **a10**, **b16**, and **b17** manifested much more activity against *V. dahliae* than Carbendazim and Amphotericin B, all with the same MIC value of 15.63 µg mL^−1^.

**Figure 3:**
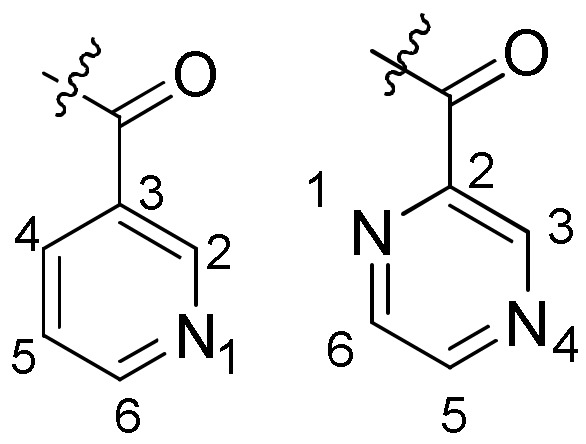
Substituent R_3_.

Although it is difficult to extract clear structure–activity relationships from the biological data, some conclusions can still be drawn (Figure 3). Firstly, the analysis of the relationship between structure and activity showed that compounds **a6**, **a10**, **b4**, **b11**, **b15**, **b16**, and **b17** contained Cl atom, and the Cl atoms that were located at C-2, C-5, or C-6 positions showed excellent antifungal activities. Compound **b16** contained two Cl atoms, and the antifungal activity was stronger than that of the target compound with one Cl. Secondly, when trifluoromethyl was introduced into the benzene ring of R_3_ group, the antifungal effect was significantly improved, and when the aromatic ring of R_1_ group was chloropyridine, the antifungal effect was significantly enhanced.

## 3. Materials and Methods

### 3.1. Instruments and Chemicals

All reagents and solvents were reagent grade or purified according to standard methods before use. Analytical thin-layer chromatography (TLC) was performed with silica gel plates using silica gel 60 GF_254_ (Qingdao Haiyang Chemical Co., Ltd., Qingdao, China). The ^1^H−NMR (400 MHz) and ^13^C−NMR (100 MHz) were obtained on an AM−500 FT−NMR spectrometer (Bruker Corporation, Fällanden, Switzerland) with CDCl_3_, acetone-*d_6_*, or DMSO-*d_6_* as the solvent and TMS as the internal standard. MS were recorded under ESI conditions using a LCQ Fleet instrument (Thermo Fisher, Waltham, MA, USA).

### 3.2. Synthesis

The general synthetic methods for the compounds **a1**–**a20** and **b1**–**b19** are depicted in Scheme 1.

#### 3.2.1. Synthesis of Compound **1**

The 3-indole acetonitrile (2.0 g, 12.8 mmol) was dissolved in dimethyl sulfoxide (DMSO) (30 mL, 281.6 mmol). Then, 100 mL 37% HCl was added. The mixture was stirred at r.t. for 2 h. The reaction mixture was evaporated under reduced pressure to remove the solvent to obtain the white solid **1** without further purification (2.1 g, 93%).

#### 3.2.2. Synthesis of Compound **2** or **4**

A round-bottom Schlenk flask was charged with NaH (0.7 g, 30 mmol), anhydrous THF (35 mL), 1-(bromomethyl)-3-(trifluoromethyl)benzene (2.1 g, 8.7 mmol) or 1-(bromomethyl)-4-methylbenzene (1.6 g, 8.7 mmol) and compound **1** (1.0 g, 5.8 mmol) respectively. The mixture was stirred at r.t. and the reaction progress was monitored by TLC until the reaction was complete (10 h~12 h). The solvent was then removed under vacuum, and the residue was extracted with ethyl acetate (3 × 30 mL) and purified by flash column chromatography (petroleum ether/EtOAc, 2:1) to produce the target product **2** (1.8 g, 63.6%) or product **4** (1.4 g, 64.5%).

#### 3.2.3. Synthesis of Compound **3** or **5**

To an oven-dried flask under nitrogen atmosphere were added LiAlH_4_ (0.6 g, 15.4 mmol), anhydrous THF (35 mL), and compound **2** (1.0 g, 2.1 mmol) or **4** (0.8 g, 2.1 mmol). The mixture was stirred at reflux for 6 h. The reaction progress was monitored by TLC until the reaction was complete (5~8 h). The solvent was then removed under vacuum, and the residue was extracted with ethyl acetate (3 × 30 mL) and purified by flash column chromatography (petroleum ether/EtOAc, 2:1) to produce the target product **3** (0.6 g, 61.4%) or 5 (0.5 g, 63.8%).

#### 3.2.4. Synthesis of *Chimonanthus praecox* Derivatives **a1**–**a20** and **b1**–**b19**

5-methylnicotinic acid (0.2 g, 1.3 mmol, 2 eq) and dichloro sulfoxide (0.3 mL, 4.3 mmol) were dissolved in 10 mL of dichloromethane (DCM). The resulting mixture was heated to reflux for 2 h. The solvent was then removed under vacuum, and the acyl chloride was obtained. A combination of 3a, 8-Bis(3-(trifluoromethyl)benzyl)-1, 2, 3, 3a, 8, 8a-hexahydrop-yrrole [2, 3-*b*]indole (0.3 g, 0.6 mmol, 1 eq) and triethylamine (0.2 mL, 1.1 mmol) was dissolved in 10 mL of dichloromethane (DCM). The prepared acyl chloride in 10 mL of DME was slowly added. The mixture was stirred at r.m. and monitored by TLC. When the reaction was complete, the solvent was removed under reduced pressure. The residue was washed with water and saturated brine. The organic layer was dried over anhydrous Na_2_SO_4_. The solvent was evaporated under reduced pressure, and the residue was purified by flash column chromatography to produce the target product **a5** (0.2 g), which was yellow and oily (Yield 64.0%). For the procedure for the remaining *Chimonanthus praecox* derivatives, refer to the steps of compound **a5**.

(3a,8-bis(4-methylbenzyl)-3,3a,8,8a-tetrahydropyrrole[2,3-*b*]indol-1(2*H*)-yl)(2-fluoropyridin-3-yl)methanone(**a1**): White solid, Melting Point: 131–133 °C, 90% Yield, ^1^H NMR (400 MHz, Acetone-*d*_6_), *δ*: 8.28 (d, *J* = 4.7 Hz, 1H), 7.85 (t, *J* = 8.3 Hz, 1H), 7.42–7.33 (m, 1H), 7.01 (dd, *J* = 15.9, 7.5 Hz, 8H), 6.87 (t, *J* = 8.0 Hz, 2H), 6.69 (dt, *J* = 14.7, 7.5 Hz, 1H), 6.15 (d, *J* = 7.9 Hz, 1H), 5.99 (s, 1H), 4.58 (d, *J* = 16.3 Hz, 1H), 4.49 (d, *J* = 16.3 Hz, 1H), 3.43–3.35 (m, 1H), 3.30–3.20 (m, 1H), 3.14 (d, *J* = 13.4 Hz, 1H), 2.96 (d, *J* = 13.4 Hz, 1H), 2.30–2.22 (m, 8H).^13^C NMR (100 MHz, Acetone-*d*_6_) δ 164.43, 160.65, 158.29, 151.44, 149.70 (CH, d, *J* = 13.0 Hz ), 149.55, 141.06, 136.98, 136.45, 136.38, 135.21, 132.56, 130.66, 129.51, 129.25, 129.00, 127.51, 123.99, 122.70, 122.65, 120.49 (d, *J* = 32.4 Hz ), 120.16, 117.97, 106.87, 83.17, 57.69, 49.98, 48.39, 44.69, 38.36, 21.00, 20.95. MS(ESI(+)) calcd for C_32_H_30_FN_3_O^+^ [M + H]^+^: 492.24; found: 492.24.

(3a,8-bis(4-methylbenzyl)-3,3a,8,8a-tetrahydropyrrolo[2,3-*b*]indol-1(2*H*)-yl)(6-methylpyridin-3-yl)methanone(**a2**): White solid, Melting Point: 153–155 °C, 89% Yield, ^1^H NMR (400 MHz, Acetone-*d*_6_), *δ*: 8.48 (s, 1H), 7.76–7.54 (m, 1H), 7.23 (d, *J* = 8.0 Hz, 1H), 6.99 (dq, *J* = 8.9, 7.4 Hz, 9H), 6.85 (d, *J* = 7.8 Hz, 2H), 6.64 (t, *J* = 7.3 Hz, 1H), 6.18–6.03 (m, 2H), 4.57 (d, *J* = 8.9 Hz, 1H), 4.43 (d, *J* = 8.9 Hz, 1H), 3.36 (d, *J* = 9.0 Hz, 1H), 3.10 (d, *J* = 13.3 Hz, 1H), 2.94 (d, *J* = 13.3 Hz, 1H), 2.48 (s, 3H), 2.28 (s, 3H), 2.24–2.20 (m, 5H).^13^C NMR (100 MHz, Acetone-*d*_6_) δ 168.43, 160.77, 151.45, 148.66, 137.13, 136.38, 136.33, 136.00, 135.28, 135.23, 132.68, 130.69, 130.06, 129.47, 129.21, 128.95, 127.43, 123.99, 122.88, 117.76, 106.74, 83.08, 57.27, 49.75, 49.68, 44.68, 38.49, 24.28, 20.98, 20.92. MS(ESI(+)) calcd for C_33_H_33_N_3_O^+^ [M + H]^+^: 488.27; found: 488.27.

(3a,8-bis(4-methylbenzyl)-3,3a,8,8a-tetrahydropyrrolo[2,3-*b*]indol-1(2*H*)-yl)(5,6-dichloropyridin-3-yl)methanone(**a3**): White solid, Melting Point: 130–132 °C, 91%, ^1^H NMR (400 MHz, Acetone-*d*_6_), *δ*: 8.28 (d, *J* = 48.1 Hz, 1H), 7.92 (s, 1H), 7.25–6.58 (m, 10H), 6.35–5.54 (m, 2H), 4.76–4.08 (m, 2H), 3.10–3.01 (m, 71.2, 43.9 Hz, 5H), 2.28 (d, *J* = 14.9 Hz, 8H).^13^C NMR (100 MHz, Acetone) δ 165.73, 151.43, 150.02, 147.00, 138.67, 137.26, 136.55, 136.45, 135.25, 133.70, 132.62, 130.74, 130.31, 130.29, 129.77, 129.55, 129.31, 129.08, 127.51, 124.06, 117.92, 106.76, 83.36, 57.39, 49.53, 44.61, 38.56, 32.15, 23.14, 20.98, 20.94. MS(ESI(+)) calcd for C_32_H_29_Cl_2_N_3_O^+^ [M + H]^+^: 542.18; found: 542.18.

(2-aminopyridin-3-yl)(3a,8-bis(4-methylbenzyl)-3,3a,8,8a-tetrahydropyrrolo[2,3-*b*]indol-1(2*H*)-yl)methanone (**a4**): White solid, Melting Point: 164–166 °C, 88% Yield, ^1^H NMR (400 MHz, Chloroform-*d*), *δ*: 7.95 (s, 1H), 7.25 (s, 1H), 6.90 (t, *J* = 9.8 Hz, 11H), 6.52 (d, *J* = 9.8 Hz, 2H), 6.10 (s, 2H), 4.34 (s, 2H), 2.97 (d, *J* = 6.5 Hz, 2H), 2.15 (d, *J* = 4.2 Hz, 10H), 1.21 (s, 1H).^13^C NMR (100 MHz, Chloroform-*d*) δ 169.00, 157.53, 150.74, 150.20, 137.13, 137.01, 136.21, 136.14, 136.04, 133.97, 131.54, 130.09, 129.03, 128.83, 128.68, 126.76, 123.49, 117.30, 113.09, 112.39, 106.28, 82.50, 56.62, 49.25, 44.43, 37.86, 21.17, 21.14. MS(ESI(+)) calcd for C_32_H_32_N_4_O^+^ [M + H]^+^: 489.26; found: 489.26.

(3a,8-bis(4-methylbenzyl)-3,3a,8,8a-tetrahydropyrrolo[2,3-*b*]indol-1(2*H*)-yl)(5-methylpyridin-3-yl)methanone (**a5**): White solid, Melting Point: 93–95 °C, 64% Yield, ^1^H NMR (400 MHz, Acetone-*d*_6_), *δ*: 8.45 (d, *J* = 6.4 Hz, 2H), 7.59 (s, 1H), 7.13–6.88 (m, 10H), 6.69 (t, *J* = 7.3 Hz, 1H), 6.20 (d, *J* = 7.8 Hz, 1H), 6.11 (s, 1H), 4.64 (d, *J* = 6.3 Hz, 1H), 4.48 (d, *J* = 6.3 Hz, 1H), 3.63–3.52 (m, 1H), 3.39 (dd, *J* = 8.4, 9.5 Hz, 1H), 3.15 (d, *J* = 13.3 Hz, 1H), 2.99 (d, *J* = 13.3 Hz, 1H), 2.37–2.25 (m, 11H).^13^C NMR (100 MHz, Acetone-*d*_6_) δ 167.70, 151.32, 150.78, 145.57, 136.50, 135.72, 135.66, 135.05, 134.61, 132.71, 132.02, 131.82, 130.02, 128.81, 128.56, 128.29, 126.77, 123.31, 117.10, 106.03, 82.39, 70.34, 56.60, 48.98, 43.93, 37.78, 31.44, 22.43, 20.28, 17.26, 13.51. MS(ESI(+)) calcd for C_33_H_33_N_3_O^+^ [M+H]^+^: 488.27; found: 488.27.

(3a,8-bis(4-methylbenzyl)-3,3a,8,8a-tetrahydropyrrolo[2,3-*b*]indol-1(2*H*)-yl)(6-chloropyridin-2-yl)methanone (**a6**): White solid, Melting Point: 130–132 °C, 83% Yield, ^1^H NMR (400 MHz, Acetone-*d*_6_), *δ*: 7.94 (t, *J* = 7.8 Hz, 1H), 7.68 (d, *J* = 7.6 Hz, 1H), 7.56–7.49 (m, 1H), 7.08–6.97 (m, 8H), 6.84 (s, 1H), 6.66 (t, *J* = 7.3 Hz, 1H), 6.14 (d, *J* = 7.8 Hz, 1H), 6.00 (s, 1H), 4.62 (d, *J* = 6.2 Hz, 1H), 4.49 (d, *J* = 6.2 Hz, 1H), 3.82–3.75 (m, 1H), 3.43 (d, *J* = 6.3 Hz, 1H), 3.09–2.88 (m, 3H), 2.26–2.12 (m, 8H).^13^C NMR (100 MHz, Acetone-*d*_6_) δ 165.65, 154.57, 150.61, 149.22, 140.30, 136.37, 135.77, 135.67, 134.55, 132.22, 130.20, 130.08, 128.84, 128.70, 128.63, 128.57, 128.26, 126.98, 125.70, 125.66, 123.24, 122.80, 117.25, 106.34, 82.75, 59.34, 56.36, 49.33, 48.68, 43.81, 37.32, 20.28. MS(ESI(+)) calcd for C_32_H_30_ClN_3_O^+^ [M + H]^+^: 508.22; found: 508.22.

(3a,8-bis(4-methylbenzyl)-3,3a,8,8a-tetrahydropyrrolo[2,3-*b*]indol-1(2*H*)-yl)(naphthalen-1-yl)methanone (**a7**): White solid, Melting Point: 147–149 °C, 95% Yield, ^1^H NMR (400 MHz, Acetone-*d*_6_), *δ*: 7.93 (d, *J* = 8.3 Hz, 2H), 7.63–7.34 (m, 4H), 7.26 (d, *J* = 6.4 Hz, 1H), 7.10 (s, 7H), 7.00 (dd, *J* = 10.9, 7.9 Hz, 3H), 6.66 (t, *J* = 7.4 Hz, 1H), 6.30–6.20 (m, 2H), 4.73 (d, *J* = 6.3 Hz, 2H), 3.18–2.97 (m, 4H), 2.34 (d, *J* = 7.1 Hz, 6H), 2.18 (d, *J* = 6.9 Hz, 2H).^13^C NMR (100 MHz, Acetone-*d*_6_) δ 169.57, 151.58, 151.55, 137.35, 136.56, 136.46, 136.05, 135.30, 134.16, 133.14, 131.02, 130.95, 130.01, 129.65, 129.61, 129.39, 129.03, 129.01, 127.65, 127.54, 127.01, 125.87, 125.51, 124.54, 123.99, 117.88, 106.61, 100.70, 57.70, 50.04, 48.49, 44.51, 38.38, 21.03, 20.97. MS(ESI(+)) calcd for C_37_H_34_N_2_O^+^ [M + H]^+^: 523.27; found: 523.27.

(3a,8-bis(4-methylbenzyl)-3,3a,8,8a-tetrahydropyrrolo[2,3-*b*]indol-1(2*H*)-yl)(2-chloropyridin-4-yl)methanone (**a8**): Light yellow solid, Melting Point: 107–109 °C, 90% Yield, ^1^H NMR (400 MHz, Acetone-*d*_6_), *δ*: 8.41 (dd, *J* = 4.9, 0.6 Hz, 1H), 7.30–7.25 (m, 2H), 7.01–6.92 (m, 9H), 6.86 (d, *J* = 8.0 Hz, 1H), 6.65 (td, *J* = 7.4, 0.8 Hz, 1H), 6.19 (d, *J* = 7.8 Hz, 1H), 6.00 (s, 1H), 4.51 (d, *J* = 6.4 Hz, 2H), 3.43 (d, *J* = 1.9 Hz, 1H), 3.29–3.15 (m, 1H), 3.11 (d, *J* = 13.4 Hz, 1H), 2.95 (d, *J* = 13.4 Hz, 1H), 2.29 (s, 2H), 2.28–2.18 (m, 6H).^13^C NMR (100 MHz, Acetone-*d*_6_) δ 166.69, 151.87, 151.34, 150.86, 148.04, 137.20, 136.52, 136.41, 135.19, 132.60, 130.68, 129.51, 129.27, 129.03, 127.46, 123.99, 122.64, 121.23, 117.91, 106.73, 83.40, 57.39, 49.71, 49.27, 44.53, 38.52, 20.99, 20.95. MS(ESI(+)) calcd for C_32_H_30_ClN_3_O^+^ [M + H]^+^: 508.22; found: 508.22.

2-(3a,8-bis(4-methylbenzyl)-1,2,3,3a,8,8a-hexahydropyrrolo[2,3-*b*]indole-1-carbonyl)benzaldehyde (**a9**): White solid, Melting Point: 97–99 °C, 92% Yield, ^1^H NMR (400 MHz, Acetone-*d*_6_), *δ*: 7.58–7.49 (m, 4H), 7.08–6.97 (m, 7H), 6.73–6.54 (m, 4H), 6.21 (d, *J* = 7.8 Hz, 1H), 5.99 (s, 1H), 5.39 (s, 1H), 4.39 (d, *J* = 6.6 Hz, 3H), 3.57 (d, *J* = 9.5 Hz, 1H), 3.08–2.81 (m, 2H), 2.50–2.17 (m, 8H).^13^C NMR (100 MHz, Acetone-*d*_6_) δ 169.56, 151.55, 137.35, 136.56, 136.46, 136.05, 135.30, 134.16, 133.14, 130.95, 130.01, 129.65, 129.61, 129.39, 129.03, 129.01, 127.65, 127.54, 127.01, 125.87, 125.51, 124.54, 123.99, 117.88, 106.61, 57.70, 50.04, 48.49, 44.51, 38.38, 21.03, 20.97. MS(ESI(+)) calcd for C_34_H_32_N_2_O_2_ ^+^ [M + H]^+^: 501.25; found: 501.25.

(3a,8-bis(4-methylbenzyl)-3,3a,8,8a-tetrahydropyrrolo[2,3-*b*]indol-1(2*H*)-yl)(6-chloropyridin-3-yl)methanone (**a1**0): White solid, Melting Point: 117–119 °C, 94% Yield, ^1^H NMR (400 MHz, Acetone-*d*_6_), *δ*: 8.45 (d, *J* = 1.5 Hz, 1H), 7.88 (dd, *J* = 8.2, 1.9 Hz, 1H), 7.51 (d, *J* = 8.2 Hz, 1H), 7.09–6.87 (m, 10H), 6.69 (t, *J* = 7.3 Hz, 1H), 6.20 (d, *J* = 7.8 Hz, 1H), 6.09 (s, 1H), 4.62 (d, *J* = 16.3 Hz, 1H), 4.48 (d, *J* = 16.3 Hz, 1H), 3.60 (dd, *J* = 7.8, 4.5 Hz, 1H), 3.42 (dd, *J* = 18.4, 9.5 Hz, 1H), 3.15 (d, *J* = 13.2 Hz, 1H), 2.98 (d, *J* = 13.3 Hz, 1H), 2.30 (t, *J* = 11.0 Hz, 8H).^13^C NMR (100 MHz, Acetone-*d*_6_) δ 167.25, 152.95, 151.57, 149.61, 139.29, 137.27, 136.61, 136.52, 135.38, 132.77, 132.38, 130.85, 129.66, 129.40, 129.16, 127.59, 124.63, 124.17, 118.01, 106.92, 83.34, 71.19, 57.47, 49.81, 44.75, 38.56, 21.12, 21.05. MS(ESI(+)) calcd for C_32_H_30_ClN_3_O^+^ [M + H]^+^: 508.22; found: 508.22.

(3a,8-bis(4-methylbenzyl)-3,3a,8,8a-tetrahydropyrrolo[2,3-*b*]indol-1(2*H*)-yl)(pyridin-2-yl)methanone (**a11**): Light yellow solid, Melting Point: 120–122 °C, 90% Yield, ^1^H NMR (400 MHz, Acetone-*d*_6_), *δ*: 8.55–8.45 (m, 1H), 7.92–7.86 (m, 1H), 7.73–7.68 (m, 1H), 7.49–7.41 (m, 1H), 7.06–6.96 (m, 7H), 6.87 (t, *J* = 8.5 Hz, 3H), 6.65 (dd, *J* = 7.4, 6.7 Hz, 1H), 6.16 (d, *J* = 7.8 Hz, 1H), 6.04 (s, 1H), 4.65 (d, *J* = 16.2 Hz, 1H), 4.50 (d, *J* = 16.2 Hz, 1H), 3.83 (ddd, *J* = 11.5, 6.1, 2.1 Hz, 1H), 3.42 (td, *J* = 11.3, 6.8 Hz, 1H), 3.09 (dd, *J* = 10.9, 5.7 Hz, 1H), 2.94 (dd, *J* = 13.5, 2.3 Hz, 1H), 2.30 (s, 3H), 2.26 (s, 3H), 2.22 (d, *J* = 3.0 Hz, 2H).^13^C NMR (100 MHz, Acetone-*d*_6_) δ 167.91, 155.13, 151.42, 148.67, 137.50, 137.15, 136.40, 136.30, 135.31, 133.00, 130.88, 130.75, 129.49, 129.32, 129.23, 128.87, 127.67, 126.76, 125.59, 125.32, 124.51, 123.89, 117.80, 106.92, 83.40, 57.04, 50.04, 49.30, 44.63, 38.26, 20.99, 20.94. MS(ESI(+)) calcd for C_32_H_31_N_3_O^+^ [M + H]^+^: 474.25; found: 474.25.

(3a,8-bis(4-methylbenzyl)-3,3a,8,8a-tetrahydropyrrolo[2,3-*b*]indol-1(2*H*)-yl)(4-methoxyphenyl)methanone (**a12**): Light yellow solid, Melting Point: 119–121 °C, 91% Yield, ^1^H NMR (400 MHz, Acetone-*d*_6_), *δ*: 7.42 (s, 2H), 7.08–6.84 (m, 13H), 6.64 (t, *J* = 7.3 Hz, 1H), 6.14 (d, *J* = 7.7 Hz, 1H), 4.50 (dd, *J* = 80.9, 15.2 Hz, 2H), 3.82 (s, 3H), 3.69–3.23 (m, 2H), 3.10 (d, *J* = 13.3 Hz, 1H), 2.94 (d, *J* = 13.3 Hz, 1H), 2.28 (d, *J* = 16.9 Hz, 6H), 2.22–2.15 (m, 2H).^13^C NMR (100 MHz, Acetone-*d*_6_) δ 170.04, 161.91, 151.57, 137.14, 136.35, 136.29, 135.37, 132.90, 132.83, 132.80, 132.70, 130.74, 130.30, 129.46, 129.32, 129.22, 128.90, 127.48, 127.16, 123.98, 117.69, 113.90, 106.73, 83.02, 57.18, 55.54, 50.07, 49.59, 44.76, 38.51, 21.00, 20.94. MS(ESI(+)) calcd for C_34_H_34_N_2_O_2_^+^ [M + H]^+^: 503.27; found: 503.27.

(3a,8-bis(4-methylbenzyl)-3,3a,8,8a-tetrahydropyrrolo[2,3-*b*]indol-1(2*H*)-yl)(3-chloropyridin-4-yl)methanone (**a13**): White solid, Melting Point: 119–121 °C, 93% Yield, ^1^H NMR (400 MHz, Acetone-*d*_6_), *δ*: 8.61 (s, 1H), 8.54 (d, *J* = 4.7 Hz, 1H), 7.11–6.88 (m, 11H), 6.69–6.62 (m, 1H), 6.18 (d, *J* = 7.6 Hz, 1H), 5.98 (s, 1H), 4.64 (d, *J* = 16.3 Hz, 1H), 4.51 (d, *J* = 16.3 Hz, 1H), 3.25–3.07 (m, 3H), 2.98 (d, *J* = 13.4 Hz, 1H), 2.29 (d, *J* = 9.1 Hz, 7H), 2.22–2.17 (m, 1H).^13^C NMR (100 MHz, Acetone-*d*_6_) δ 165.05, 151.39, 150.21, 149.17, 144.35, 136.99, 136.53, 136.45, 135.14, 132.65, 130.76, 129.60, 129.54, 129.32, 129.22, 129.04, 127.83, 127.60, 123.99, 122.49, 118.04, 106.78, 82.92, 57.87, 49.92, 47.87, 44.48, 38.58, 20.98. MS(ESI(+)) calcd for C_32_H_30_ClN_3_O^+^ [M + H]^+^: 508.22; found: 508.22.

(3a,8-bis(4-methylbenzyl)-3,3a,8,8a-tetrahydropyrrolo[2,3-*b*]indol-1(2*H*)-yl)(2-hydroxypyridin-3-yl)methanone (**a14**): Yellow oily, 83% Yield, ^1^H NMR (400 MHz, DMSO-*d*_6_), *δ*: 11.92 (d, *J* = 4.6 Hz, 1H), 7.46–7.35 (m, 2H), 7.06 (d, *J* = 7.1 Hz, 1H), 6.99–6.96 (m, 3H), 6.93–6.76 (m, 6H), 6.61 (d, *J* = 7.2 Hz, 1H), 6.26–6.16 (m, 1H), 6.02 (d, *J* = 7.8 Hz, 1H), 5.80 (s, 1H), 4.42 (d, *J* = 8.4 Hz, 2H), 3.07 (d, *J* = 13.4 Hz, 2H), 2.86 (d, *J* = 13.3 Hz, 1H), 2.25–2.20 (m, 7H), 2.10 (dd, *J* = 6.7, 2.5 Hz, 2H).^13^C NMR (100 MHz, DMSO-*d*_6_).δ 166.17, 158.73, 150.08, 140.48, 137.30, 135.77, 135.05, 134.26, 131.62, 131.21, 129.63, 129.55, 128.54, 128.36, 128.29, 128.16, 127.94, 126.50, 125.81, 123.58, 123.06, 116.61, 105.43, 104.50, 82.35, 81.42, 58.18, 56.33, 48.49, 46.81, 43.44, 20.57. MS(ESI(+)) calcd for C_32_H_31_N_3_O_2_^+^ [M + H]^+^: 490.25; found: 490.25.

(3a,8-bis(4-methylbenzyl)-3,3a,8,8a-tetrahydropyrrolo[2,3-*b*]indol-1(2*H*)-yl)(pyridin-4-yl)methanone (**a15**): Yellow oily, 92% Yield, ^1^H NMR (400 MHz, Acetone-*d*_6_), *δ*: 8.62 (dd, *J* = 4.4, 1.5 Hz, 2H), 7.30 (dd, *J* = 4.4, 1.6 Hz, 2H), 7.09–6.96 (m, 9H), 6.87 (d, *J* = 8.0 Hz,2H), 6.66 (t, *J* = 7.3 Hz, 1H), 6.18 (d, *J* = 7.8 Hz, 1H), 6.03 (s, 1H), 4.60 (d, *J* = 16.3 Hz, 1H), 4.47 (d, *J* = 16.3 Hz, 1H), 3.35–3.27 (m, 1H), 3.12 (d, *J* = 13.3 Hz, 1H), 2.96 (d, *J* = 13.4 Hz, 1H), 2.30 (s, 3H), 2.26 (s, 3H), 2.24–2.19 (m, 2H).^13^C NMR (100 MHz, Acetone-*d*_6_) δ 168.03, 151.19, 150.47, 144.29, 136.92, 136.25, 136.16, 135.01, 132.44, 130.77, 130.48, 129.31, 129.04, 128.79, 127.27, 126.78, 123.79, 122.25, 121.86, 117.67, 106.60, 83.01, 57.18, 49.58, 49.22, 44.41, 38.20, 20.80, 20.73. MS(ESI(+)) calcd for C_32_H_31_N_3_O ^+^ [M + H]^+^: 474.25; found: 474.25.

(3a,8-bis(4-methylbenzyl)-3,3a,8,8a-tetrahydropyrrolo[2,3-*b*]indol-1(2*H*)-yl)(2-chloropyridin-3-yl)methanone (**a16**): Light yellow solid, Melting Point: 118–120 °C, 90% Yield, ^1^H NMR (400 MHz, Acetone-*d*_6_), *δ*: 8.42 (dd, *J* = 4.7, 1.8 Hz, 1H), 7.46–7.39 (m, 1H), 7.31–6.92 (m, 10H), 6.90 (d, *J* = 8.0 Hz, 2H), 6.66 (t, *J* = 7.4 Hz, 1H), 6.18 (d, *J* = 6.4 Hz, 1H), 6.01 (s, 1H), 4.68 (d, *J* = 16.3 Hz, 1H), 4.53 (d, *J* = 16.3 Hz, 1H), 3.25 (dd, *J* = 9.8, 7.6 Hz, 1H), 3.16 (d, *J* = 13.3 Hz, 1H), 2.98 (d, *J* = 13.4 Hz, 1H), 2.31 (s, 3H), 2.29 (s, 3H), 2.26–2.15 (m, 2H).^13^C NMR (100 MHz, Acetone-*d*_6_)δ 165.56, 151.20, 150.58, 150.52, 146.77, 137.48, 136.81, 136.23, 136.16, 134.93, 133.57, 132.42, 130.52, 129.29, 129.07, 128.78, 127.35, 123.74), 123.53, 117.73, 106.48, 82.66, 57.63, 49.62, 47.77, 44.30, 38.36, 20.80, 20.76. MS(ESI(+)) calcd for C_32_H_30_ClN_3_O^+^ [M + H]^+^: 508.22; found: 508.22.

(3a,8-bis(4-methylbenzyl)-3,3a,8,8a-tetrahydropyrrolo[2,3-*b*]indol-1(2*H*)-yl)(5-methylpyrazin-2-yl)methanone (**a17**): Light yellow solid, Melting Point: 117–119 °C, 89% Yield, ^1^H NMR (400 MHz, Acetone-*d*_6_), *δ*: 8.75 (d, *J* = 1.3 Hz, 1H), 8.45 (d, *J* = 0.8 Hz, 1H), 7.08–6.93 (m, 10H), 6.67–6.63 (m, 1H), 6.17 (d, *J* = 7.8 Hz, 1H), 4.62 (d, *J* = 16.1 Hz, 1H), 4.50 (d, *J* = 16.1 Hz, 1H), 3.47–3.37 (m, 1H), 3.08 (d, *J* = 13.4 Hz, 1H), 2.55 (s, 2H), 2.46 (s, 1H), 2.30–2.21 (m, 11H).^13^C NMR (100 MHz, Acetone-*d*_6_ ) δ 166.31, 155.96, 151.31, 147.20, 145.06, 142.71, 137.07, 136.45, 136.34, 135.22, 132.94, 130.89, 130.75, 129.49, 129.36, 129.24, 129.02, 128.92, 127.65, 126.34, 123.90, 117.91, 107.00, 83.48, 57.00, 50.10, 49.23, 44.53, 38.12, 21.48, 20.98, 20.94. MS(ESI(+)) calcd for C_32_H_32_N_4_O^+^ [M + H]^+^: 489.26; found: 489.26.

(3a,8-bis(4-methylbenzyl)-3,3a,8,8a-tetrahydropyrrolo[2,3-*b*]indol-1(2*H*)-yl)(pyridin-3-yl)methanone (**a18**): Light yellow solid, Melting Point: 92–94 °C, 93% Yield, ^1^H NMR (400 MHz, Acetone-*d*_6_), *δ*: 8.62–8.59 (m, 2H), 7.77 (d, *J* = 7.8 Hz, 1H), 7.37 (dd, *J* = 7.7, 4.9 Hz, 1H), 7.24–6.90 (m, 10H), 6.87 (d, *J* = 7.8 Hz, 2H), 6.66 (t, *J* = 7.4 Hz, 1H), 6.18 (d, *J* = 7.8 Hz, 1H), 6.09 (s, 1H), 4.61 (d, *J* = 16.3 Hz, 1H), 4.47 (d, *J* = 16.3 Hz, 1H), 3.12 (d, *J* = 13.3 Hz, 1H), 2.98–2.89 (m, 1H), 2.30 (s, 3H), 2.26 (s, 3H), 2.24–2.20 (m, 2H).^13^C NMR (100 MHz, Acetone-*d*_6_)δ 168.17, 151.57, 151.40, 149.11, 137.10, 136.37, 136.30, 135.50, 135.22, 132.91, 132.62, 130.66, 129.47, 129.21, 128.96, 127.43, 123.98, 123.65, 117.78, 106.74, 83.15, 57.27, 49.68, 44.64, 38.47, 20.99, 20.93. MS(ESI(+)) calcd for C_32_H_31_N_3_O^+^ [M + H]^+^: 474.25; found: 474.25.

(3a,8-bis(4-methylbenzyl)-3,3a,8,8a-tetrahydropyrrolo[2,3-*b*]indol-1(2*H*)-yl)(5-chloropyrazin-2-yl)methanone (**a19**): Light yellow solid, Melting Point: 131–133 °C, 84% Yield, ^1^H NMR (400 MHz, Acetone-*d*_6_), *δ*: 8.69 (d, *J* = 1.3 Hz, 1H), 8.66 (d, *J* = 1.3 Hz, 1H), 7.08 (dd, *J* = 7.3, 0.8 Hz, 1H), 7.01 (dt, *J* = 7.9, 4.6 Hz, 8H), 6.95 (dd, *J* = 10.0, 4.6 Hz, 1H), 6.85 (d, *J* = 7.9 Hz, 2H), 6.31–6.18 (m, 2H), 6.01 (s, 1H), 4.61 (d, *J* = 16.1 Hz, 1H), 4.50 (d, *J* = 16.2 Hz, 1H), 3.88–3.76 (m, 1H), 3.46–3.38 (m, 1H), 3.07–3.00 (m, 1H), 2.94 (d, *J* = 13.4 Hz, 1H), 2.29 (s, 3H), 2.26 (s, 3H).^13^C NMR (100 MHz, Acetone-*d*_6_) δ 165.16, 151.24, 150.35, 148.45, 145.69, 143.05, 137.00, 136.53, 136.39, 135.15, 132.88, 130.76, 129.52, 129.27, 128.98, 127.65, 126.16, 123.92, 118.04, 107.07, 83.52, 57.10, 50.12, 49.23, 44.45, 37.97, 20.97, 20.93. MS(ESI(+)) calcd for C_31_H_29_ClN_4_O^+^ [M + H]^+^: 509.21; found: 509.21.

(3a,8-bis(4-methylbenzyl)-3,3a,8,8a-tetrahydropyrrolo[2,3-*b*]indol-1(2*H*)-yl)(pyrazin-2-yl)methanone (**a20**): White solid, Melting Point: 105–107 °C, 91% Yield, ^1^H NMR (400 MHz, Acetone-*d*_6_), *δ*: 8.86 (s, 1H), 8.67 (d, *J* = 2.5 Hz, 1H), 8.59–8.53 (m, 1H), 7.05–6.84 (m, 10H), 6.68–6.62 (m, 1H), 6.17 (t, *J* = 7.5 Hz, 1H), 6.03 (s, 1H), 4.56 (d, *J* = 16.2 Hz, 2H), 3.42 (td, *J* = 11.0, 7.7 Hz, 1H), 2.96–2.87 (m, 3H), 2.29 (s, 2H), 2.25 (t, *J* = 5.5 Hz, 6H).^13^C NMR (100 MHz, Acetone-*d*_6_) δ 165.97, 151.15, 150.03, 146.58, 146.30, 145.92, 143.18, 142.39, 136.90, 136.33, 136.22, 135.05, 132.77, 130.68, 130.60, 129.35, 129.10, 128.79, 127.50, 126.25, 123.76), 117.81, 106.87, 83.36, 56.88, 49.94, 49.01, 44.33, 37.93, 20.78, 20.74. MS(ESI(+)) calcd for C_31_H_30_N_4_O^+^ [M + H]^+^: 475.25; found: 475.25.

(3a,8-bis(3-(trifluoromethyl)benzyl)-3,3a,8,8a-tetrahydropyrrolo[2,3-*b*]indol-1(2*H*)-yl)(naphthalen-1-yl)methanone (**b1**): White solid, Melting Point: 142–144 °C, 94% Yield, ^1^H NMR (400 MHz, DMSO- *d*_6_), *δ*: 7.97 (d, *J* = 8.2 Hz, 2H), 7.64–7.35 (m, 10H), 7.32–7.10 (m, 4H), 7.01 (t, *J* = 7.7 Hz, 1H), 6.70 (t, *J* = 7.3 Hz, 1H), 6.21–6.04 (m, 2H), 4.73 (d, *J* = 16.9 Hz, 1H), 4.56 (d, *J* = 16.9 Hz, 1H), 3.43–3.28 (m, 2H), 3.14–2.88 (m, 2H), 2.10 (d, *J* = 16.3 Hz, 2H).^13^C NMR (100 MHz, DMSO- *d*_6_) δ 169.21, 150.84, 141.38, 139.45, 134.87, 134.44, 133.45, 131.59 (q, *J* = 35.3 Hz), 130.88, 130.03, 129.85, 129.73, 129.66, 129.41, 129.28, 129.15, 128.92, 128.61, 127.51, 126.95, 126.76, 126.14, 126.08, 125.71, 124.82 (q, *J* = 268.2 Hz), 124.24, 124.16, 123.90, 123.56, 118.11, 106.06, 82.29, 57.28, 49.56, 48.31, 43.77, 38.22. MS(ESI(+)) calcd for C_37_H_28_F_6_N_2_O^+^ [M + H]^+^: 631.22; found: 631.22.

(3a,8-bis(3-(trifluoromethyl)benzyl)-3,3a,8,8a-tetrahydropyrrolo[2,3-*b*]indol-1(2*H*)-yl)(naphthalen-1-yl)methanone (**b2**): White solid, Melting Point: 161–163 °C, 92% Yield, ^1^H NMR (400 MHz, DMSO- *d*_6_), *δ*: 7.98 (dd, *J* = 3.2, 1.3 Hz, 1H), 7.50 (t, *J* = 8.2 Hz, 2H), 7.37–7.30 (m, 3H), 7.25 (t, *J* = 7.7 Hz, 2H), 7.18 (d, *J* = 7.2 Hz, 1H), 7.08 (s, 1H), 7.00–6.90 (m, 2H), 6.69 (td, *J* = 7.4, 0.8 Hz, 1H), 6.49 (d, *J* = 0.5 Hz, 1H), 6.18 (s, 2H), 6.04–5.91 (m, 1H), 4.54–4.39 (m, 1H), 4.38–4.26 (m, 1H), 3.60–3.42 (m, 1H), 3.32 (d, *J* = 8.0 Hz, 2H), 3.13 (d, *J* = 13.1 Hz, 2H), 2.27–2.13 (m, 2H).^13^C NMR (100 MHz, DMSO- *d*_6_) δ 168.88, 157.22, 150.89, 150.27, 139.55, 136.94, 134.14, 131.15 (q, *J* = 35.3 Hz), 130.61, 129.69, 129.58, 129.38, 129.27, 129.16, 129.09, 128.96, 128.76, 126.47, 126.07, 126.00, 124.22 (q, *J* = 268.2 Hz), 123.79, 123.61, 123.38, 117.96, 111.88, 105.98, 82.23, 57.04, 49.00, 44.05, 38.71. MS(ESI(+)) calcd for C_32_H_26_F_6_N_4_O^+^ [M + H]^+^: 597.21; found: 597.21.

(3a,8-bis(3-(trifluoromethyl)benzyl)-3,3a,8,8a-tetrahydropyrrolo[2,3-*b*]indol-1(2*H*)-yl)(2-chloropyridin-4-yl)methanone (**b3**): Yellow oily, 89% Yield, ^1^H NMR (400 MHz, Acetone-*d*_6_), *δ*: 8.53 (d, *J* = 5.0 Hz, 1H), 7.62 (d, *J* = 7.7 Hz, 3H), 7.49 (dd, *J* = 9.5, 7.3 Hz, 3H), 7.39 (d, *J* = 7.7 Hz, 1H), 7.26 (dd, *J* = 7.8, 7.1 Hz, 2H), 7.12 (td, *J* = 7.7, 1.2 Hz, 1H), 6.84 (d, *J* = 3.9 Hz, 1H), 6.24 (d, *J* = 7.9 Hz, 1H), 6.06 (s, 1H), 4.68 (d, *J* = 16.8 Hz, 1H), 4.57 (d, *J* = 16.8 Hz, 1H), 3.71 (ddd, *J* = 10.9, 5.6, 2.8 Hz, 1H), 3.55–3.42 (m, 2H), 3.28 (d, *J* = 13.2 Hz, 1H), 2.48–2.45 (m, 1H), 2.20–2.11 (m, 1H), 1.46–1.26 (m, 2H).^13^C NMR (100 MHz, Acetone-*d*_6_) δ 166.93, 151.96, 151.39, 150.93, 147.75, 141.76, 139.70, 134.35, 131.47 (q, *J* = 35.3 Hz), 131.05, 130.73, 130.41, 130.32, 129.84, 129.49, 129.34, 127.08, 127.04, 126.51, 126.41, 124.37 (q, *J* = 268.2 Hz), 123.96, 122.68, 121.27, 118.61, 106.79, 83.58, 57.61, 49.94, 49.43, 44.80, 38.59. MS(ESI(+)) calcd for C_32_H_24_ClF_6_N_3_O^+^ [M + H]^+^: 616.16; found: 616.16.

(3a,8-bis(3-(trifluoromethyl)benzyl)-3,3a,8,8a-tetrahydropyrrolo[2,3-*b*]indol-1(2*H*)-yl)(3-chloropyridin-4-yl)methanone (**b4**): Yellow oily, 85% Yield, ^1^H NMR (400 MHz, Acetone-*d*_6_), *δ*: 8.64 (s, 1H), 8.56 (d, *J* = 4.8 Hz, 1H), 7.63–7.47 (m, 4H), 7.46–7.34 (m, 3H), 7.24–7.11 (m, 4H), 7.03 (td, *J* = 7.7, 1.2 Hz, 1H), 6.74 (d, *J* = 3.9 Hz, 1H), 6.17 (d, *J* = 7.9 Hz, 1H), 5.96 (s, 1H), 4.63 (s, 1H), 4.57 (d, *J* = 16.7 Hz, 1H), 3.40 (d, *J* = 13.3 Hz, 1H), 3.35–3.29 (m, 1H), 3.26–3.21 (m, 1H), 2.39–2.34 (m, 2H).^13^C NMR (100 MHz, Acetone-*d*_6_)δ 165.29, 151.40, 150.26, 149.20, 144.08, 141.62, 139.63, 134.47, 131.61 (q, *J* = 35.3 Hz), 131.18, 129.89, 129.50, 129.44, 127.79, 127.19, 127.15, 124.34 (q, *J* = 268.2 Hz), 124.16, 124.11, 124.07, 124.02, 122.45, 118.76, 118.21, 106.85, 83.09, 58.02, 50.13, 48.01, 44.59, 38.62. MS(ESI(+)) calcd for C_32_H_24_ClF_6_N_3_O^+^ [M + H]^+^: 616.16; found: 616.16.

(3a,8-bis(3-(trifluoromethyl)benzyl)-3,3a,8,8a-tetrahydropyrrolo[2,3-*b*]indol-1(2*H*)-yl)(5-methylpyrazin-2-yl)methanone (**b5**): Yellow oily, 88% Yield, ^1^H NMR (400 MHz, Acetone-*d*_6_), *δ*: 8.74 (d, *J* = 1.4 Hz, 1H), 8.50–8.47 (m, 1H), 7.55 (d, *J* = 8.4 Hz, 2H), 7.51–7.27 (m, 6H), 7.18–7.10 (m, 2H), 7.02 (dd, *J* = 5.7, 1.7 Hz, 1H), 6.77–6.72 (m, 1H), 6.11 (d, *J* = 7.9 Hz, 1H), 6.03 (s, 1H), 4.63 (d, *J* = 16.7 Hz, 1H), 4.47 (d, *J* = 16.7 Hz, 1H), 3.54–3.45 (m, 1H), 3.37 (d, *J* = 13.2 Hz, 1H), 3.20 (d, *J* = 13.2 Hz, 1H), 2.56 (s, 3H), 2.40–2.29 (m, 2H).^13^C NMR (100 MHz, Acetone-*d*_6_) δ 166.39, 156.15, 151.29, 146.90, 146.09, 145.02, 142.77, 141.71, 139.81, 134.41, 131.81 (q, *J* = 35.3 Hz), 131.10, 129.77, 129.38, 129.33, 127.13, 127.10, 124.30 (q, *J* = 268.2 Hz), 124.07, 124.03, 123.99, 123.92, 118.55, 106.84, 83.94, 57.12, 50.28, 49.34, 44.77, 38.54, 21.48. MS(ESI(+)) calcd for C_32_H_26_F_6_N_4_O^+^ [M + H]^+^: 597.21; found: 597.21.

(3a,8-bis(3-(trifluoromethyl)benzyl)-3,3a,8,8a-tetrahydropyrrolo[2,3-*b*]indol-1(2*H*)-yl)(pyrazin-2-yl)methanone (**b6**): Yellow oily, 86% Yield, ^1^H NMR (400 MHz, Acetone-*d*_6_), *δ*: 8.59–8.54 (m, 1H), 7.89 (t, *J* = 7.7 Hz, 1H), 7.70 (d, *J* = 7.8 Hz, 1H), 7.57–7.40 (m, 6H), 7.36–7.29 (m, 2H), 7.22–7.14 (m, 2H), 7.02–6.98 (m, 1H), 6.77 (d, *J* = 7.3 Hz, 1H), 6.10 (d, *J* = 7.8 Hz, 1H), 6.03 (s, 1H), 4.64 (d, *J* = 16.6 Hz, 1H), 4.50 (d, *J* = 16.6 Hz, 1H), 3.53–3.44 (m, 1H), 3.37 (d, *J* = 13.1 Hz, 1H), 3.21 (d, *J* = 13.2 Hz, 1H), 2.38–2.28 (m, 2H).^13^C NMR (100 MHz, Acetone-*d*_6_) δ 167.79, 154.62, 151.20, 148.52, 141.58, 139.69, 137.36, 134.23, 131.70 (q, *J* = 35.3 Hz), 130.96, 129.57, 129.14, 126.96, 126.92, 125.69, 125.53, 125.39, 124.32 (q, *J* = 268.2 Hz), 124.09, 123.72, 118.26, 106.59, 83.63, 56.95, 50.03, 49.22, 44.65, 38.46. MS(ESI(+)) calcd for C_31_H_24_F_6_N_4_O^+^ [M + H]^+^: 583.19; found: 583.19.

(3a,8-bis(3-(trifluoromethyl)benzyl)-3,3a,8,8a-tetrahydropyrrolo[2,3-*b*]indol-1(2*H*)-yl)(2-hydroxypyridin-3-yl)methanone (**b7**): White solid, Melting Point: 109–111 °C, 83% Yield, ^1^H NMR (400 MHz, Acetone-*d*_6_), *δ*: 11.17 (s, 1H), 7.57–7.47 (m, 6H), 7.38 (dd, *J* = 7.8, 4.4 Hz, 2H), 7.32–7.26 (m, 2H), 7.19–7.15 (m, 3H), 6.99–6.95 (m, 1H), 6.71 (dd, *J* = 9.5, 5.3 Hz, 1H), 6.28 (t, *J* = 6.7 Hz, 1H), 6.04 (d, *J* = 8.0 Hz, 1H), 5.89 (s, 1H), 4.59 (d, *J* = 16.7 Hz, 1H), 4.47 (d, *J* = 16.8 Hz, 1H), 3.40–3.35 (m, 1H), 3.16 (d, *J* = 13.2 Hz, 1H), 2.32–2.25 (m, 2H).^13^C NMR (100 MHz, Acetone-*d*_6_)δ 167.20, 160.00, 151.53, 142.95, 141.83, 141.63, 140.51, 139.91, 137.75, 137.60, 134.52, 134.38, 131.71 (q, *J* = 35.3 Hz), 131.36, 129.81, 129.26, 127.09, 127.05, 124.23 (q, *J* = 268.2 Hz), 124.02, 123.82, 118.25, 106.58, 105.52, 83.14, 57.72, 50.08, 47.97, 44.91, 38.87. MS(ESI(+)) calcd for C_32_H_25_F_6_N_3_O_2_^+^ [M + H]^+^: 598.19; found: 598.19.

(3a,8-bis(3-(trifluoromethyl)benzyl)-3,3a,8,8a-tetrahydropyrrolo[2,3-*b*]indol-1(2*H*)-yl)(2-chloropyridin-3-yl)methanone (**b8**): Yellow oily, 94% Yield, ^1^H NMR (400 MHz, Acetone-*d*_6_), *δ*: 8.45 (dd, *J* = 4.8, 1.9 Hz, 1H), 7.64 (d, *J* = 4.8 Hz, 1H), 7.57 (d, *J* = 9.9 Hz, 3H), 7.54–7.37 (m, 4H), 7.34 (d, *J* = 7.7 Hz, 1H), 7.25–7.13 (m, 3H), 7.03 (td, *J* = 7.7, 1.1 Hz, 1H), 6.75 (t, *J* = 7.4 Hz, 1H), 6.17 (d, *J* = 7.9 Hz, 1H), 5.96 (s, 1H), 4.66 (d, *J* = 16.7 Hz, 1H), 4.59 (d, *J* = 16.7 Hz, 1H), 3.39 (t, *J* = 2.5 Hz, 1H), 3.22 (d, *J* = 7.4 Hz, 2H), 2.39–2.34 (m, 2H).^13^C NMR (100 MHz, Acetone-*d*_6_ )δ 166.06, 151.47, 150.91, 146.99, 141.71, 139.68, 137.75, 134.48, 133.59, 131.66 (q, *J* = 35.3 Hz), 131.21, 130.44, 130.39, 130.07, 129.87, 129.47, 129.44, 127.20, 127.16, 124.34 (q, *J* = 268.2 Hz), 124.08, 124.04, 124.00, 123.81, 118.69, 106.80, 83.08, 58.03, 50.08, 48.15, 44.66, 38.63. MS(ESI(+)) calcd for C_32_H_24_ClF_6_N_3_O^+^ [M + H]^+^: 616.16; found: 616.16.

(3a,8-bis(3-(trifluoromethyl)benzyl)-3,3a,8,8a-tetrahydropyrrolo[2,3-*b*]indol-1(2*H*)-yl)(pyridin-2-yl)methanone (**b9**): Yellow oily, 92% Yield, ^1^H NMR (400 MHz, Acetone-*d*_6_), *δ*: 8.56–8.51 (m, 1H), 7.92–7.86 (m, 1H), 7.69 (dd, *J* = 7.9, 2.8 Hz, 1H), 7.57–7.54 (m, 2H), 7.50–7.31 (m, 6H), 7.24 (d, *J* = 6.2 Hz, 1H), 7.17 (d, *J* = 5.0 Hz, 2H), 7.02–6.99 (m, 1H), 6.77–6.71 (m, 1H), 6.11 (t, *J* = 8.2 Hz, 1H), 6.03 (d, *J* = 3.9 Hz, 1H), 4.67–4.61 (m, 1H), 4.50 (d, *J* = 16.7 Hz, 1H), 4.03–3.96 (m, 1H), 3.37 (d, *J* = 13.2 Hz, 1H), 3.20 (dd, *J* = 13.3, 2.6 Hz, 1H), 2.37–2.28 (m, 2H).^13^C NMR (100 MHz, Acetone-*d*_6_) δ 167.98, 154.78, 151.37, 148.70, 141.75, 139.86, 137.54, 134.40, 131.87 (q, *J* = 35.3 Hz), 131.13, 129.75, 129.32, 129.30, 127.13, 127.09, 125.87, 125.72, 125.58, 124.51 (q, *J* = 268.2 Hz), 124.26, 124.12, 124.09, 123.90, 118.44, 106.77, 83.80, 57.13, 50.20, 49.41, 44.83, 38.64. MS(ESI(+)) calcd for C_32_H_25_F_6_N_3_O^+^ [M + H]^+^: 582.20; found: 582.20.

(3a,8-bis(3-(trifluoromethyl)benzyl)-3,3a,8,8a-tetrahydropyrrolo[2,3-*b*]indol-1(2*H*)-yl)(pyridin-3-yl)methanone (**b10**): Yellow oily, 95% Yield, ^1^H NMR (400 MHz, Acetone-*d*_6_), *δ*: 8.67–8.60 (m, 2H), 7.83 (d, *J* = 7.9 Hz, 1H), 7.59–7.24 (m, 8H), 7.23–7.11 (m, 3H), 7.03 (td, *J* = 7.6, 1.0 Hz, 1H), 6.75 (t, *J* = 7.4 Hz, 1H), 6.14 (d, *J* = 7.9 Hz, 1H), 6.05 (s, 1H), 4.60 (d, *J* = 16.8 Hz, 1H), 4.50 (d, *J* = 16.8 Hz, 1H), 3.72–3.62 (m, 1H), 3.38 (d, *J* = 13.2 Hz, 1H), 3.20 (d, *J* = 13.2 Hz, 1H), 2.42–2.30 (m, 2H).^13^C NMR (100 MHz, Acetone-*d*_6_)δ 168.21, 151.56, 151.26, 148.94, 141.58, 139.61, 135.36, 134.18, 132.50, 131.39 (q, *J* = 35.3 Hz), 130.83, 130.50, 129.61, 129.25, 129.12, 126.89, 126.86, 126.32, 126.23, 125.06, 124.17 (q, *J* = 268.2 Hz), 123.78, 123.74, 123.52, 118.30, 106.55, 83.22, 57.28, 49.69, 49.62, 44.67, 38.48. MS(ESI(+)) calcd for C_32_H_25_F_6_N_3_O^+^ [M + H]^+^: 582.20; found: 582.20.

3a,8-bis(3-(trifluoromethyl)benzyl)-3,3a,8,8a-tetrahydropyrrolo[2,3-*b*]indol-1(2*H*)-yl)(6-chloropyridin-2-yl)methanone (**b11**): Yellow oily, 87%, ^1^H NMR (400 MHz, Acetone-*d*_6_), *δ*: 7.96 (t, *J* = 7.8 Hz, 1H), 7.70–7.67 (m, 1H), 7.56 (d, *J* = 7.7 Hz, 3H), 7.51–7.38 (m, 4H), 7.32 (t, *J* = 6.8 Hz, 1H), 7.24 (d, *J* = 8.3 Hz, 1H), 7.20–7.15 (m, 2H), 7.02 (dd, *J* = 7.8, 4.2 Hz, 1H), 6.74 (dd, *J* = 9.1, 3.9 Hz, 1H), 6.13–6.10 (m, 1H), 6.01 (d, *J* = 5.1 Hz, 1H), 4.63 (d, *J* = 16.7 Hz, 1H), 4.48 (d, *J* = 16.7 Hz, 1H), 3.54–3.46 (m, 1H), 3.36 (d, *J* = 13.2 Hz, 1H), 3.20 (d, *J* = 13.2 Hz, 1H), 2.41–2.30 (m, 2H).^13^C NMR (100 MHz, Acetone-*d*_6_)δ 166.37, 154.91, 151.28, 149.97, 141.70, 140.98, 139.80, 134.39, 131.78 (q, *J* = 35.3 Hz), 131.10, 129.77, 129.59, 129.40, 129.31, 127.13, 127.09, 126.47, 124.29 (q, *J* = 268.2 Hz), 124.07, 123.93, 123.46, 118.57, 106.88, 83.97, 57.16, 50.25, 49.47, 44.76, 38.47. MS(ESI(+)) calcd for C_32_H_24_ClF_6_N_3_O^+^ [M + H]^+^: 616.16; found: 616.16.

(3a,8-bis(3-(trifluoromethyl)benzyl)-3,3a,8,8a-tetrahydropyrrolo[2,3-*b*]indol-1(2*H*)-yl)(6-methylpyridin-3-yl)methanone (**b12**): White solid, Melting Point: 117–119 °C, 89% Yield, ^1^H NMR (400 MHz, Acetone-*d*_6_), *δ*: 8.53 (s, 1H), 7.72 (dd, *J* = 8.0, 1.9 Hz, 1H), 7.57–7.29 (m, 7H), 7.27–7.12 (m, 4H), 7.05–7.00 (m, 1H), 6.75 (t, *J* = 7.4 Hz, 1H), 6.12 (d, *J* = 7.9 Hz, 1H), 6.05 (s, 1H), 4.58 (d, *J* = 16.8 Hz, 1H), 4.49 (d, *J* = 16.8 Hz, 1H), 3.47 (td, *J* = 11.1, 6.0 Hz, 1H), 3.37 (d, *J* = 13.2 Hz, 1H), 3.20 (d, *J* = 13.2 Hz, 1H), 2.50 (s, 3H), 2.35–2.31 (m, 2H).^13^C NMR (100 MHz, Acetone-*d*_6_) δ 168.61, 160.96, 151.46, 148.65, 141.77, 139.82, 136.01, 134.37, 131.60 (q, *J* = 35.3 Hz), 131.02, 130.68, 130.37, 130.27, 129.79, 129.43, 129.30, 127.09, 127.05, 126.51, 126.42, 124.35 (q, *J* = 268.2 Hz), 123.96, 123.92, 123.72, 122.92, 118.45, 106.72, 83.37, 57.42, 49.86, 44.87, 38.67, 24.28. MS(ESI(+)) calcd for C_33_H_27_F_6_N_3_O^+^ [M + H]^+^: 596.21; found: 596.21.

(3a,8-bis(3-(trifluoromethyl)benzyl)-3,3a,8,8a-tetrahydropyrrolo[2,3-*b*]indol-1(2*H*)-yl)(4-methoxyphenyl)methanone(**b13**): Yellow oily, 90% Yield, ^1^H NMR (400 MHz, Acetone-*d*_6_), *δ*: 7.54 (t, *J* = 6.5 Hz, 2H), 7.51–7.34 (m, 6H), 7.31 (d, *J* = 7.6 Hz, 1H), 7.22–7.15 (m, 2H), 7.10 (d, *J* = 6.0 Hz, 1H), 7.04–7.00 (m, 1H), 6.93 (d, *J* = 8.5 Hz, 2H), 6.74 (td, *J* = 7.5, 0.8 Hz, 1H), 6.09 (d, *J* = 7.0 Hz, 2H), 4.52 (dd, *J* = 2.3, 0.7 Hz, 2H), 3.82 (s, 3H), 3.71 (d, *J* = 0.6 Hz, 1H), 3.36 (d, *J* = 11.2 Hz, 1H), 3.19 (d, *J* = 13.2 Hz, 1H), 2.32 (d, *J* = 6.4 Hz, 2H).^13^C NMR (100 MHz, Acetone-*d*_6_)δ 170.21, 162.02, 151.52, 141.76, 139.91, 134.39, 131.73 (q, *J* = 35.3 Hz), 131.04, 130.67, 130.31, 129.95, 129.77, 129.36, 129.28, 128.95, 127.10, 127.07, 126.53, 126.44, 124.33 (q, *J* = 268.2 Hz), 123.97, 123.93, 123.89, 123.85, 118.34, 113.91, 106.66, 83.21, 57.28, 55.54, 50.18, 49.68, 44.91, 38.73. MS(ESI(+)) calcd for C_34_H_28_F_6_N_2_O_2_^+^ [M + H]^+^: 611.21; found: 611.21.

(3a,8-bis(3-(trifluoromethyl)benzyl)-3,3a,8,8a-tetrahydropyrrolo[2,3-*b*]indol-1(2*H*)-yl)(5-methylpyridin-3-yl)methanone (**b14**): Yellow oily, 93% Yield, ^1^H NMR (400 MHz, Acetone-*d*_6_), *δ*: 8.49–8.41 (m, 2H), 7.67–7.35 (m, 7H), 7.32 (d, *J* = 7.7 Hz, 1H), 7.22–7.14 (m, 3H), 7.03 (td, *J* = 7.7, 1.1 Hz, 1H), 6.75 (t, *J* = 7.4 Hz, 1H), 6.14 (d, *J* = 7.9 Hz, 1H), 6.05 (s, 1H), 4.60 (d, *J* = 16.8 Hz, 1H), 4.49 (d, *J* = 16.8 Hz, 1H), 3.45 (td, *J* = 11.0, 6.3 Hz, 1H), 3.38 (d, *J* = 13.2 Hz, 1H), 3.20 (d, *J* = 13.2 Hz, 1H), 2.37–2.31 (m, 5H).^13^C NMR (100 MHz, Acetone-*d*_6_)δ 168.54, 152.14, 151.45, 146.25, 141.83, 139.82, 135.69, 134.38, 133.44, 132.24, 131.61 (q, *J* = 35.3 Hz), 131.07, 129.82, 129.44, 129.32, 127.10, 127.06, 126.53, 126.43, 124.36 (q, *J* = 268.2 Hz), 123.93, 118.47, 106.70, 83.38, 57.47, 49.89, 49.79, 44.87, 38.67, 17.94. MS(ESI(+)) calcd for C_33_H_27_F_6_N_3_O^+^ [M + H]^+^: 596.21; found: 596.21.

(3a,8-bis(3-(trifluoromethyl)benzyl)-3,3a,8,8a-tetrahydropyrrolo[2,3-*b*]indol-1(2*H*)-yl)(6-chloropyridin-3-yl)methanone (**b15**): Yellow oily, 94% Yield, ^1^H NMR (400 MHz, DMSO- *d*_6_), *δ*: 8.47 (d, *J* = 2.0 Hz, 1H), 7.94 (dd, *J* = 8.3, 2.4 Hz, 1H), 7.63–7.50 (m, 3H), 7.47 (s, 1H), 7.40 (td, *J* = 7.7, 3.7 Hz, 2H), 7.29 (d, *J* = 7.7 Hz, 1H), 7.23 (d, *J* = 6.7 Hz, 1H), 7.12 (s, 1H), 7.08–6.98 (m, 2H), 6.75 (t, *J* = 7.3 Hz, 1H), 6.12 (d, *J* = 7.8 Hz, 1H), 5.93 (s, 1H), 4.46–4.35 (m, 2H), 3.57 (dd, *J* = 10.6, 6.6 Hz, 1H), 3.38 (d, *J* = 7.9 Hz, 2H), 3.14 (d, *J* = 13.2 Hz, 1H), 2.28 (dt, *J* = 11.8, 7.0 Hz, 2H).^13^C NMR (100 MHz, DMSO- *d*_6_) δ 166.74, 152.13, 150.76, 149.08, 141.11, 139.37, 139.28, 134.13, 131.40 (q, *J* = 35.3 Hz), 131.15, 130.67, 129.72, 129.59, 129.28, 129.16, 128.77, 126.50, 126.06, 126.00, 124.53 (q, *J* = 268.2 Hz), 124.18, 123.81, 123.60, 123.50, 118.08, 106.19, 82.63, 56.92, 49.33, 49.18, 44.04, 38.37. MS(ESI(+)) calcd for C_32_H_24_ClF_6_N_3_O^+^ [M + H]^+^: 616.16; found: 616.16.

(3a,8-bis(3-(trifluoromethyl)benzyl)-3,3a,8,8a-tetrahydropyrrolo[2,3-*b*]indol-1(2*H*)-yl)(5,6-dichloropyridin-3-yl)methanone (**b16**): Yellow oily, Yield, ^1^H NMR (400 MHz, Acetone-*d*_6_), *δ*: 8.39 (d, *J* = 1.9 Hz, 1H), 8.00 (d, *J* = 1.9 Hz, 1H), 7.59–7.32 (m, 6H), 7.28 (d, *J* = 7.7 Hz, 1H), 7.18–7.10 (m, 3H), 7.04–6.98 (m, 1H), 6.73 (t, *J* = 7.4 Hz, 1H), 6.14 (d, *J* = 7.9 Hz, 1H), 5.99 (s, 1H), 4.55 (d, *J* = 16.8 Hz, 1H), 4.46 (d, *J* = 16.8 Hz, 1H), 3.76–3.70 (m, 1H), 3.35 (d, *J* = 13.2 Hz, 1H), 3.16 (d, *J* = 13.2 Hz, 1H), 2.38–2.28 (m, 2H).^13^C NMR (100 MHz, Acetone-*d*_6_)δ 165.74, 151.21, 149.98, 146.79, 141.55, 139.51, 138.46, 134.15, 133.19, 131.27 (q, *J* = 35.3 Hz), 130.86, 130.55, 130.51, 130.17, 130.11, 129.65, 129.30, 129.15, 127.39, 126.88, 126.85, 124.18 (q, *J* = 268.2 Hz), 123.80, 123.77, 118.39, 106.60, 83.36, 57.36, 49.66, 49.49, 44.63, 38.43. MS(ESI(+)) calcd for C_32_H_23_Cl_2_F_6_N_3_O^+^ [M + H]^+^: 650.12; found: 650.12.

(3a,8-bis(3-(trifluoromethyl)benzyl)-3,3a,8,8a-tetrahydropyrrolo[2,3-*b*]indol-1(2*H*)-yl)(5-chloropyrazin-2-yl)methanone (**b17**): Yellow oily, 83% Yield, ^1^H NMR (400 MHz, Acetone-*d*_6_), *δ*: 8.76–8.60 (m, 2H), 7.57–7.47 (m, 3H), 7.47–7.33 (m, 3H), 7.31 (d, *J* = 7.5 Hz, 1H), 7.24–7.15 (m, 3H), 7.05–7.00 (m, 1H), 6.75 (td, *J* = 7.4, 0.7 Hz, 1H), 6.13 (d, *J* = 7.8 Hz, 1H), 6.02 (s, 1H), 4.63 (d, *J* = 16.7 Hz, 1H), 4.47 (d, *J* = 16.7 Hz, 1H), 3.50 (dt, *J* = 11.6, 3.6 Hz, 1H), 3.39–3.33 (m, 1H), 3.23–3.18 (m, 1H), 2.36 (dd, *J* = 12.1, 5.7 Hz, 2H).^13^C NMR (100 MHz, Acetone-*d*_6_) δ 165.24, 151.21, 150.49, 148.14, 145.66, 143.10, 141.62, 139.72, 134.40, 131.71 (q, *J* = 35.3 Hz), 131.09, 129.80, 129.44, 129.35, 127.13, 127.09, 124.31 (q, *J* = 268.2 Hz), 124.03, 124.00, 123.96, 123.93, 118.66, 106.89, 84.01, 57.20, 50.28, 49.35, 44.70, 38.42. MS(ESI(+)) calcd for C_31_H_23_ClF_6_N_4_O^+^ [M + H]^+^: 617.15; found: 617.15.

(3a,8-bis(3-(trifluoromethyl)benzyl)-3,3a,8,8a-tetrahydropyrrolo[2,3-*b*]indol-1(2*H*)-yl)(2-fluoropyridin-3-yl)methanone (**b18**): Yellow oily, 88% Yield, ^1^H NMR (400 MHz, Acetone-*d*_6_), *δ*: 8.30 (d, *J* = 4.9 Hz, 1H), 7.96–7.83 (m, 1H), 7.54 (dd, *J* = 14.4, 7.1 Hz, 3H), 7.44–7.37 (m, 3H), 7.36–7.06 (m, 5H), 7.02 (dd, *J* = 14.0, 6.4 Hz, 1H), 6.75 (t, *J* = 7.4 Hz, 1H), 6.11 (d, *J* = 7.9 Hz, 1H), 5.96 (s, 1H), 4.62 (d, *J* = 16.7 Hz, 1H), 4.46 (d, *J* = 16.7 Hz, 1H), 3.60–3.50 (m, 1H), 3.42–3.37 (m, 1H), 3.25–3.16 (m, 1H), 2.44–2.32 (m, 2H).^13^C NMR (100 MHz, Acetone-*d*_6_) δ 164.57, 160.66, 158.30, 151.45, 149.88, 149.73(CH, d, *J* = 13.0 Hz ), 141.64, 141.14, 141.10, 139.73, 134.36, 131.53 (q, *J* = 35.3 Hz), 131.01, 129.81, 129.48, 129.34, 127.08, 127.04, 124.36 (q, *J* = 268.2 Hz), 124.00, 122.74, 122.70, 120.24(d, *J* = 31.9 Hz ), 119.91, 118.67, 106.86, 83.43, 57.85, 50.23, 48.50, 44.84, 38.42. MS(ESI(+)) calcd for C_32_H_24_F_7_N_3_O^+^ [M + H]^+^: 600.19; found: 600.19.

3a,8-bis(3-(trifluoromethyl)benzyl)-3,3a,8,8a-tetrahydropyrrolo[2,3-*b*]indol-1(2*H*)-yl)(pyridin-4-yl)methanone(**b19**): Yellow oily, 90% Yield, ^1^H NMR (400 MHz, DMSO- *d*_6_), *δ*: 8.64 (dd, *J* = 4.5, 1.4 Hz, 2H), 7.53 (d, *J* = 7.7 Hz, 2H), 7.45 (s, 1H), 7.40–7.33 (m, 4H), 7.27 (d, *J* = 7.6 Hz, 1H), 7.20 (d, *J* = 7.3 Hz, 1H), 7.10 (s, 1H), 7.00 (d, *J* = 6.9 Hz, 2H), 6.72 (t, *J* = 7.4 Hz, 1H), 6.08 (d, *J* = 7.8 Hz, 1H), 5.88 (s, 1H), 4.44 (d, *J* = 16.9 Hz, 2H), 3.45 (dd, *J* = 10.5, 6.2 Hz, 1H), 3.29 (d, *J* = 5.4 Hz, 2H), 3.13 (d, *J* = 13.1 Hz, 1H), 2.29–2.19 (m, 2H).^13^C NMR (100 MHz, DMSO- *d*_6_)δ 167.84, 150.74, 150.46, 143.56, 141.13, 139.39, 134.15, 131.17 (q, *J* = 35.3 Hz), 130.68, 129.75, 129.61, 129.30, 129.17, 129.09, 128.78, 126.51, 126.08, 126.01, 124.18 (q, *J* = 268.2 Hz), 123.83, 123.63, 123.44, 123.31, 121.83, 118.11, 106.19, 82.65, 56.98, 49.28, 49.12, 43.98, 38.33. MS(ESI(+)) calcd for C_32_H_25_F_6_N_3_O^+^ [M + H]^+^: 582.20; found: 582.20.

### 3.3. Biological Activity

The antifungal activity of *Chimonanthus praecox* analogues was measured according to the previously reported method [17,18].

The six phytopathogenic fungi (*Verticillium dahliae*-ACCC 36211; *Colletotrichum orbiculare*-SAUM 0321; *Cytospora juglandis*-ACCC36357; *Curvularia lunata*-SAUM 1373; *Sclerotinia sclerotiorum*-ACCC34236; *Altenaria solani*-SAUM 1275) were provided by the School of Environmental and Chemical Engineering, Jiangsu University of Science and Technology. The antifungal concentrations were 250, 125, 62.5, 32.3, 15.63, 7.8, 3.9, and 1.95 µg mL^−1^, respectively. The antifungal test plates were incubated aerobically at 28 °C for 48 h. The MICs were examined. All tests were performed in triplicate and repeated if the results differed.

## 4. Conclusions

In summary, a total of 39 *Chimonanthus praecox* alkaloids (**a1**~**a20** and **b1**~**b19**) were synthesized from indole-3-acetonitrile via alkylation reaction, reduction reaction, and acylation reaction. Their antifungal activities were studied, and the relationship between the antifungal activity of the target compounds and their structures was discussed. Compound **b15** showed the best inhibitory effect on *A. solani*, and its minimum inhibitory concentration (MIC) value is 1.95 µg mL^−1^; compound **b17** displayed the best effect on *S. sclerotiorum*, and its minimum inhibitory concentration (MIC) value is 1.95 µg mL^−1^. These results will lay a foundation for subsequent research and development.

## Figures and Tables

**Table 1:** MIC of *Chimonanthus praecox* derivatives against 6 plant pathogens.

Compd.	MIC （µg mL^−1^）					
	*S. s.*	*A. s.*	*V. d.*	*C. o.*	*C. j.*	*C. l.*
**a1**	62.5	-	125	32.3	-	31.3
**a2**	31.2	-	31.25	15.61	32.25	125
**a3**	62.5	-	31.25	15.61	31.25	31.25
**a4**	62.5	250	62.5	250	125	125
**a5**	31.25	62.5	125	250	250	125
**a6**	31.3	15.61	62.5	62.5	62.5	31.3
**a7**	250	-	250	250	125	250
**a8**	125	250	62.5	125	-	250
**a9**	62.5	250	250	250	-	250
**a10**	62.5	62.5	31.25	15.16	-	31.25
**a11**	31.25	-	62.5	250	-	125
**a12**	62.5	31.3	62.5	250	125	125
**a13**	62.5	-	62.5	125	31.25	125
**a14**	-	-	250	250	-	250
**a15**	31.25	-	31.25	62.5	15.61	31.25
**a16**	15.63	125	31.25	62.5	31.25	125
**a17**	125	250	62.5	250	250	125
**a18**	62.5	250	125	62.5	250	125
**a19**	7.8	31.25	31.25	62.5	-	62.5
**a20**	31.25	125	62.5	31.25	62.5	-
**b1**	125	62.5	31.3	250	62.5	62.5
**b2**	62.5	-	-	-	125	125
**b3**	62.5	62.5	-	-	125	62.5
**b4**	31.3	31.3	62.5	62.5	31.25	62.5
**b5**	62.5	125	125	-	-	31.3
**b6**	31.3	62.5	-	31.3	125	62.5
**b7**	62.5	62.5	-	125	31.3	62.5
**b8**	31.3	15.61	125	62.5	62.5	62.5
**b9**	31.3	62.5	-	62.5	125	62.5
**b10**	31.3	15.61	62.5	31.3	15.61	31.3
**b11**	15.61	31.3	15.61	62.5	125	-
**b12**	7.81	3.91	125	31.3	31.3	31.3
**b13**	62.5	62.5	-	31.3	125	250
**b14**	62.5	125	125	-	31.3	31.3
**b15**	7.81	1.95	125	31.3	-	15.61
**b16**	3.91	3.91	125	15.61	-	15.61
**b17**	1.95	3.91	62.5	15.61	31.3	15.61
**b18**	15.61	31.3	-	62.5	62.5	125
**b19**	31.25	15.63	125	62.5	125	15.61
**C**	7.8	62.5	15.16	62.5	31.3	125
**A**	3.9	15.16	62.5	31.3	125	62.5

Note: The Carbendazim and Amphotericin B were used as the positive controls; “-” means no inhibition effect. MIC: Minimal Inhibitory Concentration; *S. s.: Sclerotinia sclerotiorum; A. s.: Altenaria solani; V. d.: Verticillium dahliae; C. o.: Colletotrichum orbiculare; C. j.: Cytospora juglandis; C. l.: Curvularia lunata;* C: Carbendazim; A: Amphotericin B.

## Data Availability

Data are contained within the article or the Supplementary Materials file.

## Supplementary Materials

The following supporting information can be downloaded at: https://www.mdpi.com/article/10.3390/molecules27175570/s1, Figure S1: ^1^H-NMR spectroscopic data of compound **a1**; Figure S2: ^13^C-NMR spectroscopic data of compound **a1**; Figure S3: ^1^H-NMR spectroscopic data of compound **a2**; Figure S4: ^13^C-NMR spectroscopic data of compound **a2**; Figure S5: ^1^H-NMR spectroscopic data of compound **a3**; Figure S6: ^13^C-NMR spectroscopic data of compound **a3**; Figure S7: ^1^H-NMR spectroscopic data of compound **a4**; Figure S8: ^13^C-NMR spectroscopic data of compound **a4**; Figure S9: ^1^H-NMR spectroscopic data of compound **a5**; Figure S10**:** ^13^C-NMR spectroscopic data of compound **a5**; Figure S11: ^1^H-NMR spectroscopic data of compound **a6**; Figure S12: ^13^C-NMR spectroscopic data of compound **a6**; Figure S13: ^1^H-NMR spectroscopic data of compound **a7**; Figure S14: ^13^C-NMR spectroscopic data of compound **a7**; Figure S15: 1H-NMR spectroscopic data of compound **a8**;Figure S16: 13C-NMR spectroscopic data of compound **a8**; Figure S17: 1H-NMR spectroscopic data of compound **a9**; Figure S18: 13C-NMR spectroscopic data of compound **a9**; Figure S19: 1H-NMR spectroscopic data of compound **a10**; Figure S20: 13C-NMR spectroscopic data of compound **a10**; Figure S21: 1H-NMR spectroscopic data of compound **a11**; Figure S22: 13C-NMR spectroscopic data of compound **a11**; Figure S23: 1H-NMR spectroscopic data of compound **a12**; Figure S24: 13C-NMR spectroscopic data of compound **a12**; Figure S25: 1H-NMR spectroscopic data of compound **a13**; Figure S26: 13C-NMR spectroscopic data of compound **a13**; Figure S27: 1H-NMR spectroscopic data of compound **a14**; Figure S28: 13C-NMR spectroscopic data of compound **a14**; Figure S29: 1H-NMR spectroscopic data of compound **a15**; Figure S30: 13C-NMR spectroscopic data of compound **a15**; Figure S31: 1H-NMR spectroscopic data of compound **a16**; Figure S32: 13C-NMR spectroscopic data of compound **a16**; Figure S33: 1H-NMR spectroscopic data of compound **a17**; Figure S34: 13C-NMR spectroscopic data of compound **a17**; Figure S35: 1H-NMR spectroscopic data of compound **a18**; Figure S36: 13C-NMR spectroscopic data of compound **a18**; Figure S37: 1H-NMR spectroscopic data of compound **a19**; Figure S38: 13C-NMR spectroscopic data of compound **a19**; Figure S39: 1H-NMR spectroscopic data of compound **a20**; Figure S40: 13C-NMR spectroscopic data of compound **a20**; Figure S41: 1H-NMR spectroscopic data of compound **b1**; Figure S42: 13C-NMR spectroscopic data of compound **b1**; Figure S43: 1H-NMR spectroscopic data of compound **b2**; Figure S44: 13C-NMR spectroscopic data of compound **b2**; Figure S45: 1H-NMR spectroscopic data of compound **b3**; Figure S46: 13C-NMR spectroscopic data of compound **b3**; Figure S47: 1H-NMR spectroscopic data of compound **b4**; Figure S48: 13C-NMR spectroscopic data of compound **b4**; Figure S49: 1H-NMR spectroscopic data of compound **b5**; Figure S50: 13C-NMR spectroscopic data of compound **b5**; Figure S51: 1H-NMR spectroscopic data of compound **b6**; Figure S52: 13C-NMR spectroscopic data of compound **b6**; Figure S53: 1H-NMR spectroscopic data of compound **b7**; Figure S54: 13C-NMR spectroscopic data of compound **b7**; Figure S55: 1H-NMR spectroscopic data of compound **b8**; Figure S56: 13C-NMR spectroscopic data of compound **b8**; Figure S57: 1H-NMR spectroscopic data of compound **b9**; Figure S58: 13C-NMR spectroscopic data of compound **b9**; Figure S59: 1H-NMR spectroscopic data of compound **b10**; Figure S60: 13C-NMR spectroscopic data of compound **b10**; Figure S61: 1H-NMR spectroscopic data of compound **b11**; Figure S62: 13C-NMR spectroscopic data of compound **b11**; Figure S63: 1H-NMR spectroscopic data of compound **b12**; Figure S64: 13C-NMR spectroscopic data of compound **b12**; Figure S65: 1H-NMR spectroscopic data of compound **b13**; Figure S66: 13C-NMR spectroscopic data of compound **b13**; Figure S67: 1H-NMR spectroscopic data of compound **b14**; Figure S68: 13C-NMR spectroscopic data of compound **b14**; Figure S69: 1H-NMR spectroscopic data of compound **b15**; Figure S70: 13C-NMR spectroscopic data of compound **b15**; Figure S71: 1H-NMR spectroscopic data of compound **b16**; Figure S72: 13C-NMR spectroscopic data of compound **b16**; Figure S73: 1H-NMR spectroscopic data of compound **b17**; Figure S74: 13C-NMR spectroscopic data of compound **b17**; Figure S75: 1H-NMR spectroscopic data of compound **b18**; Figure S76: 13C-NMR spectroscopic data of compound **b18**; Figure S77: 1H-NMR spectroscopic data of compound **b19**; Figure S78: 13C-NMR spectroscopic data of compound **b19**.

## Sample Availability

Samples of the compounds are available from the authors.
