# Supplementary material for: Synthesis and Antifungal Activity of Chimonanthus praecox Derivatives
Source: Molecules. 2022 Aug 30;27(17):5570. doi: 10.3390/molecules27175570 (PMC9457717; doi:10.3390/molecules27175570)
Supplement: Supplementary file 1 [file molecules-27-05570-s001.zip › molecules-1664114-supplementary.pdf]

Supplementary Materials: Synthesis and antifungal activity of cyclotryptamine derivatives

Jinfeng Chen [#], Yimou Yang [#], Yujie Zhou, Yang Wei, Rui Zhu, Shaojun Zheng [*]

[a] School of Environmental and Chemical Engineering, Jiangsu University of Science and Technology, Zhenjiang 212003, Jiangsu, China.
* Correspondence: ; sz281cam@just.edu.cn (S.Z.);

To search efficient agricultural antifungal lead compounds, 39 cyclotryptamine derivatives were designed, synthesized and evaluated for their antifungal activities. The structures of target compounds were fully characterized by ^{1}H NMR, ^{13}C NMR, and MS spectra. The preliminary bioassays revealed that some compounds exhibited excellent antifungal activity in vitro. For example, the minimum inhibitory concentration (MIC) of compound **b15** against *Phytophthora infestans* was 1.95 μg mL^{-1}, and the minimum inhibitory concentration (MIC) of compound **b17** against *Sclerotinia sclerotiorum* was 1.95 μg mL^{-1}. Therefore, compounds **b15** and **b17** were identified as the most promising candidates for further study.

Keywords: cyclotryptamines derivative; synthesis; antifungal activity; structure

activity relationship

1. **^{1}H- and ^{13}C-NMR Spectra**

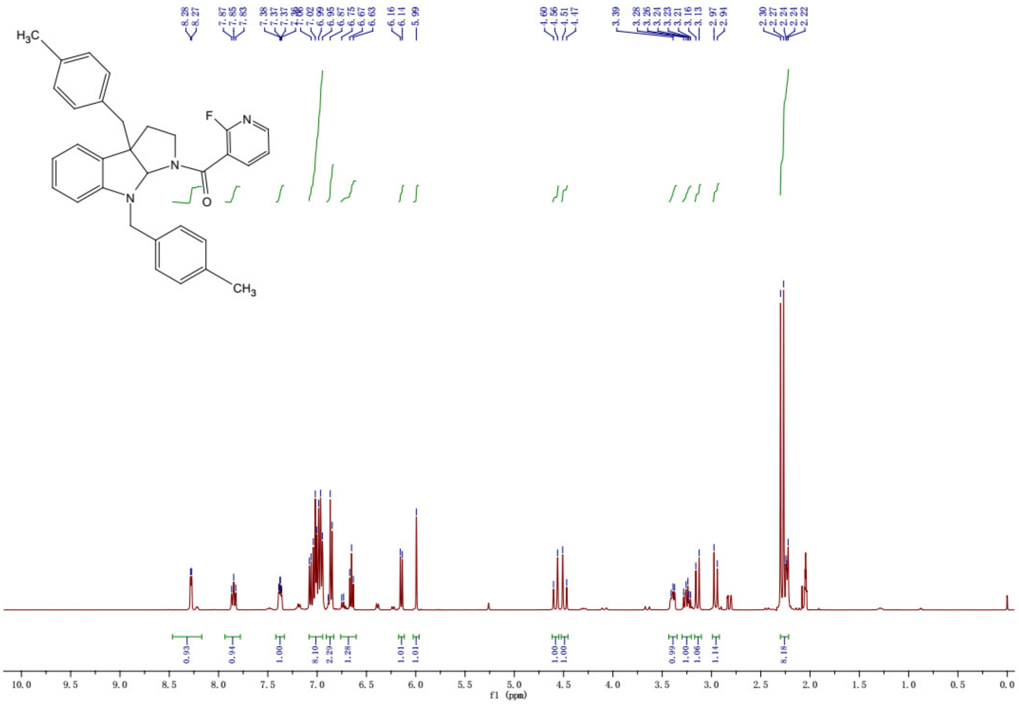

Figure S1 ^{1}H-NMR spectroscopic data of compound **a1**

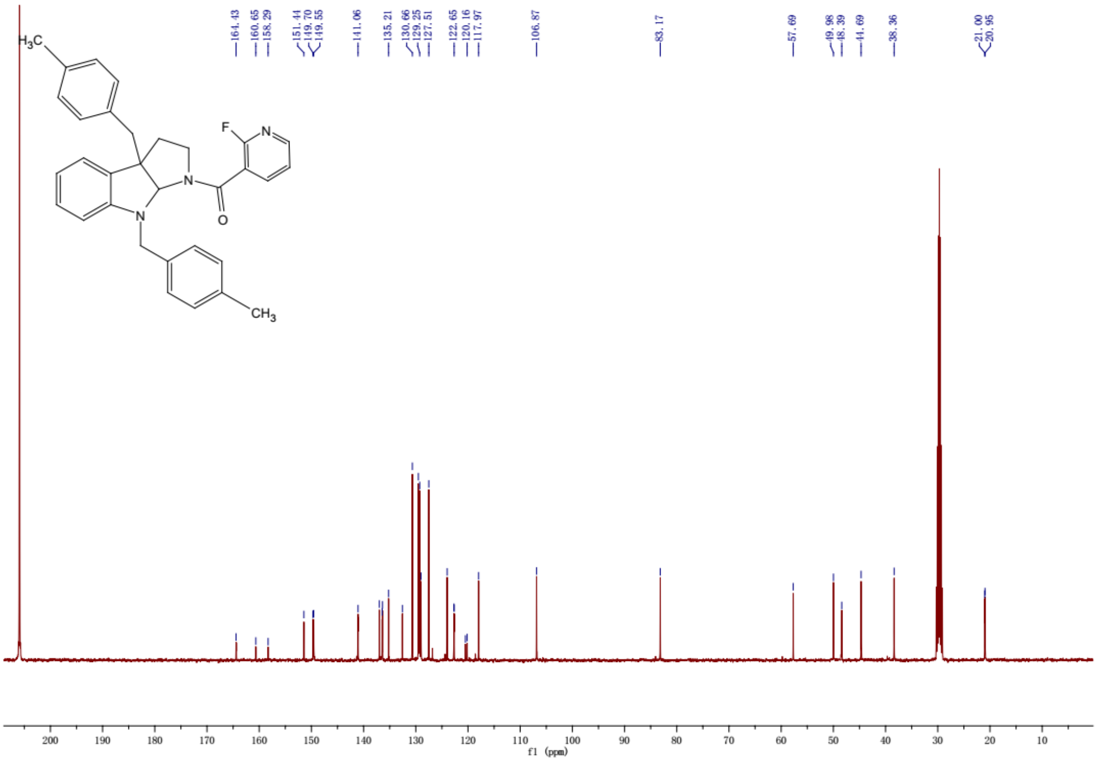

Figure S2 ^{13}C-NMR spectroscopic data of compound **a1**

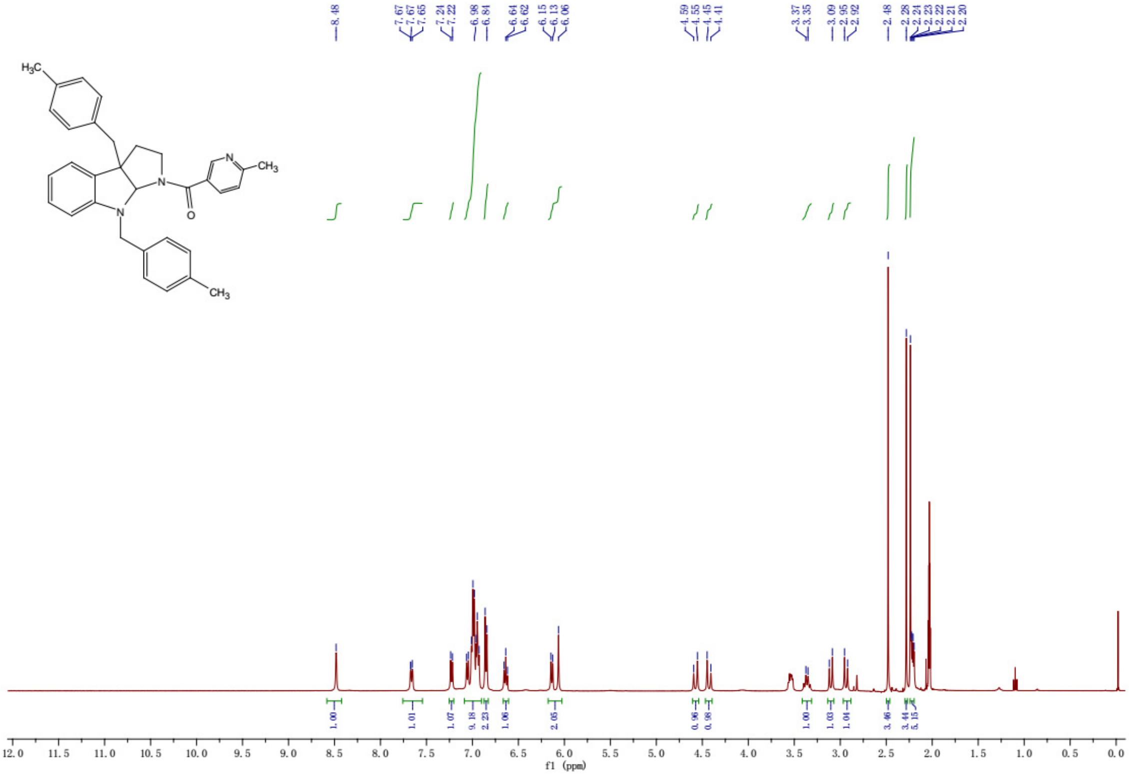

Figure S3 ^{1}H-NMR spectroscopic data of compound **a2** 的 ^{1}H-NMR

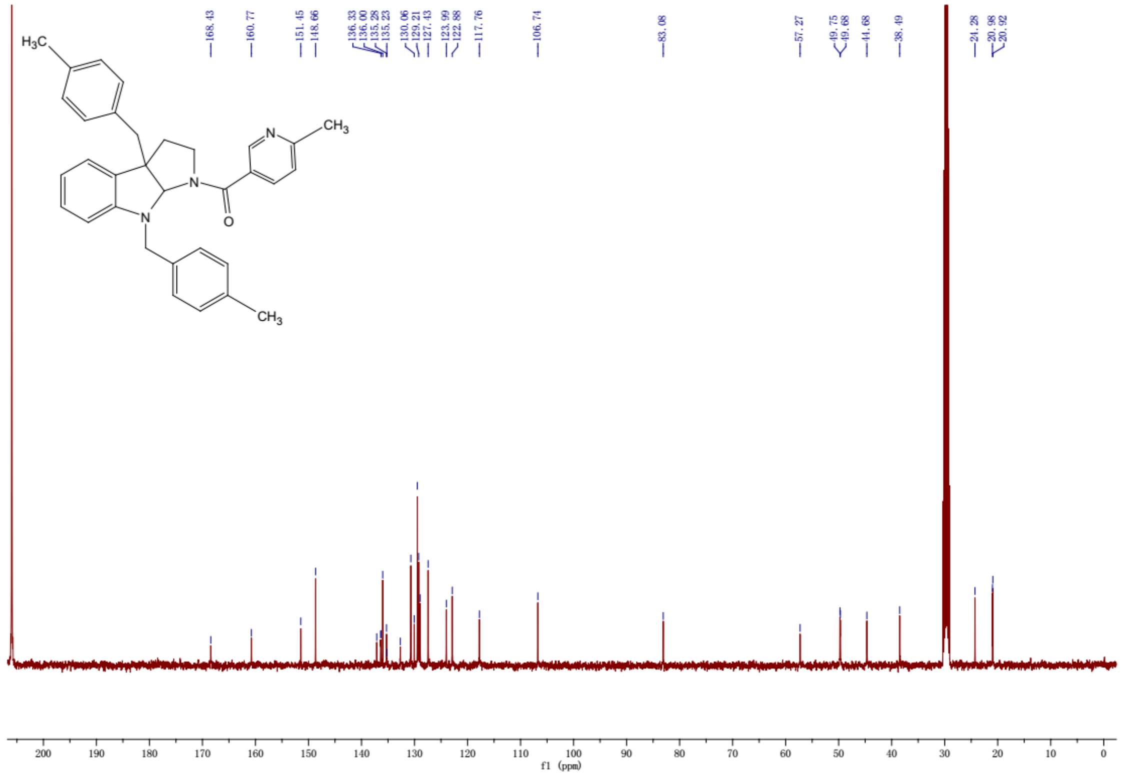

Figure S4 ^{13}C-NMR spectroscopic data of compound **a2**

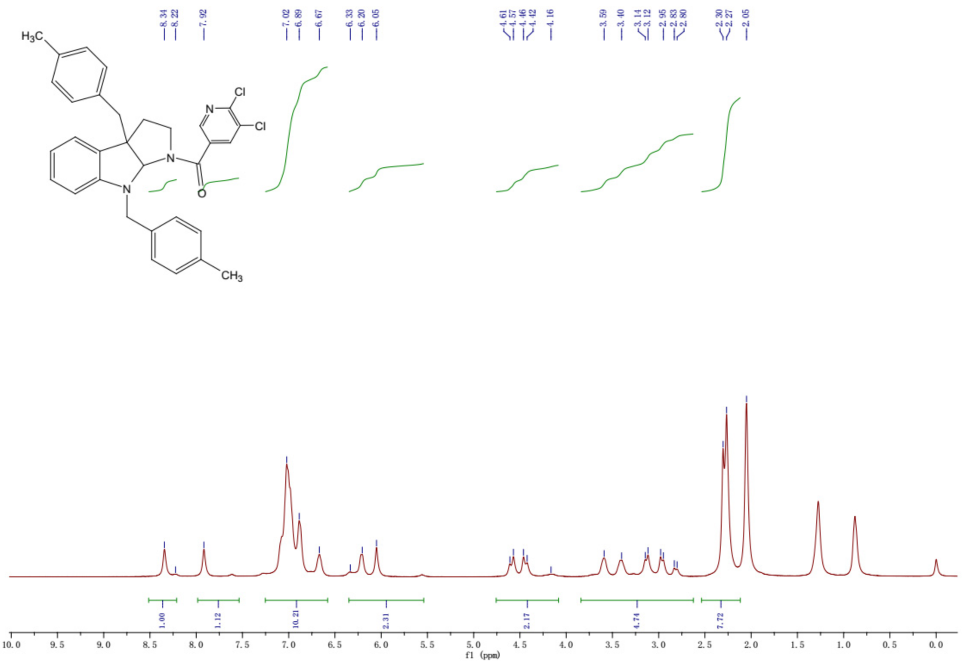

Figure S5 ^{1}H-NMR spectroscopic data of compound **a3**

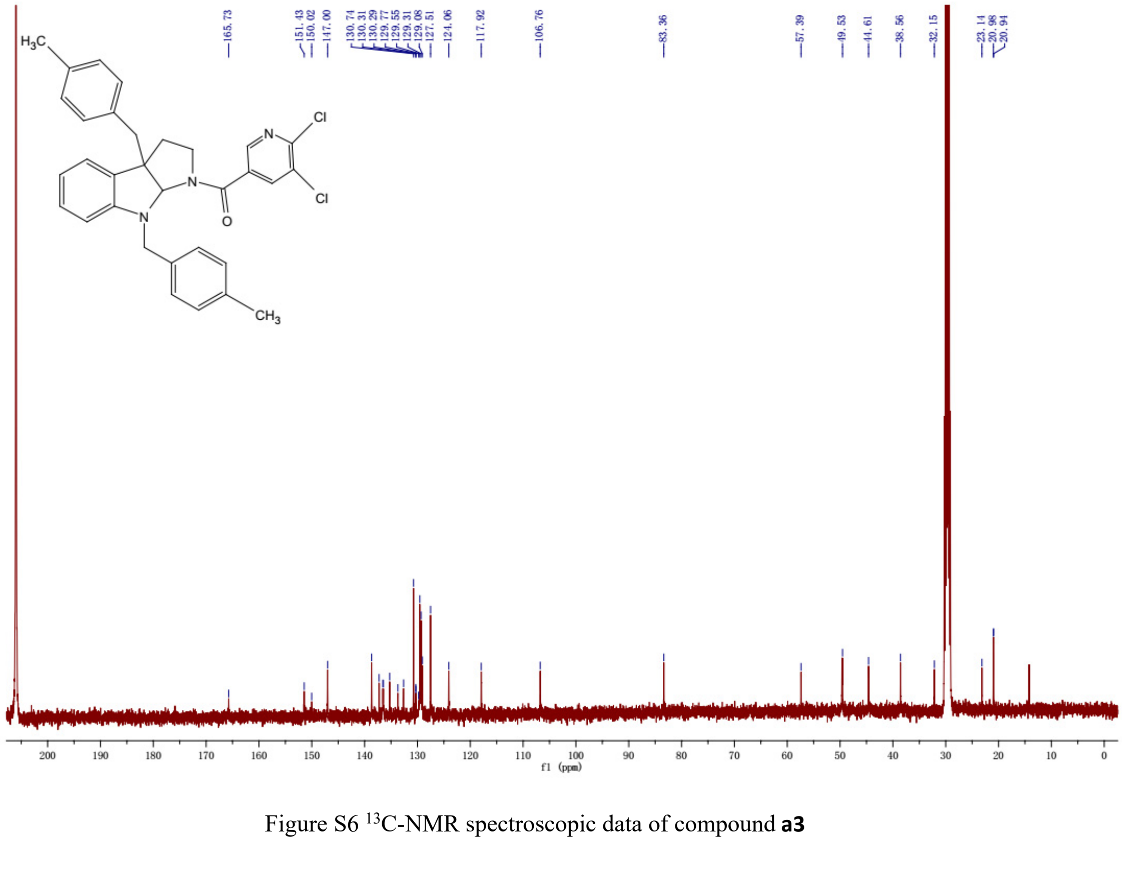

Figure S6 ^{13}C-NMR spectroscopic data of compound **a3**

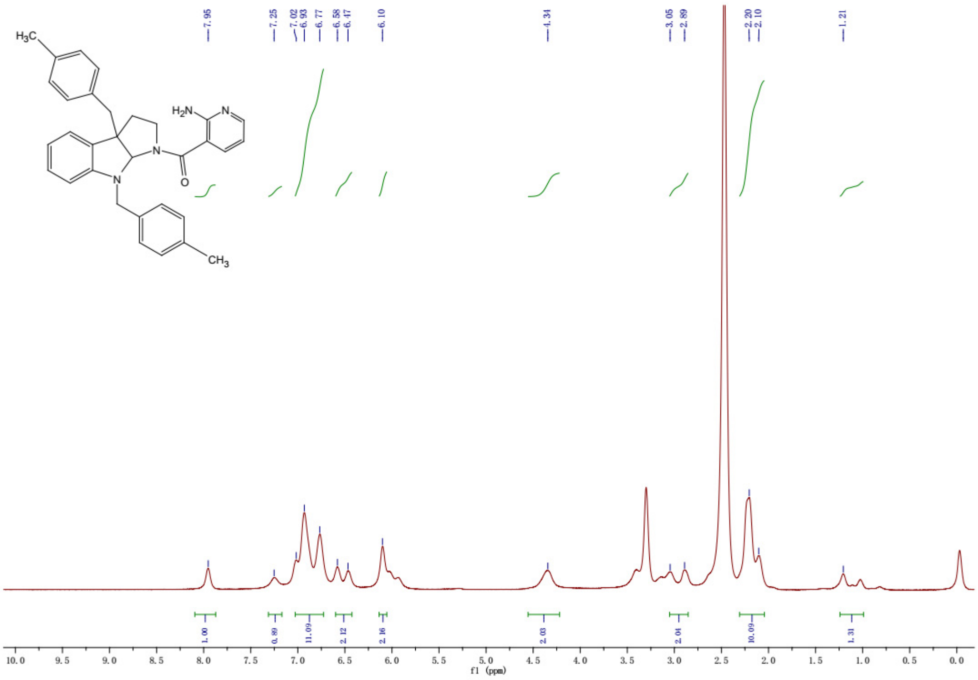

Figure S7 ^{1}H-NMR spectroscopic data of compound **a4**

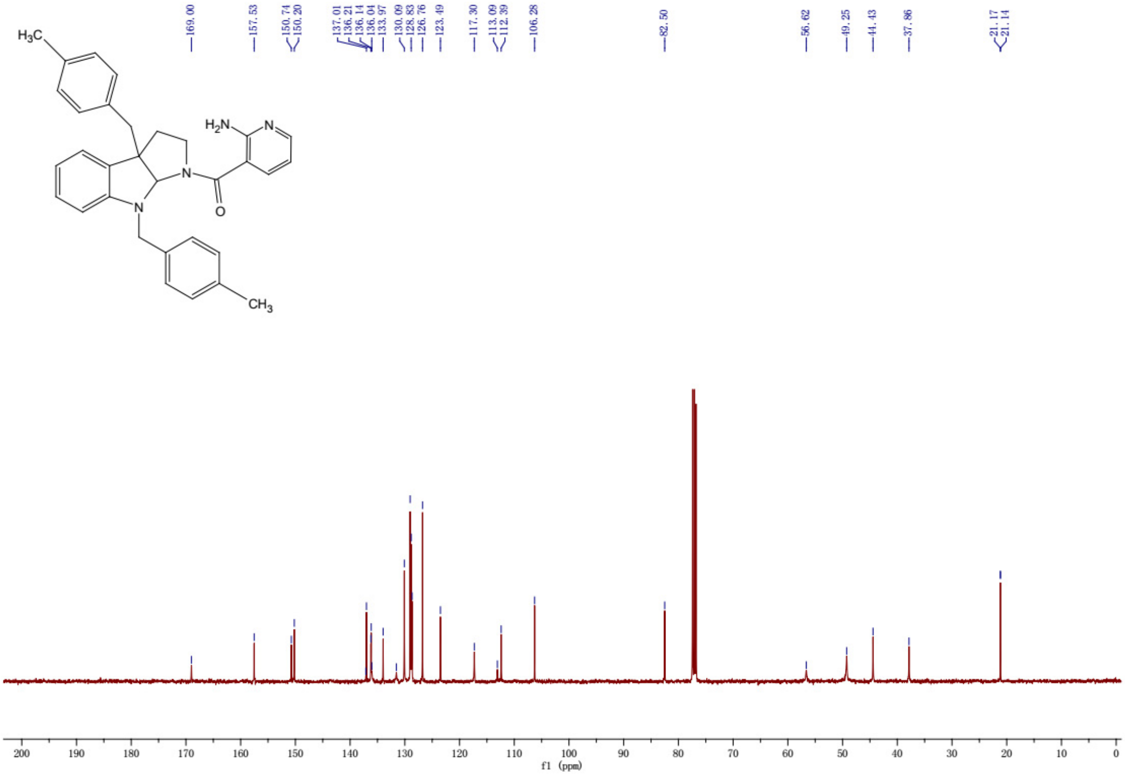

Figure S8 ^{13}C-NMR spectroscopic data of compound **a4**

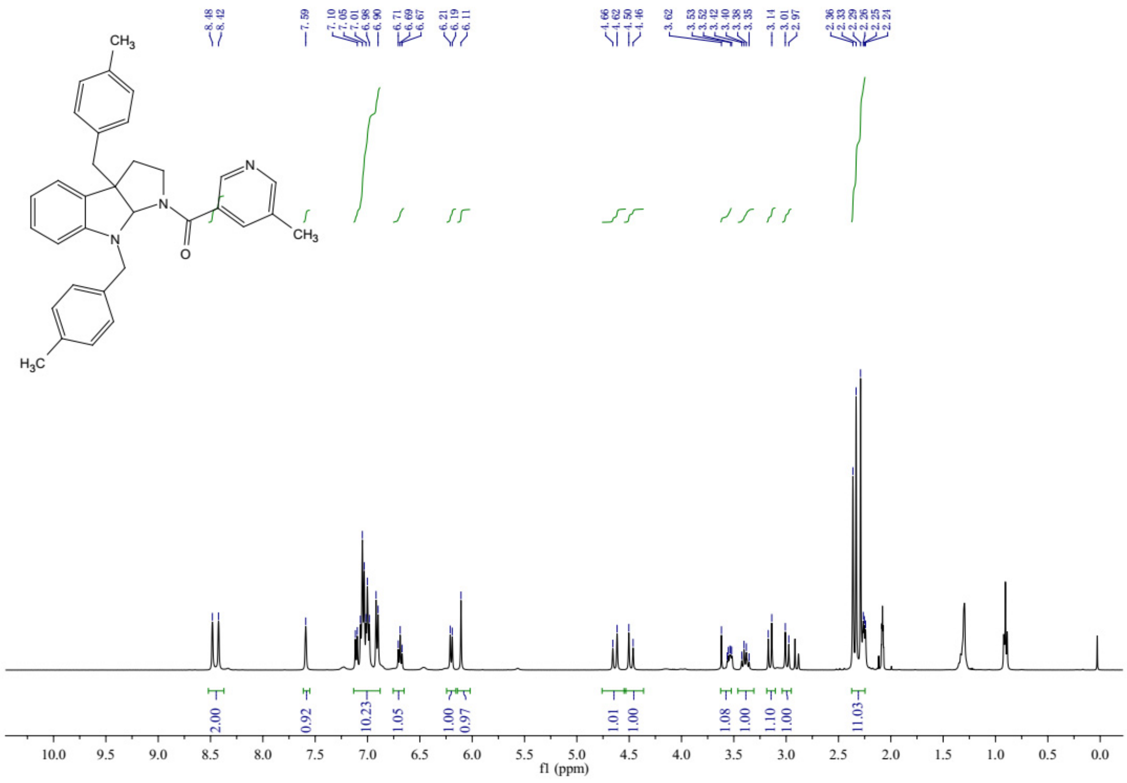

Figure S9 ^{1}H-NMR spectroscopic data of compound **a5**

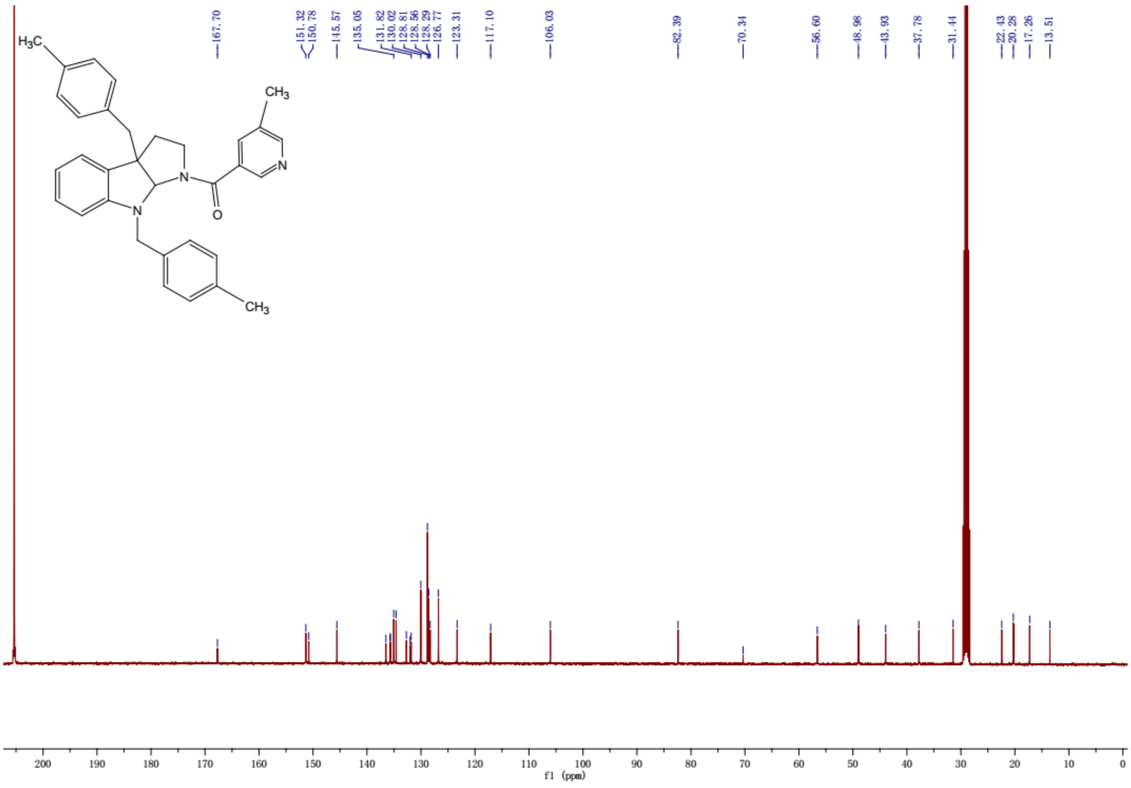

Figure S10 ^{13}C-NMR spectroscopic data of compound **a5**

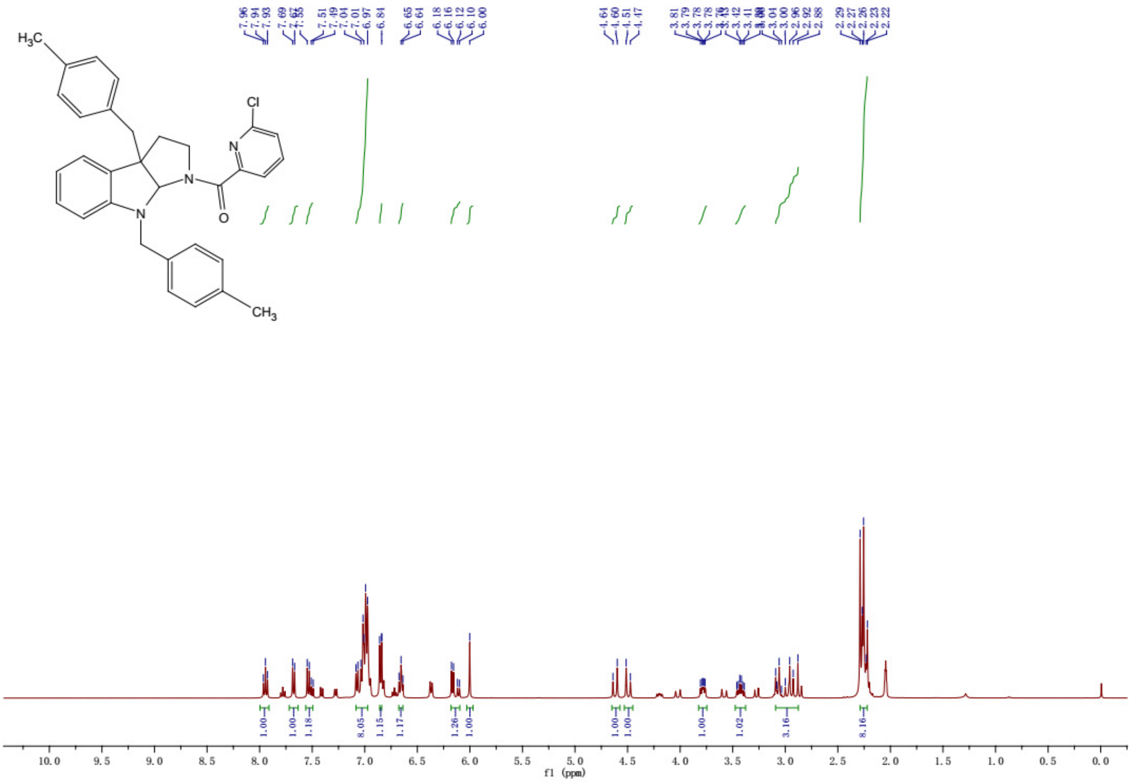

Figure S11 ^{1}H-NMR spectroscopic data of compound **a6**

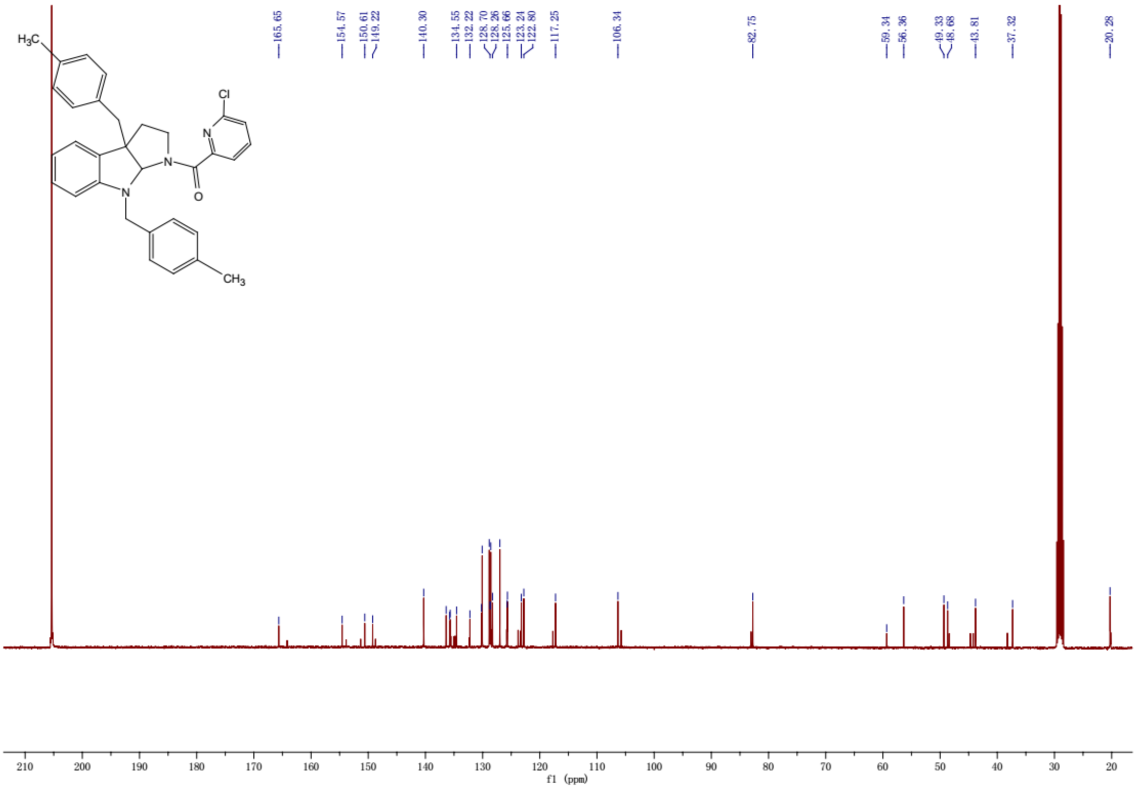

Figure S12 ^{13}C-NMR spectroscopic data of compound **a6**

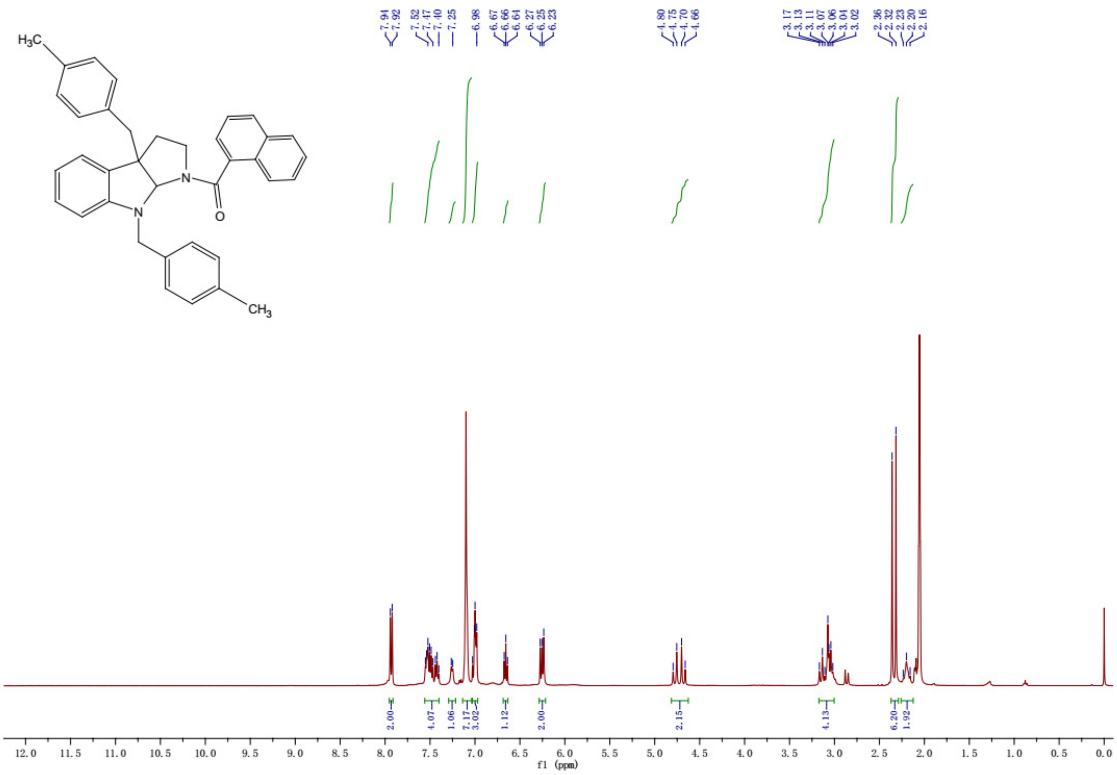

Figure S13 ^{1}H-NMR spectroscopic data of compound **a7**

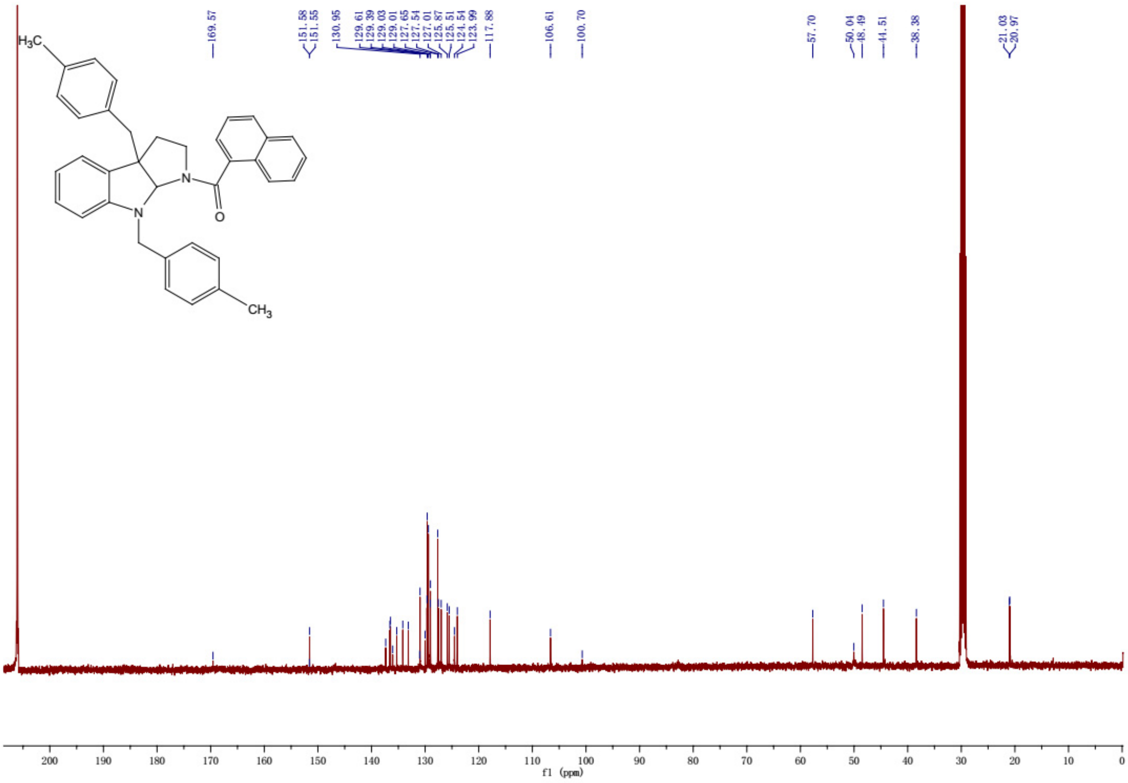

Figure S14 ^{13}C-NMR spectroscopic data of compound **a7**

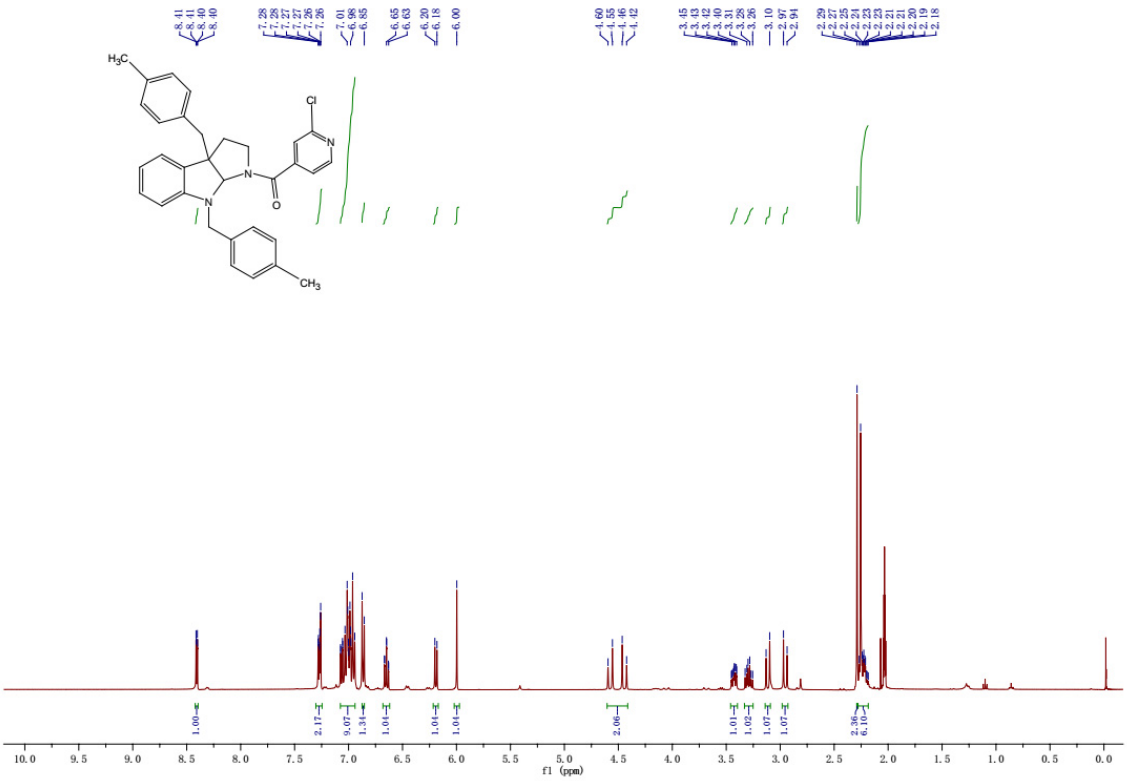

Figure S15 ^{1}H-NMR spectroscopic data of compound **a8**

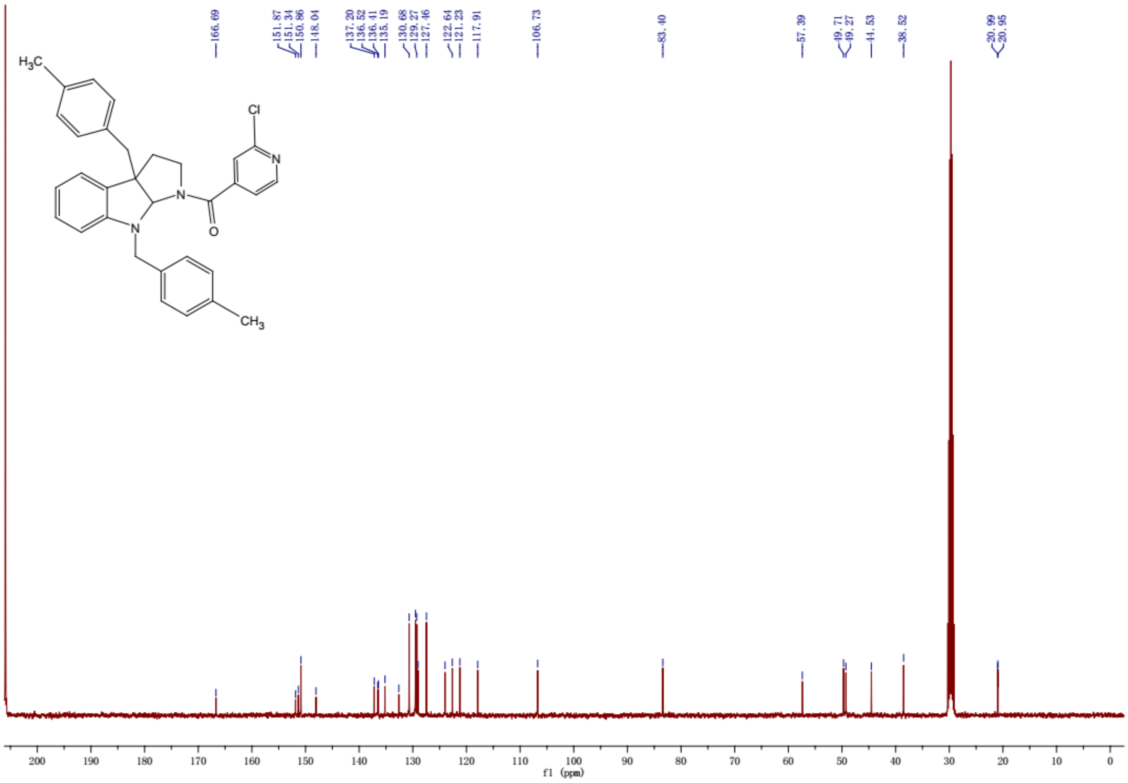

Figure S16 ^{13}C-NMR spectroscopic data of compound **a8**

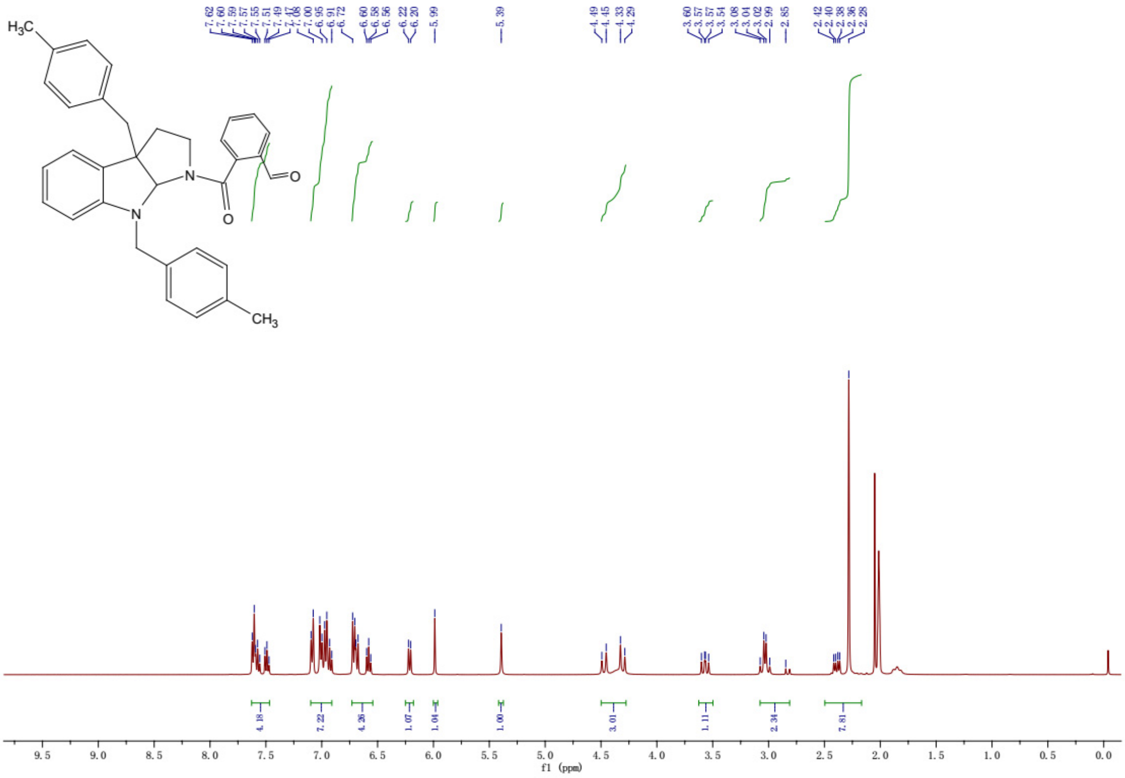

Figure S17 ^{1}H-NMR spectroscopic data of compound **a9**

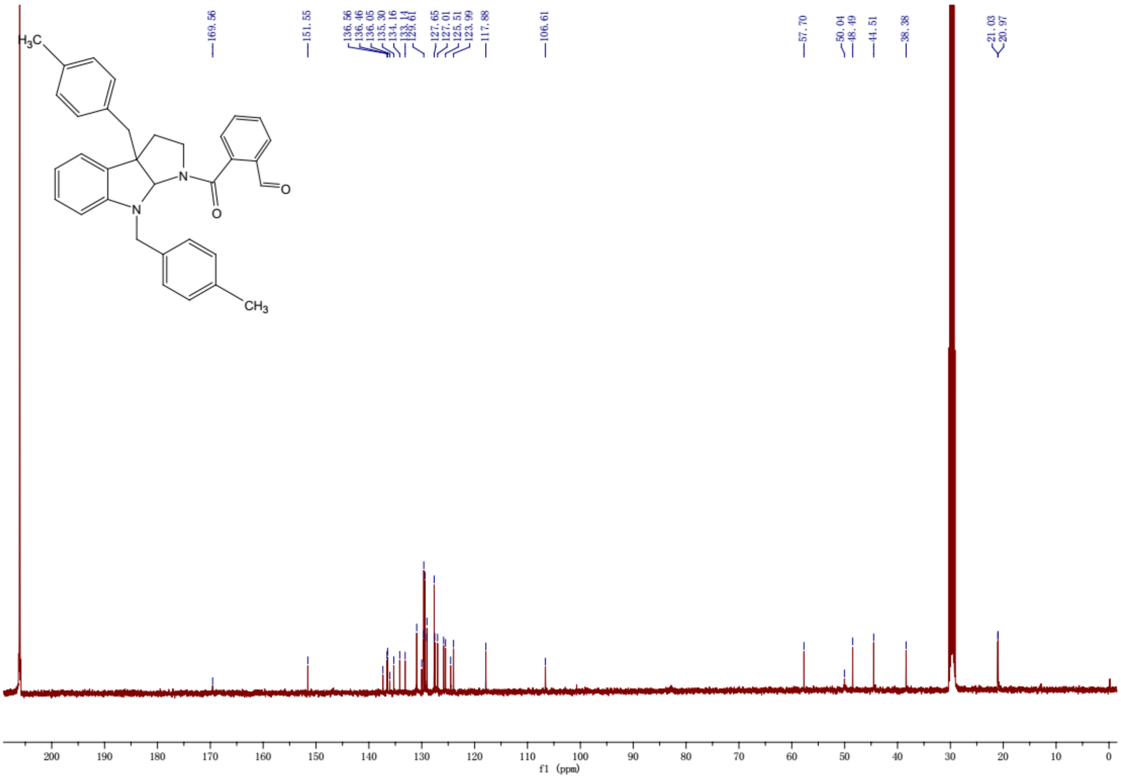

Figure S18 ^{13}C-NMR spectroscopic data of compound **a9**

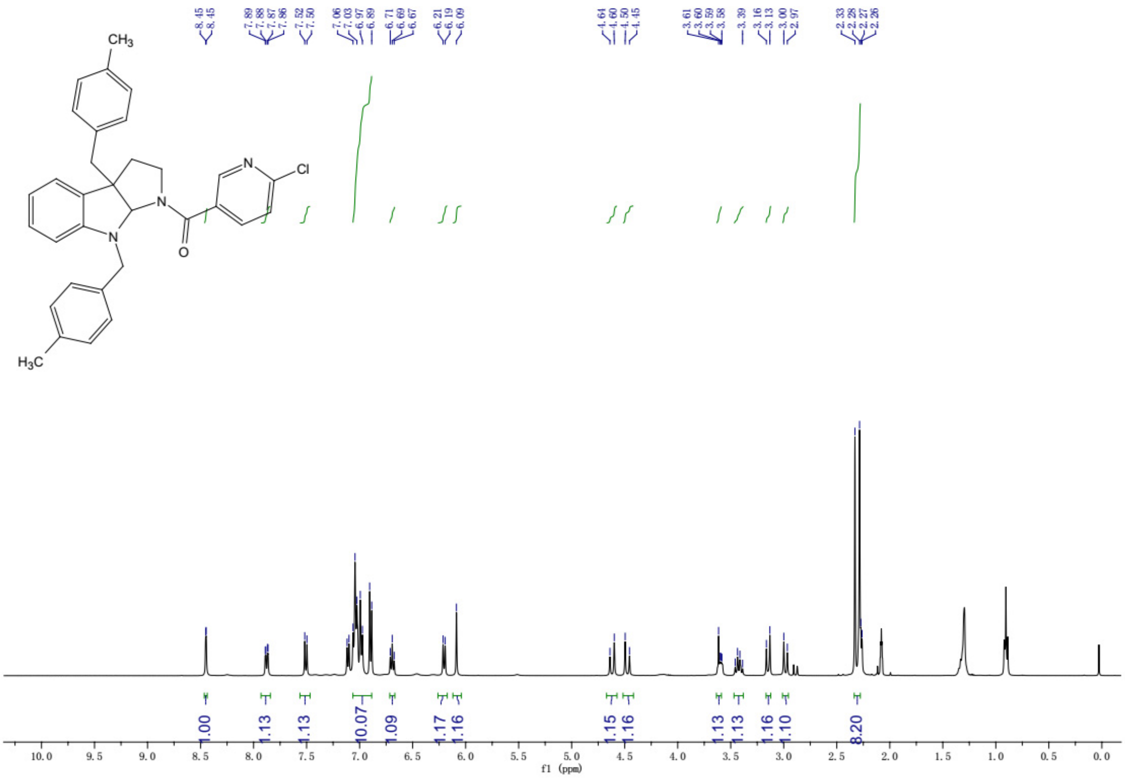

Figure S19 ^{1}H-NMR spectroscopic data of compound **a10**

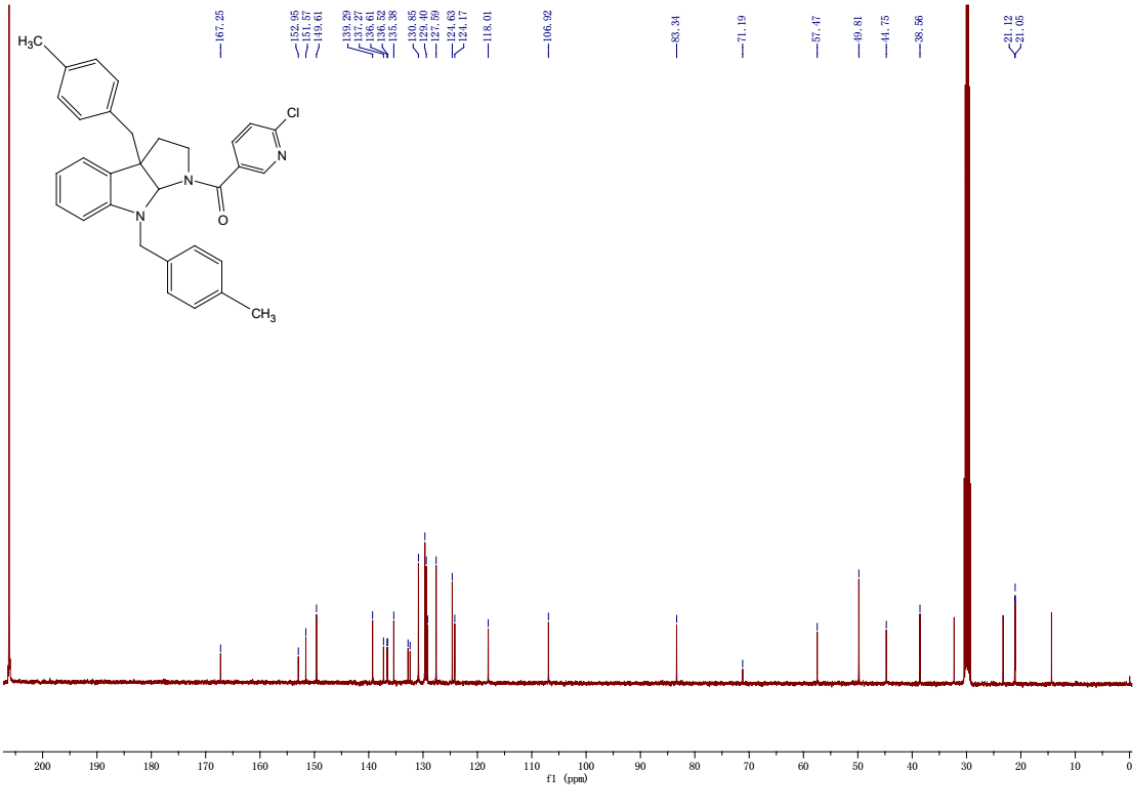

Figure S20 ^{13}C-NMR spectroscopic data of compound **a10**

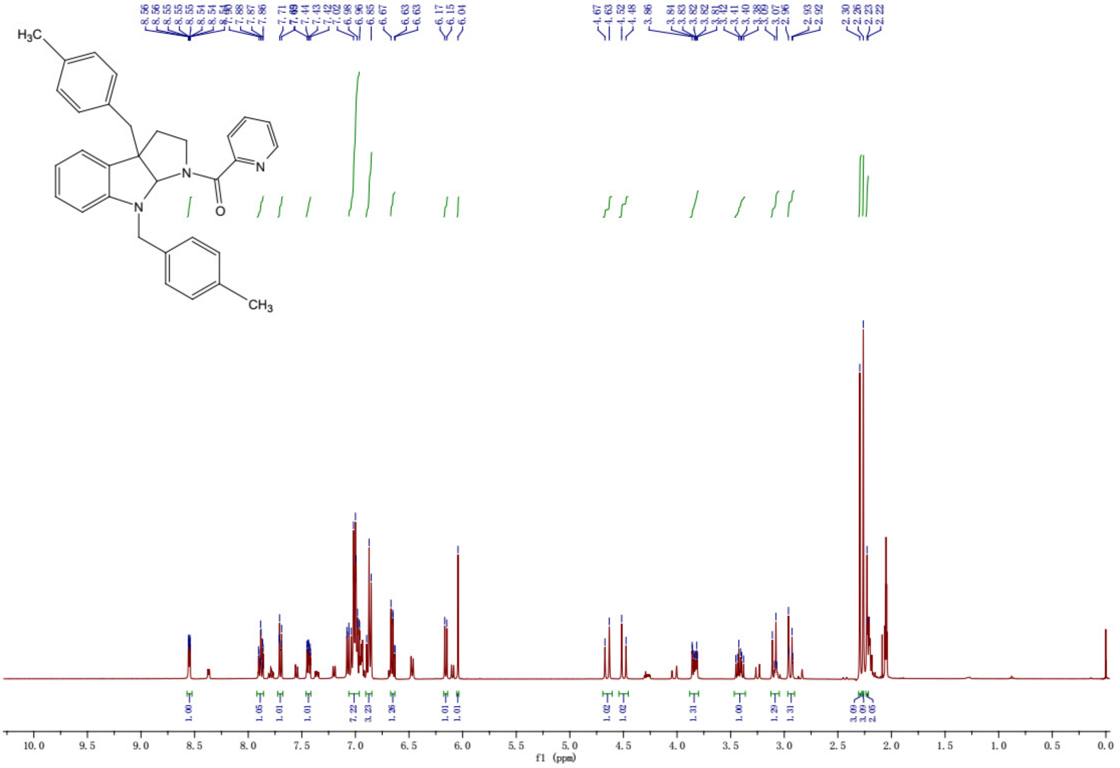

Figure S21 ^{1}H-NMR spectroscopic data of compound **a11**

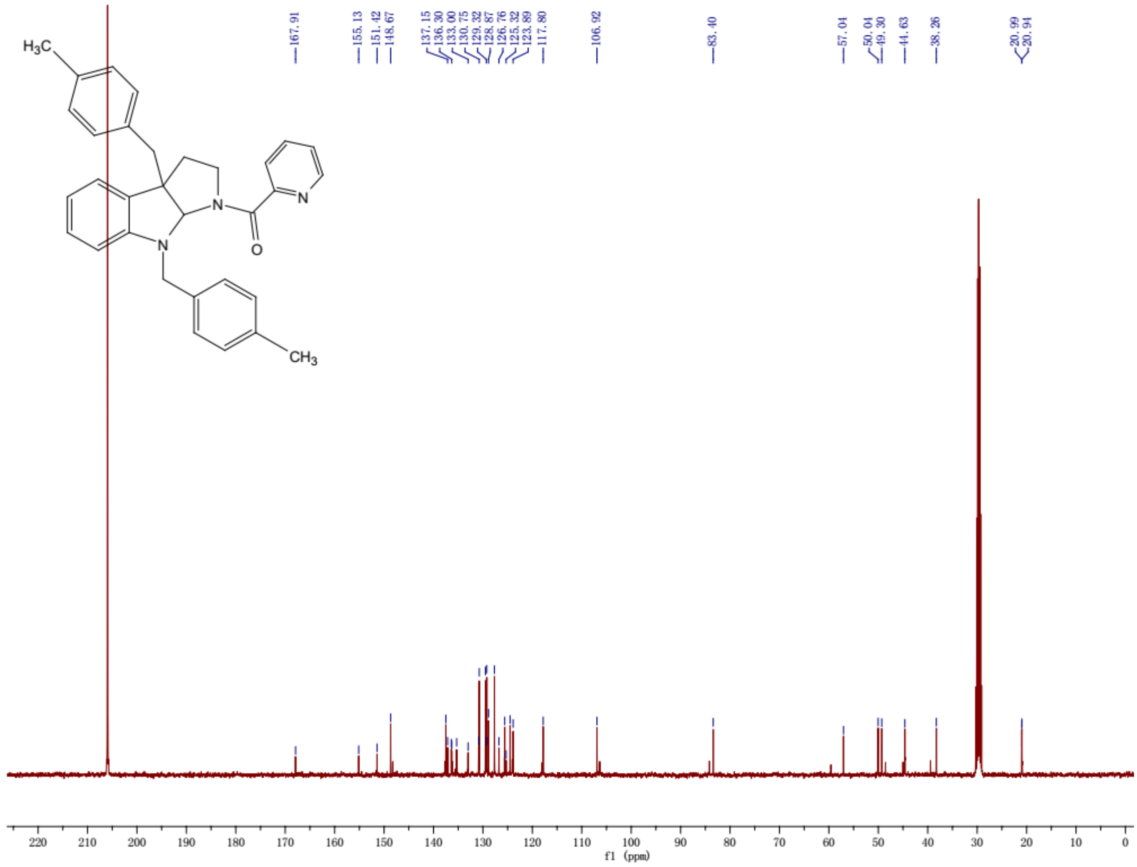

Figure S22 ^{13}C-NMR spectroscopic data of compound **a11**

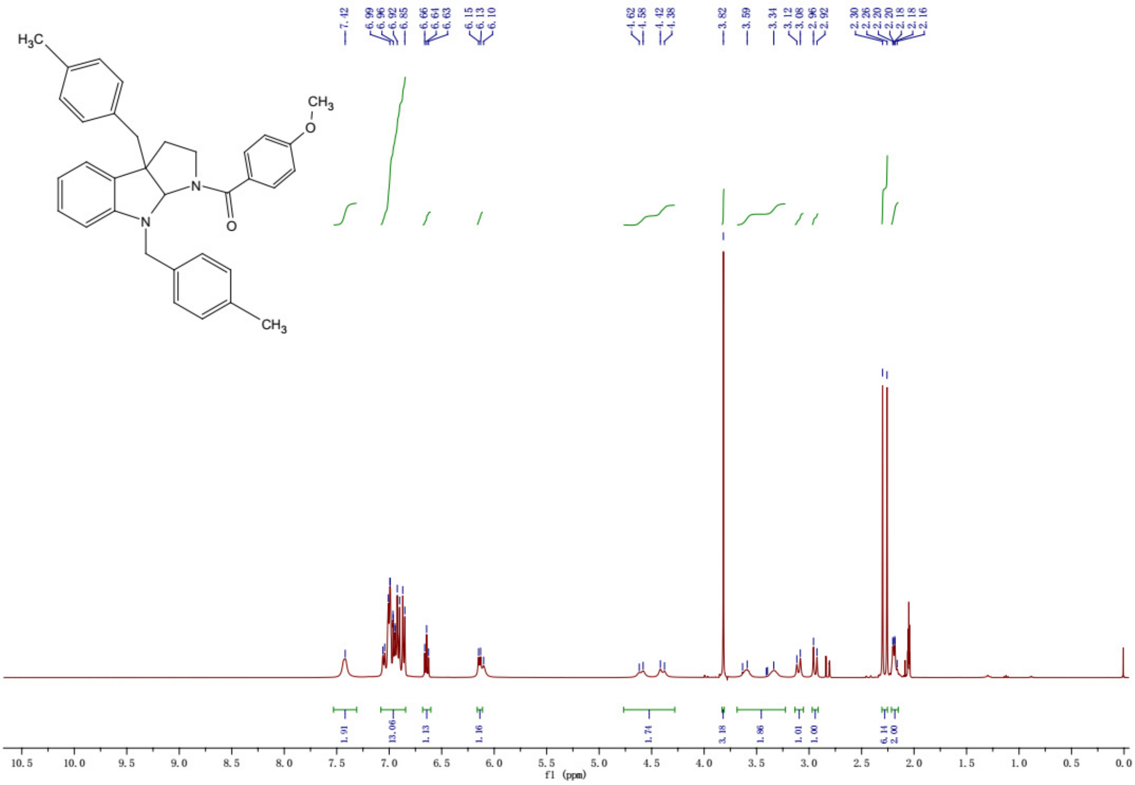

Figure S23 ^{1}H-NMR spectroscopic data of compound **a12**

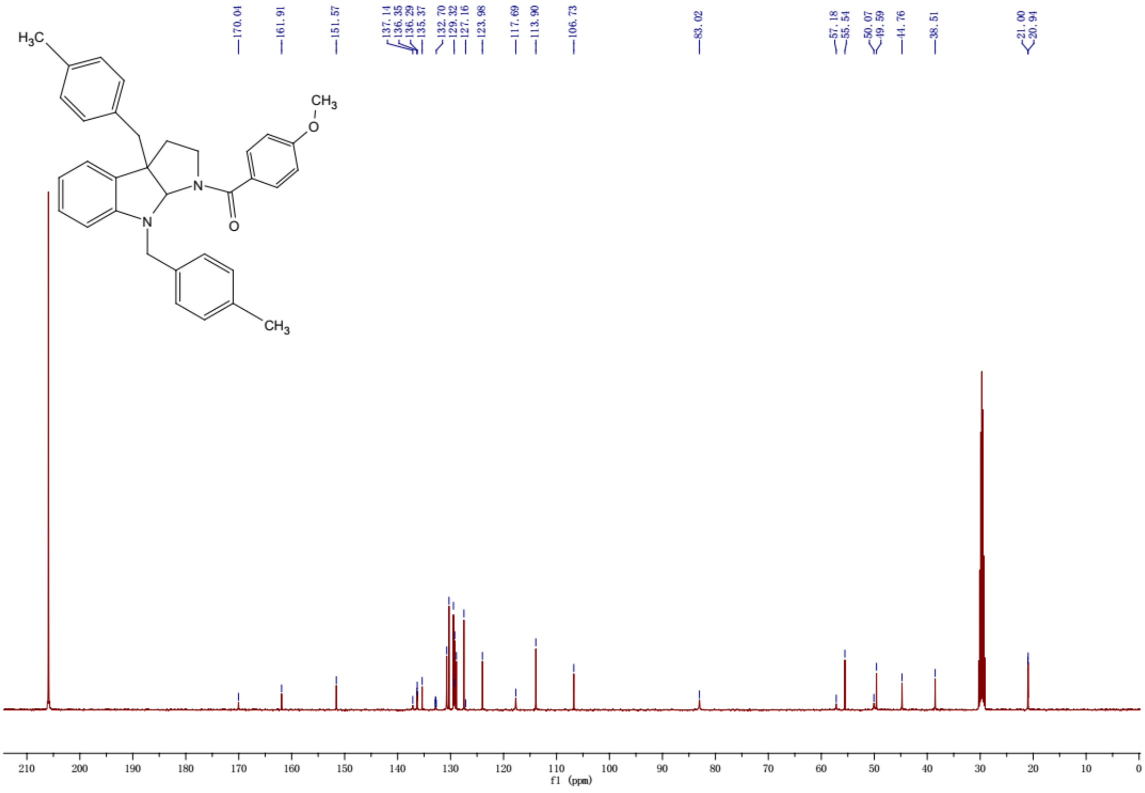

Figure S24 ^{13}C-NMR spectroscopic data of compound **a12**

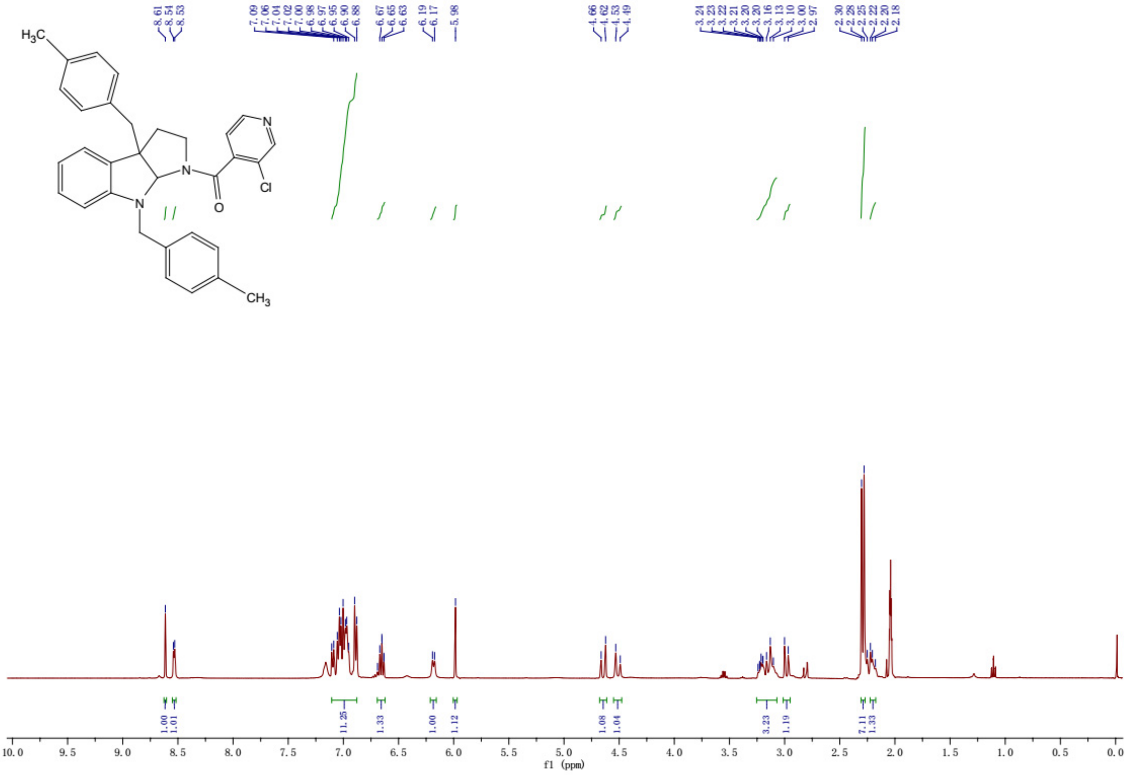

Figure S25 ^{1}H-NMR spectroscopic data of compound **a13**

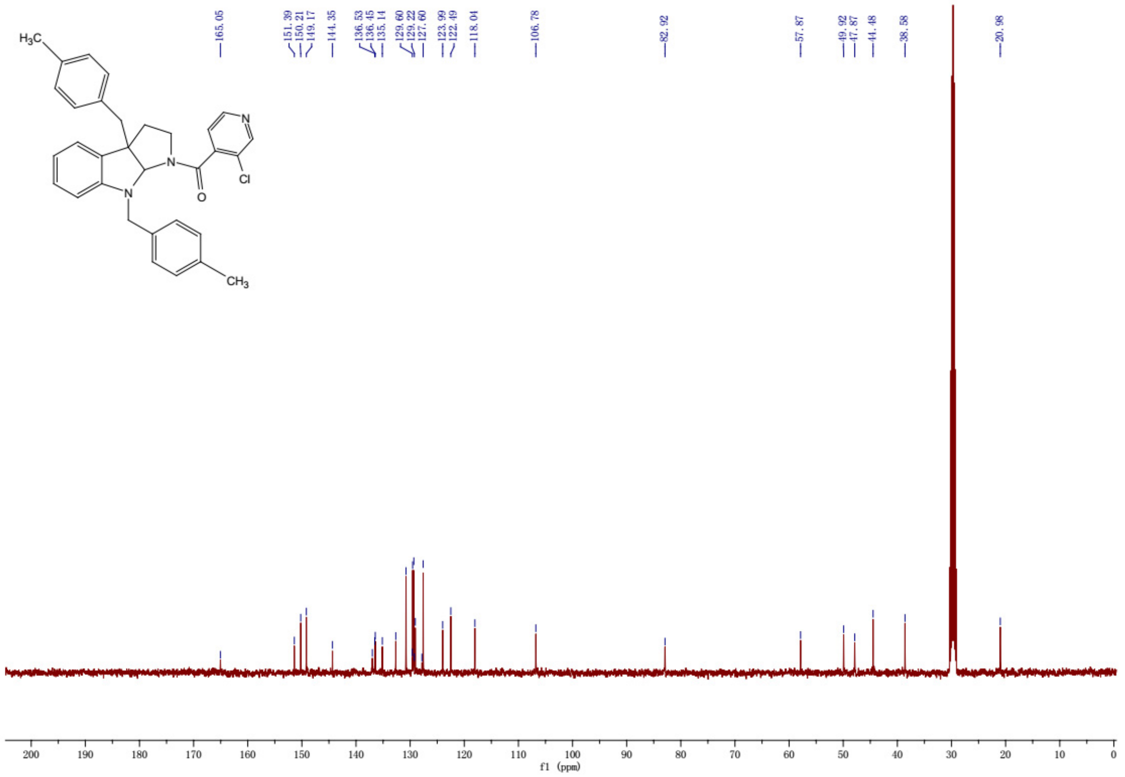

Figure S26 ^{13}C-NMR spectroscopic data of compound **a13**

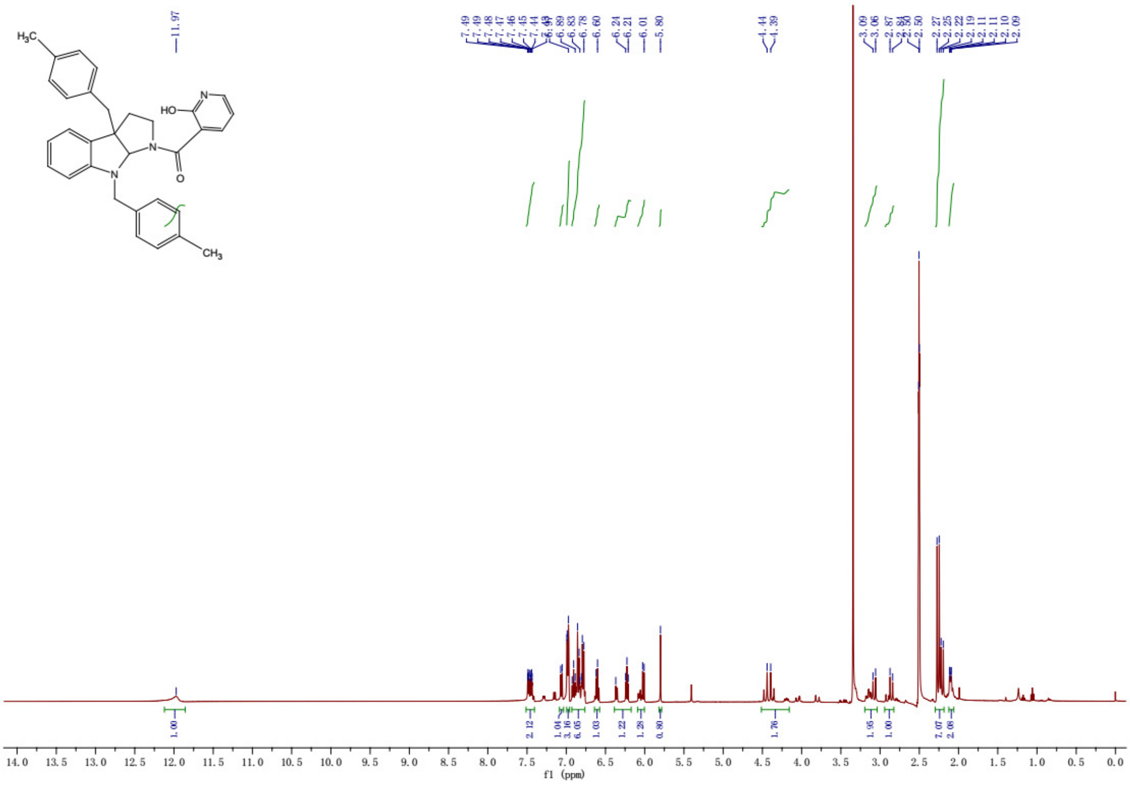

Figure S27 ^{1}H-NMR spectroscopic data of compound **a14**

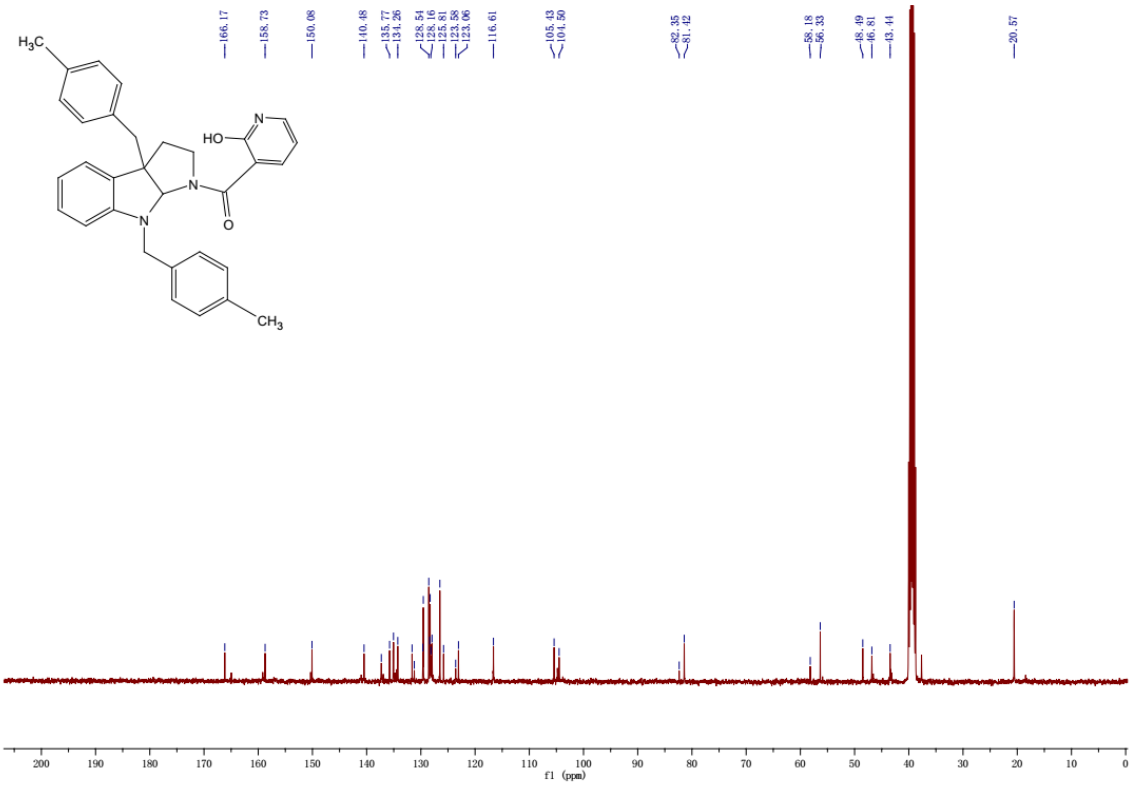

Figure S28 ^{13}C-NMR spectroscopic data of compound **a14**

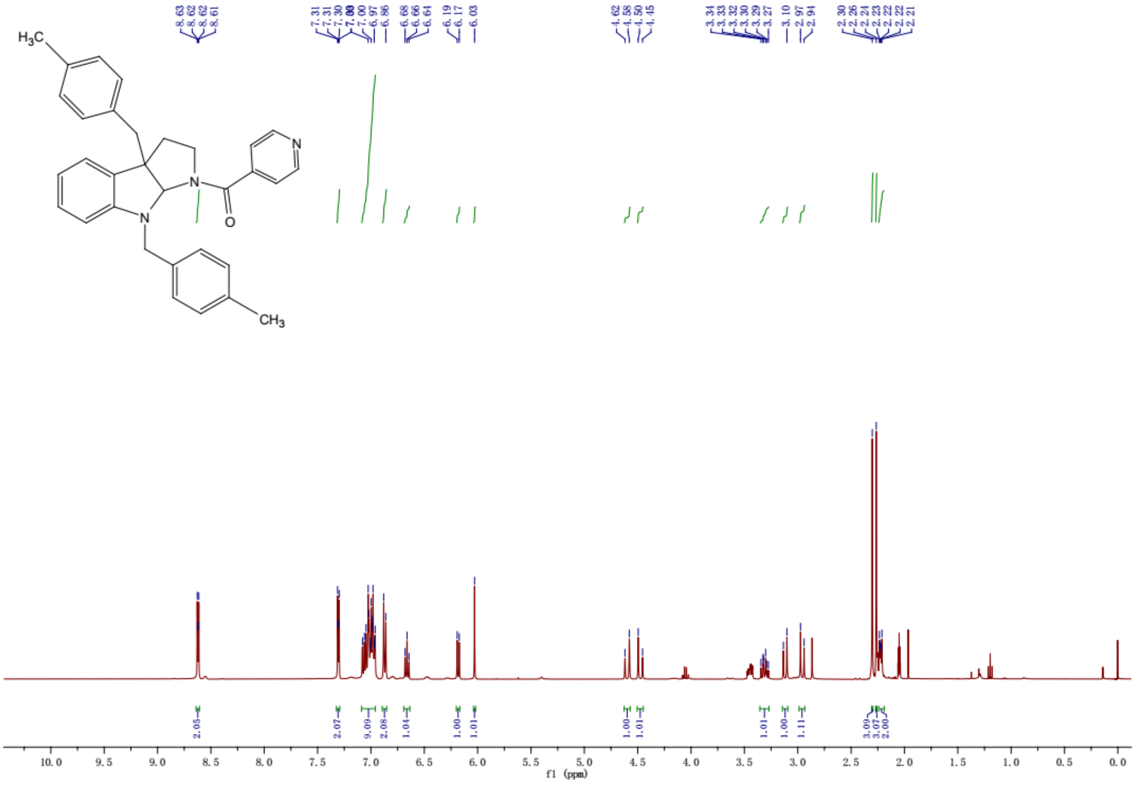

Figure S29 ^{1}H-NMR spectroscopic data of compound **a15**

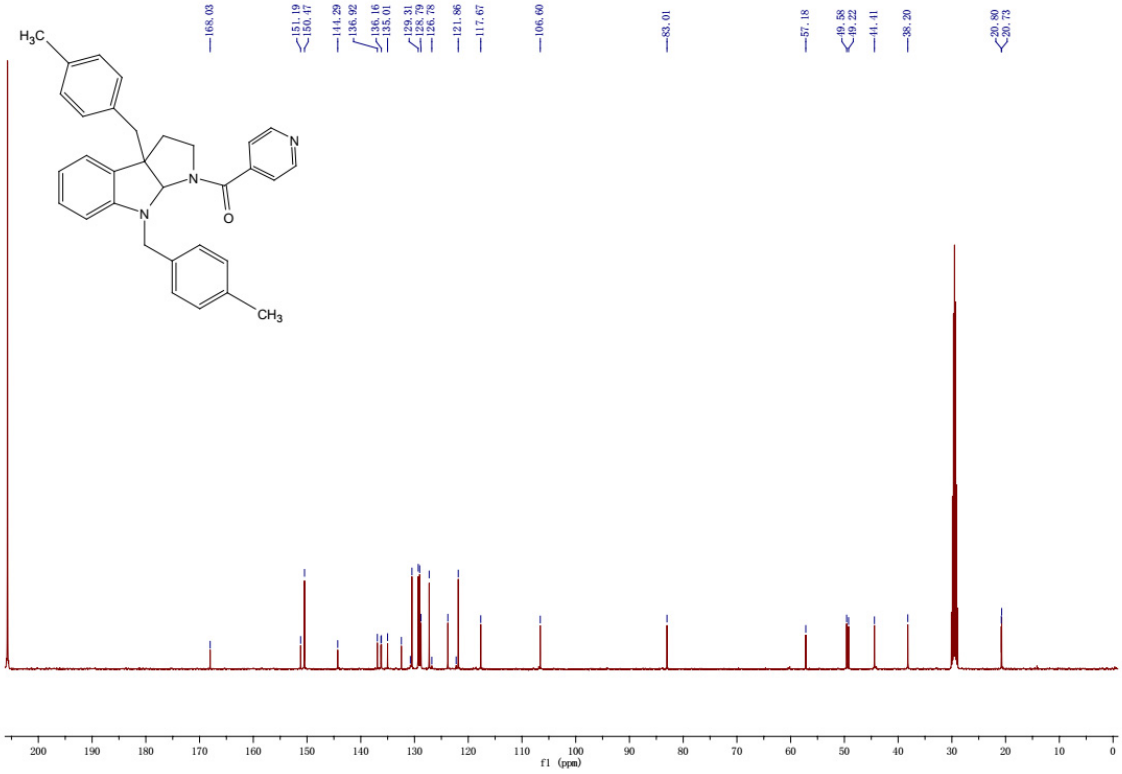

Figure S30 ^{13}C-NMR spectroscopic data of compound **a15**

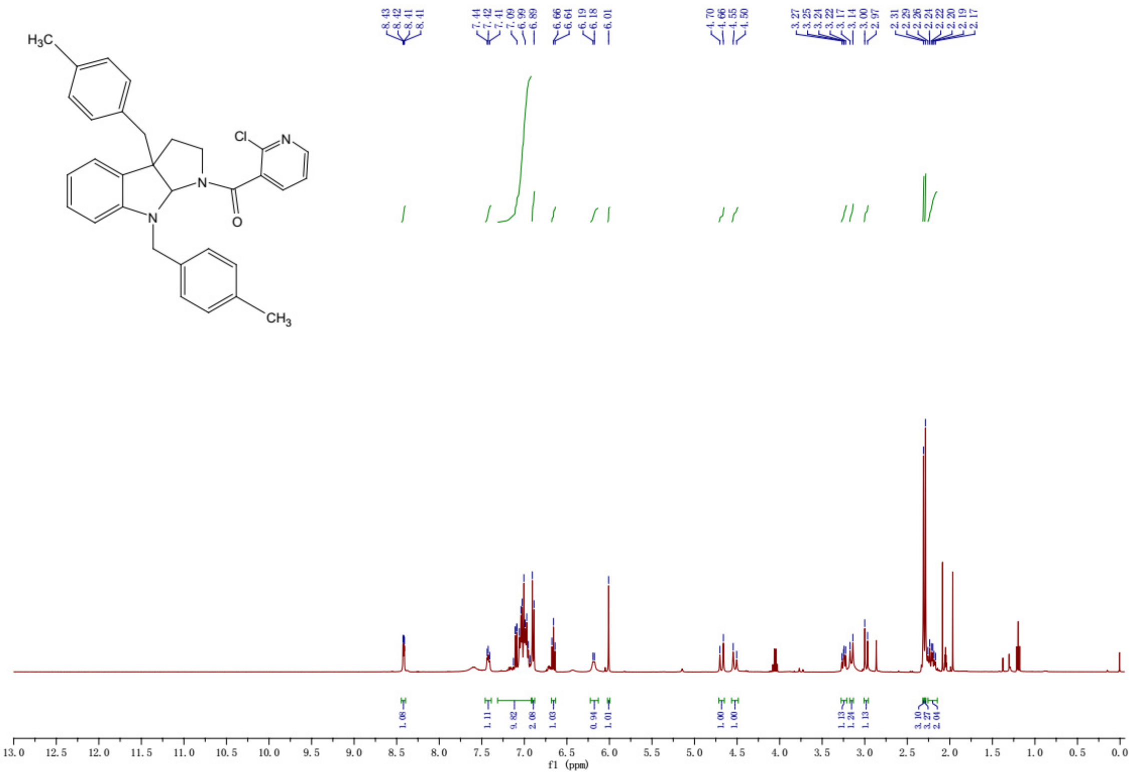

Figure S31 ^{1}H-NMR spectroscopic data of compound **a16**

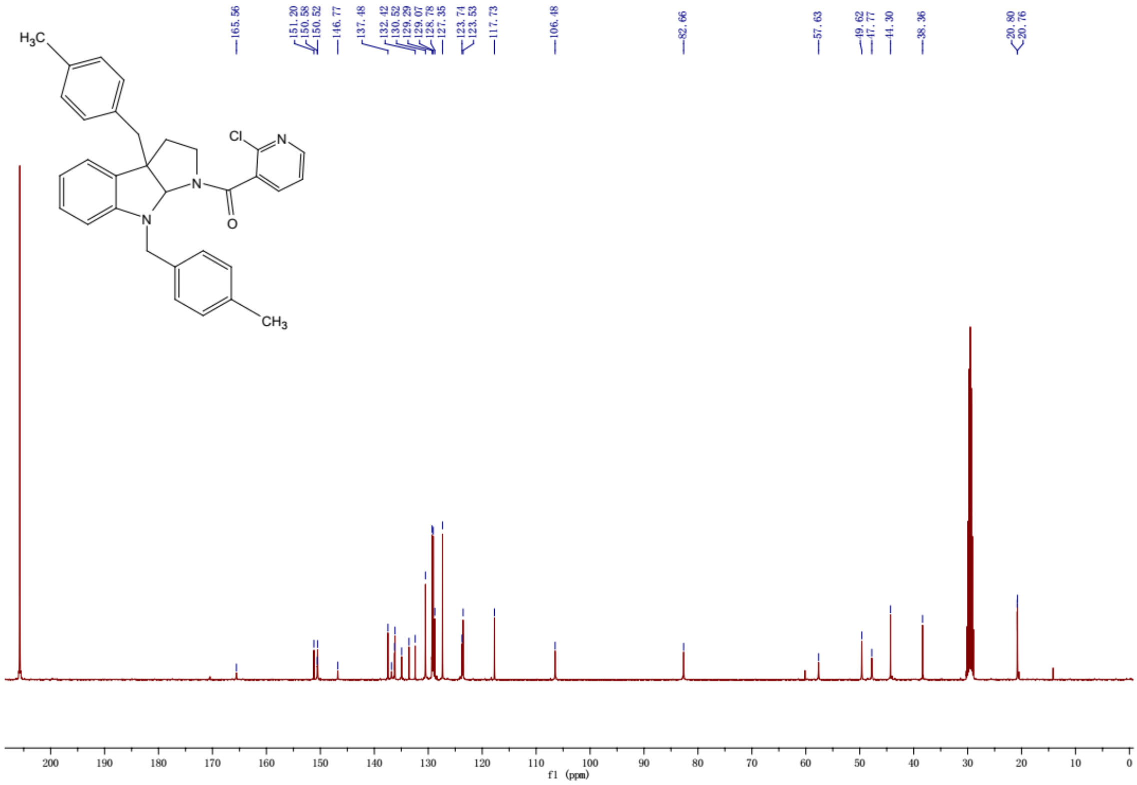

Figure S32 ^{13}C-NMR spectroscopic data of compound **a16**

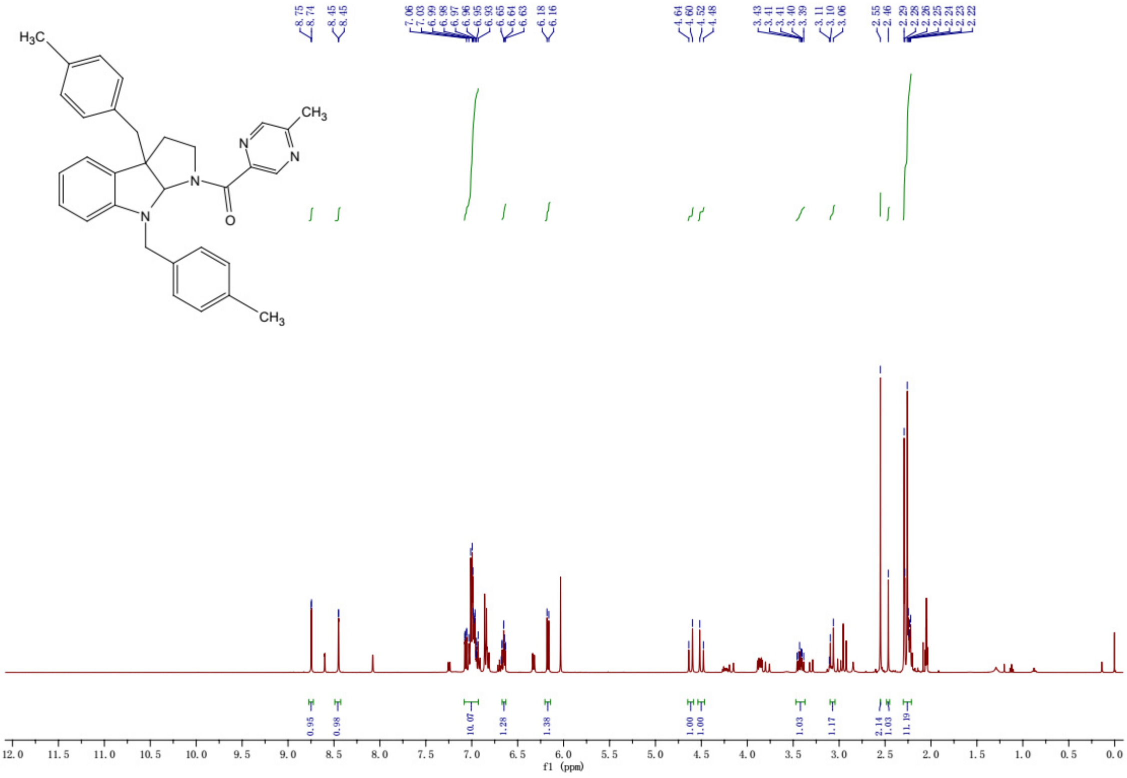

Figure S33 ^{1}H-NMR spectroscopic data of compound **a17**

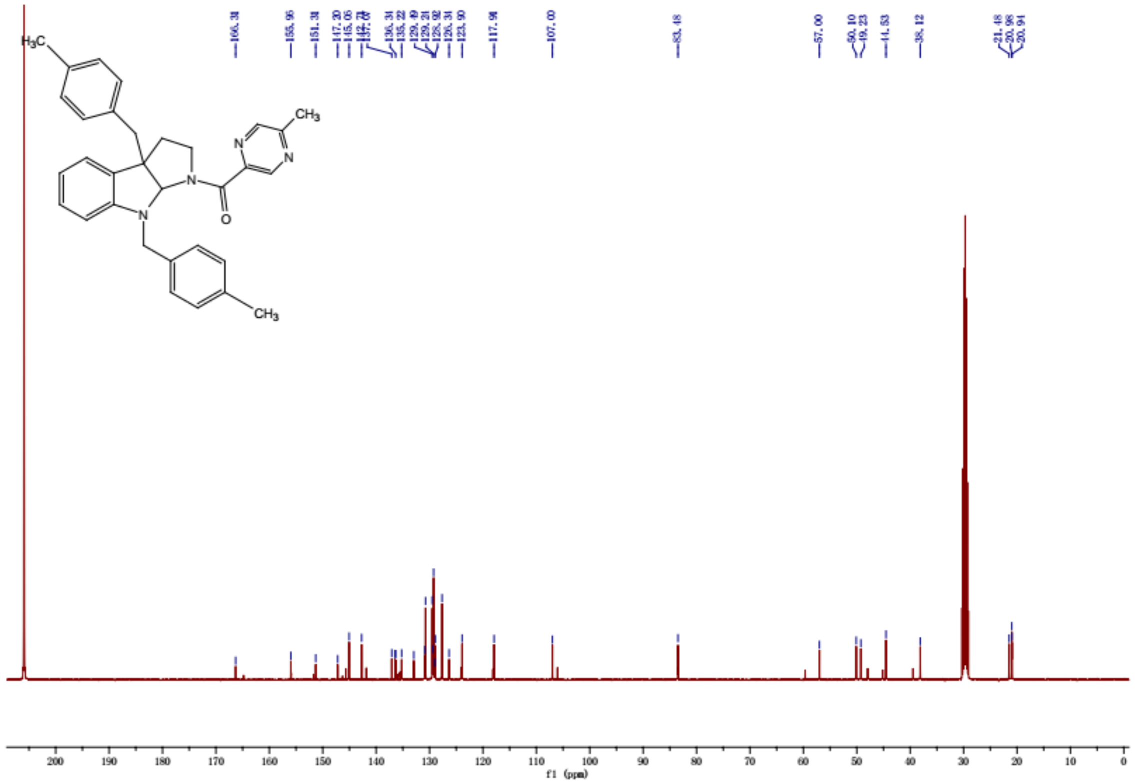

Figure S34 ^{13}C-NMR spectroscopic data of compound **a17**

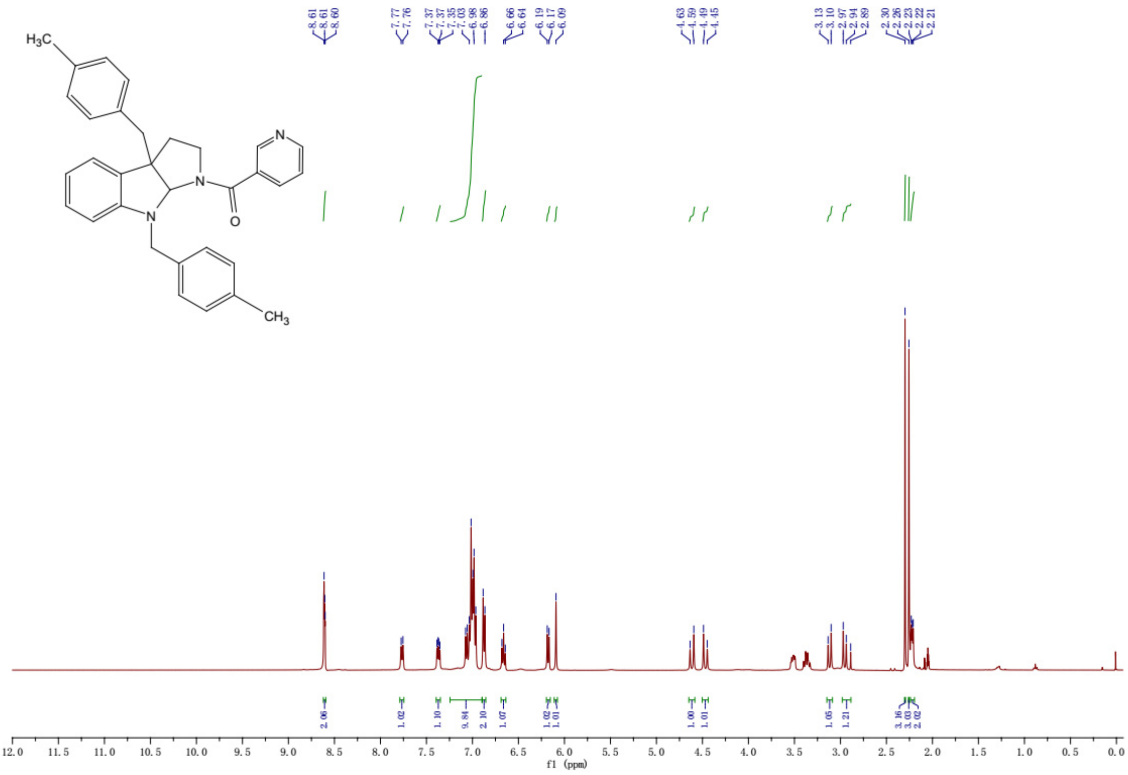

Figure S35 ^{1}H-NMR spectroscopic data of compound **a18**

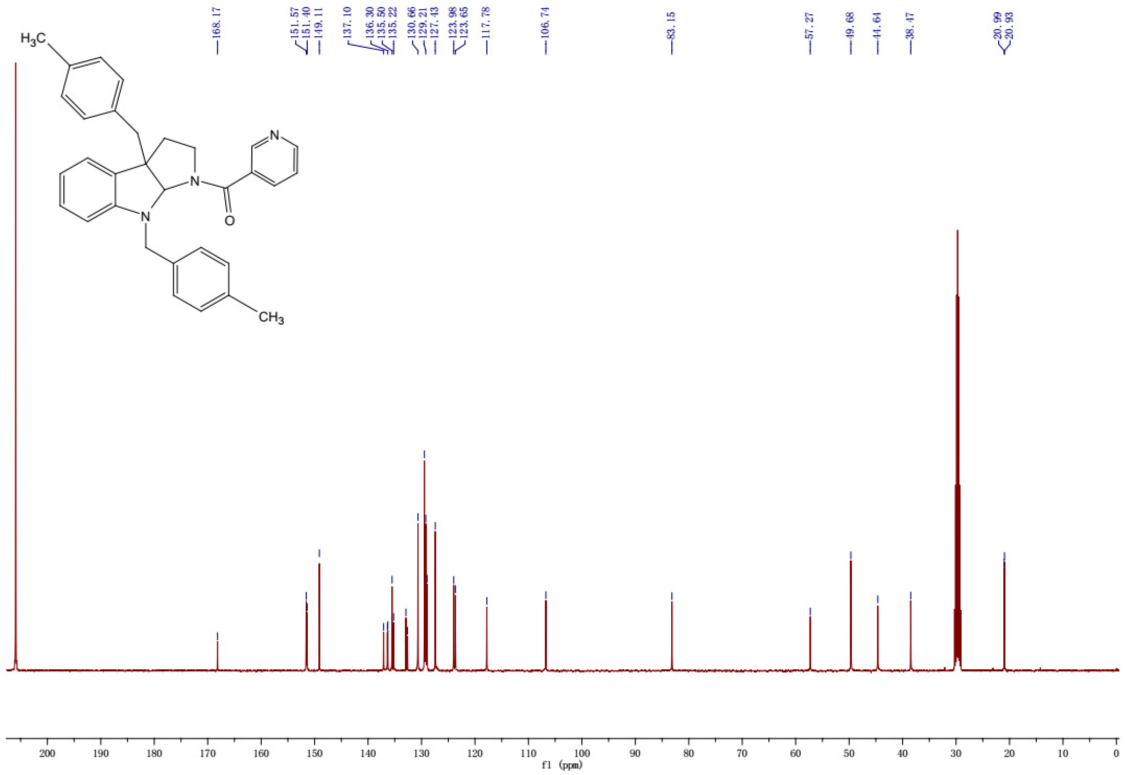

Figure S36 ^{13}C-NMR spectroscopic data of compound **a18**

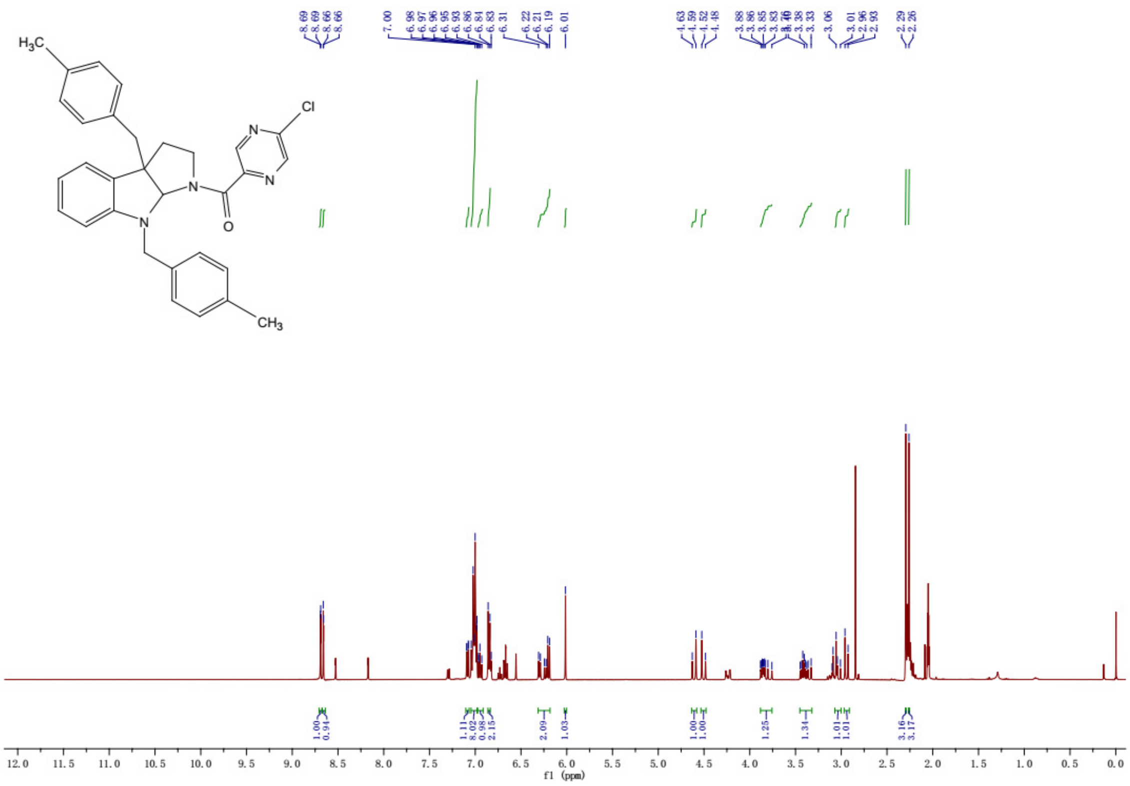

Figure S37 ^{1}H-NMR spectroscopic data of compound **a19**

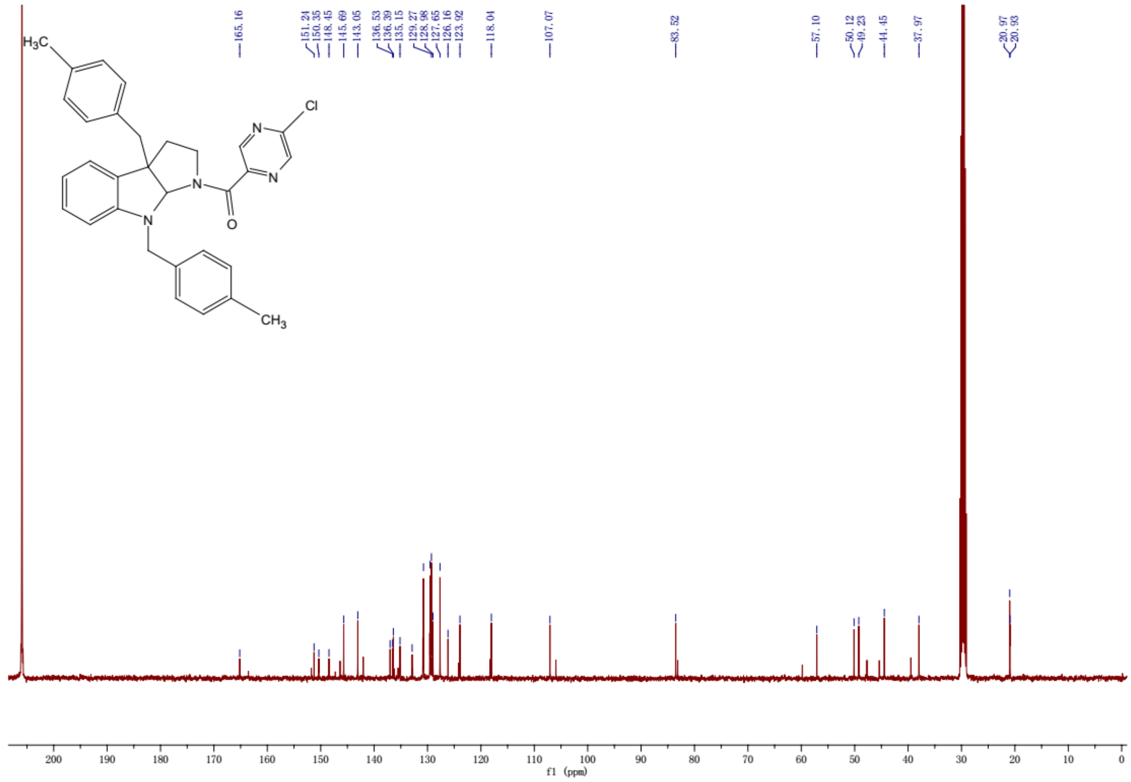

Figure S38 ^{13}C-NMR spectroscopic data of compound **a19**

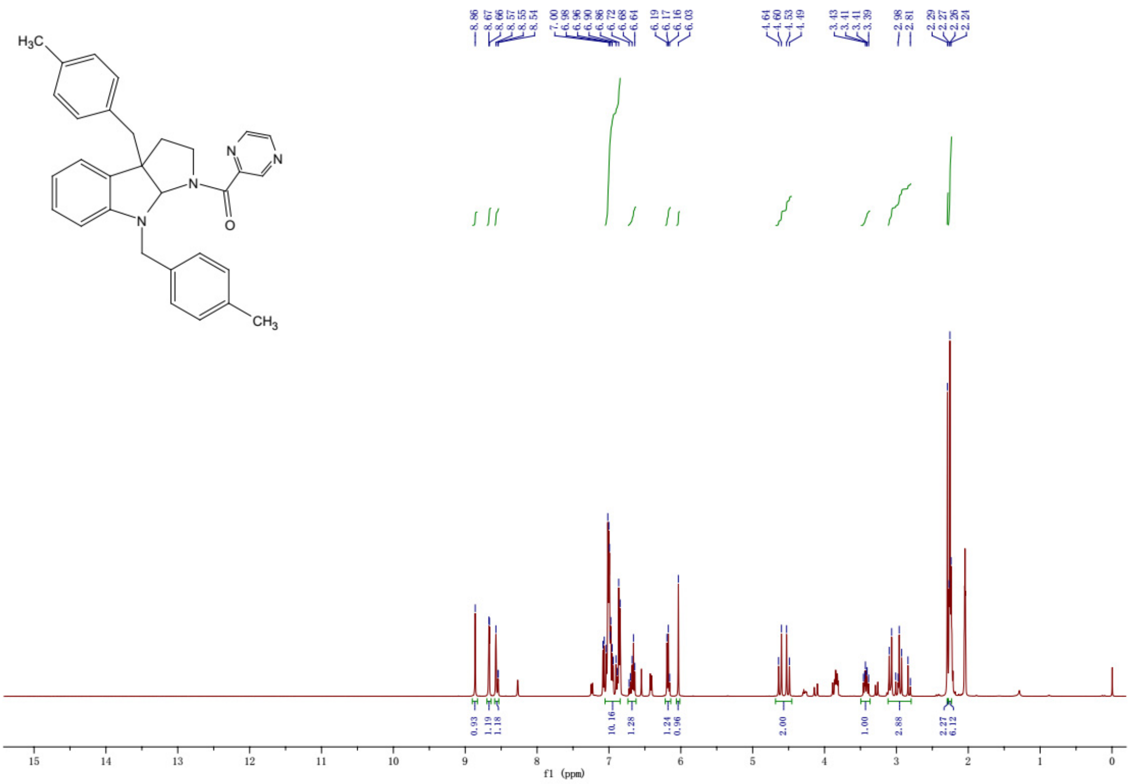

Figure S39 ^{1}H-NMR spectroscopic data of compound **a20**

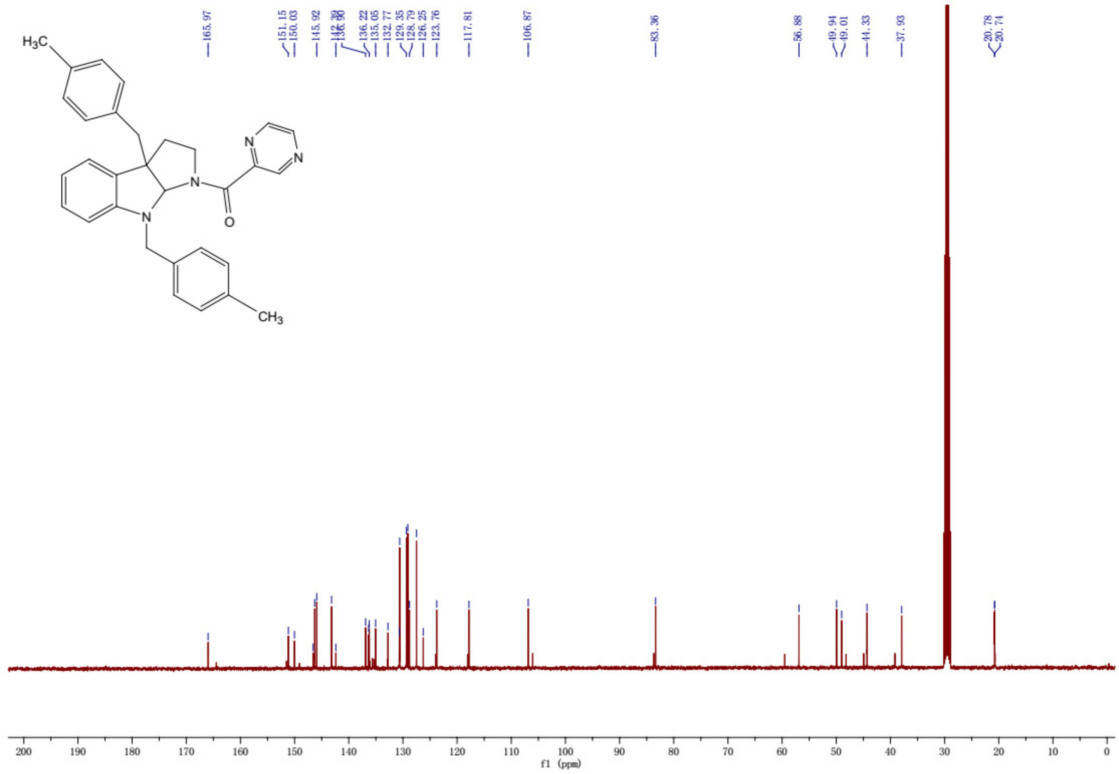

Figure S40 ^{13}C-NMR spectroscopic data of compound **a20**

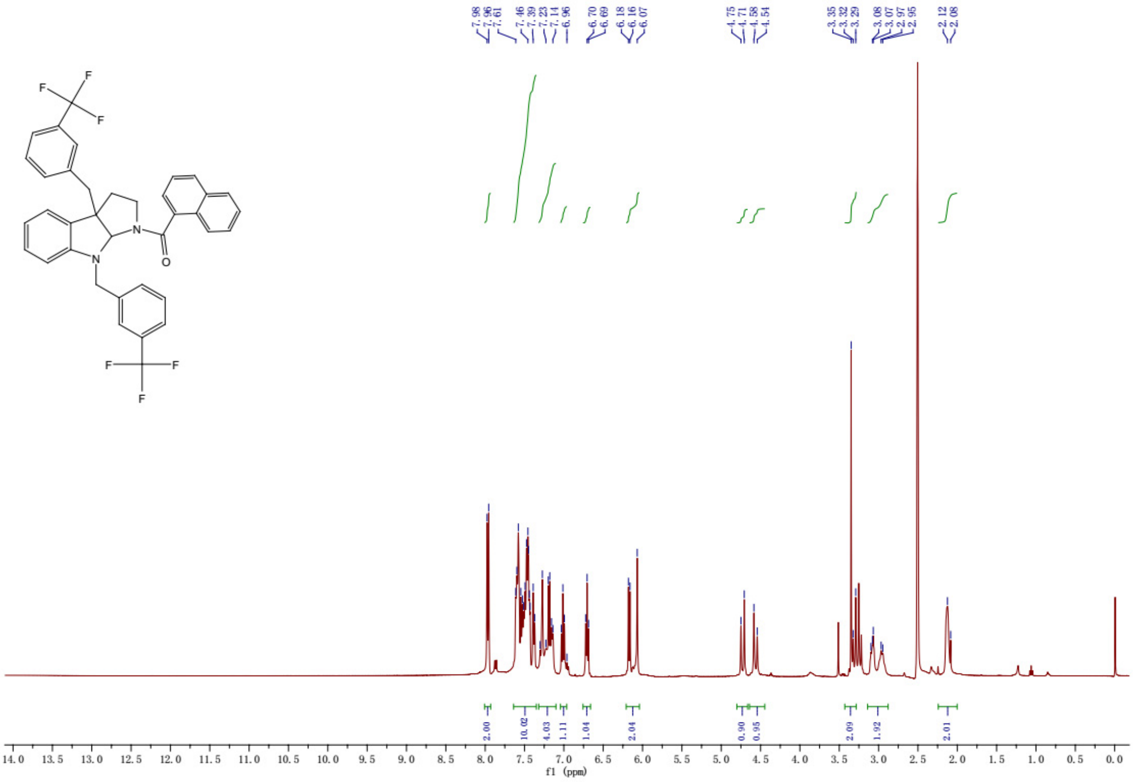

Figure S41 ^{1}H-NMR spectroscopic data of compound **b1**

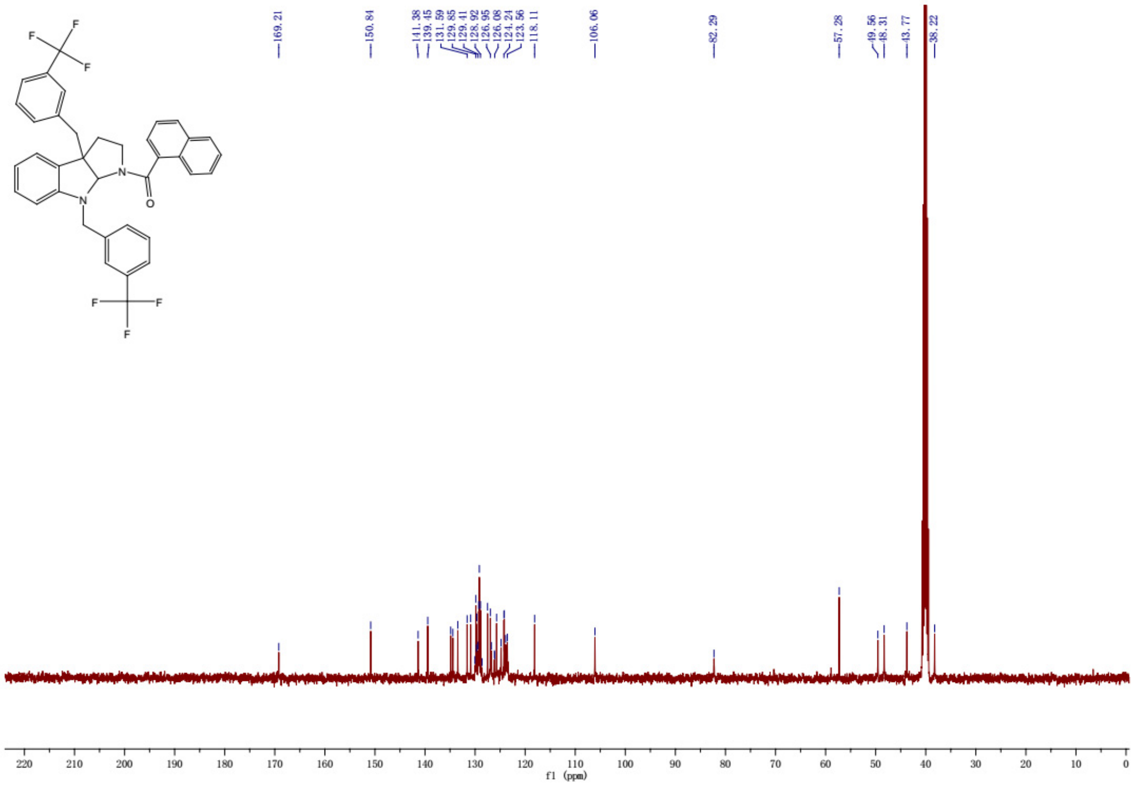

Figure S42 ^{13}C-NMR spectroscopic data of compound **b1**

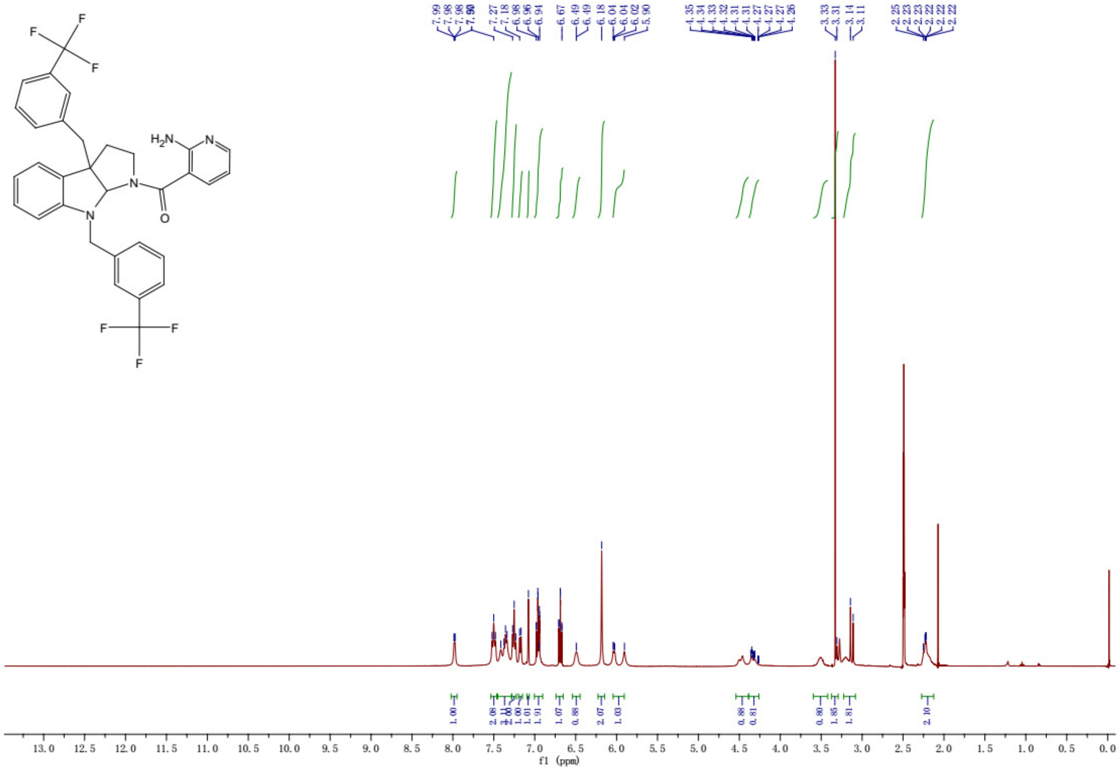

Figure S43 ^{1}H-NMR spectroscopic data of compound **b2**

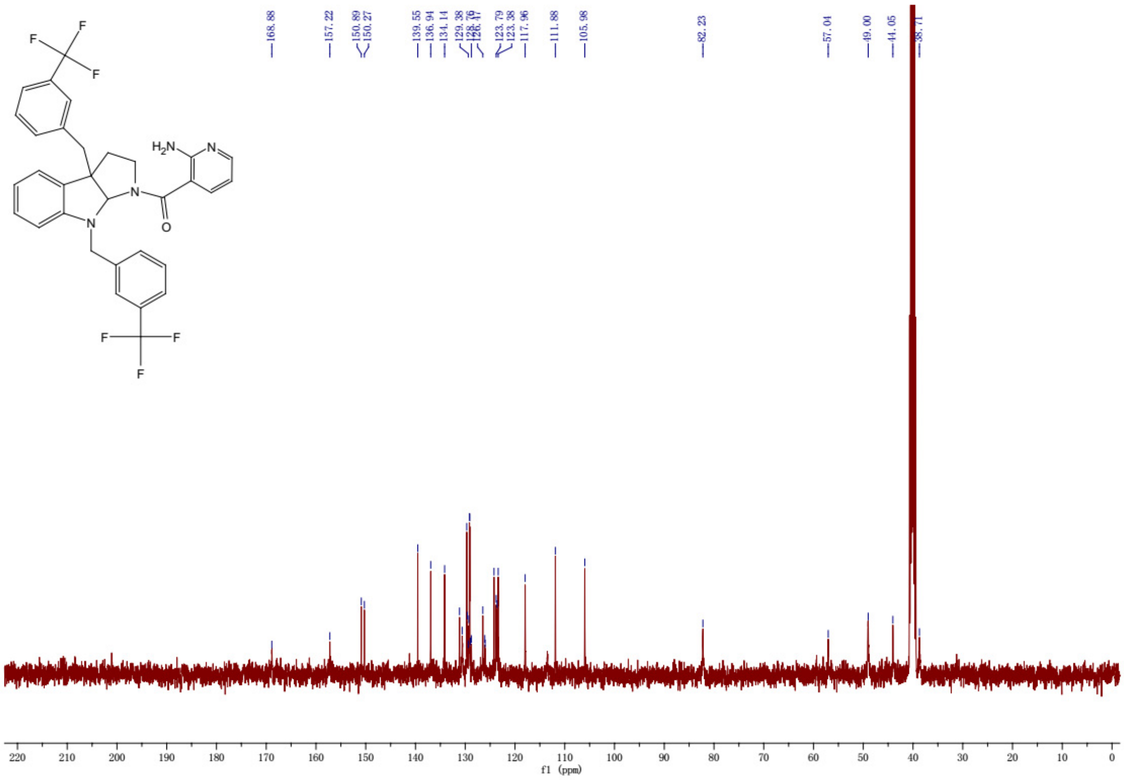

Figure S44 ^{13}C-NMR spectroscopic data of compound **b2**

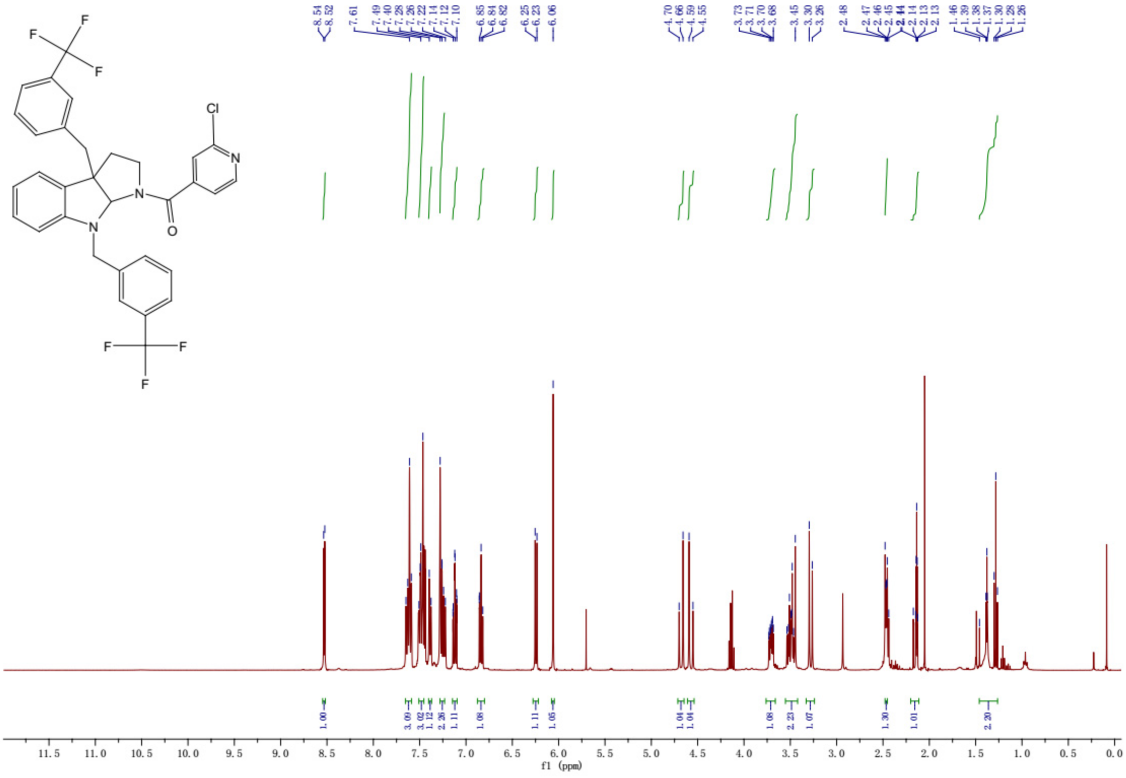

Figure S45 ^{1}H-NMR spectroscopic data of compound **b3**

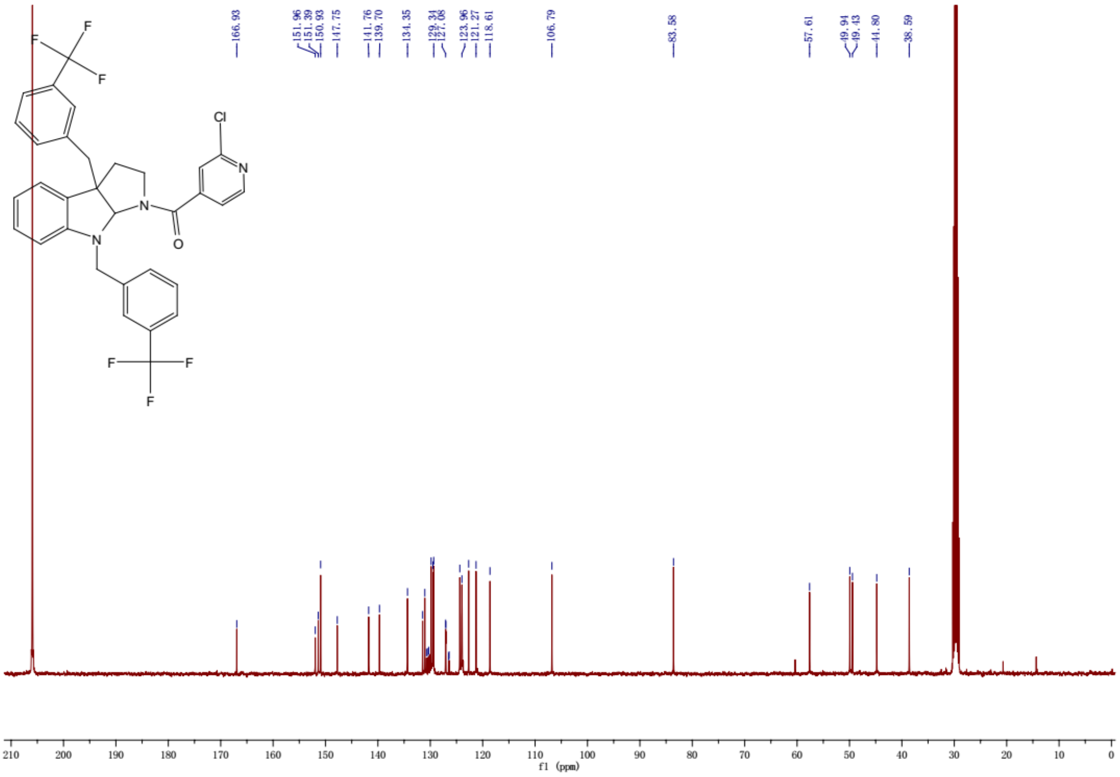

Figure S46 ^{13}C-NMR spectroscopic data of compound **b3**

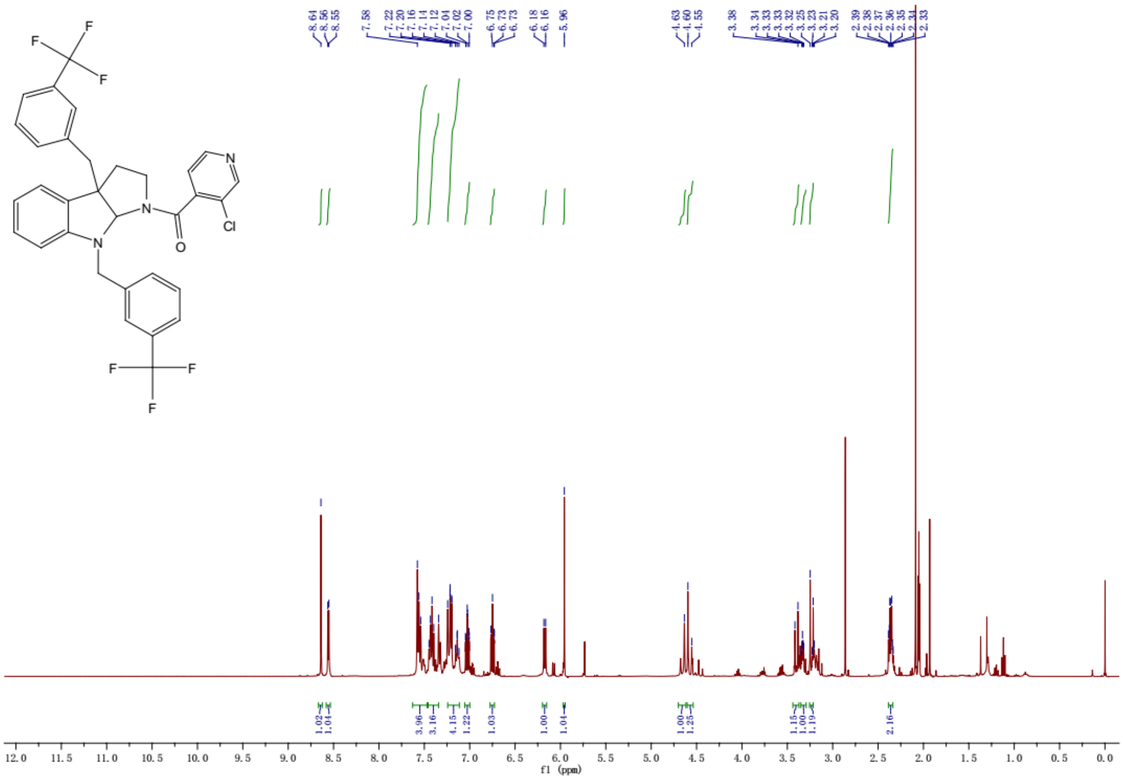

Figure S47 ^{1}H-NMR spectroscopic data of compound **b4**

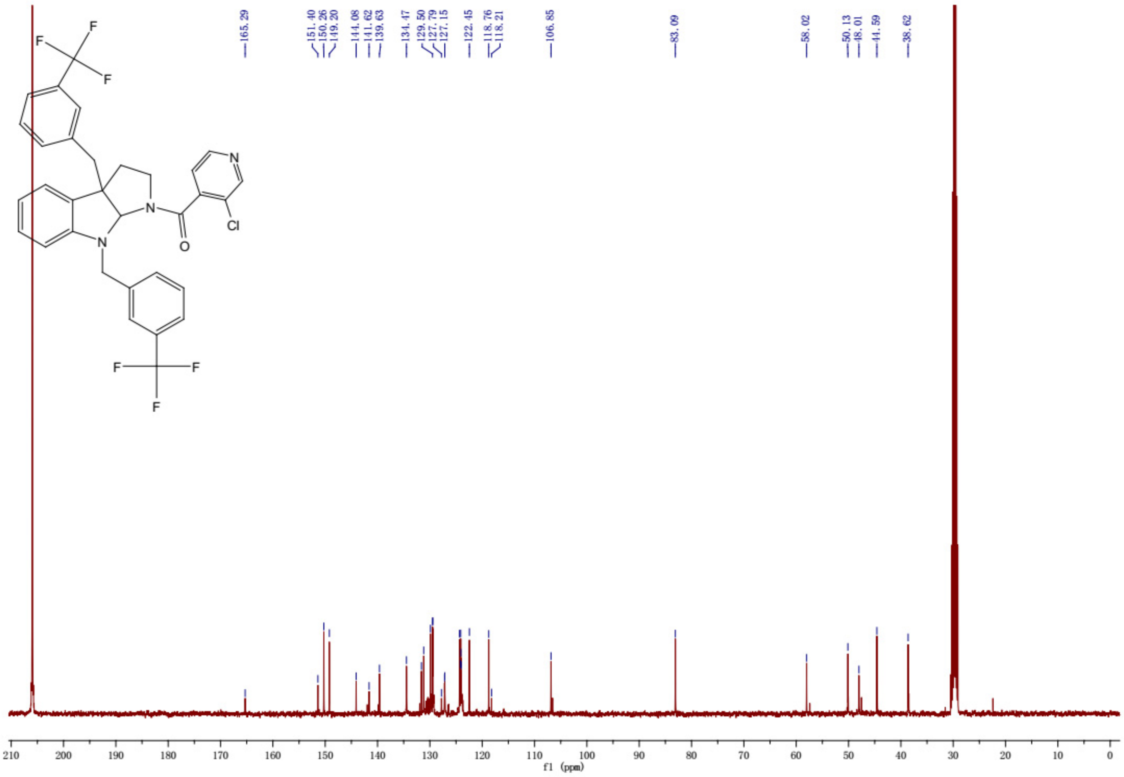

Figure S48 ^{13}C-NMR spectroscopic data of compound **b4**

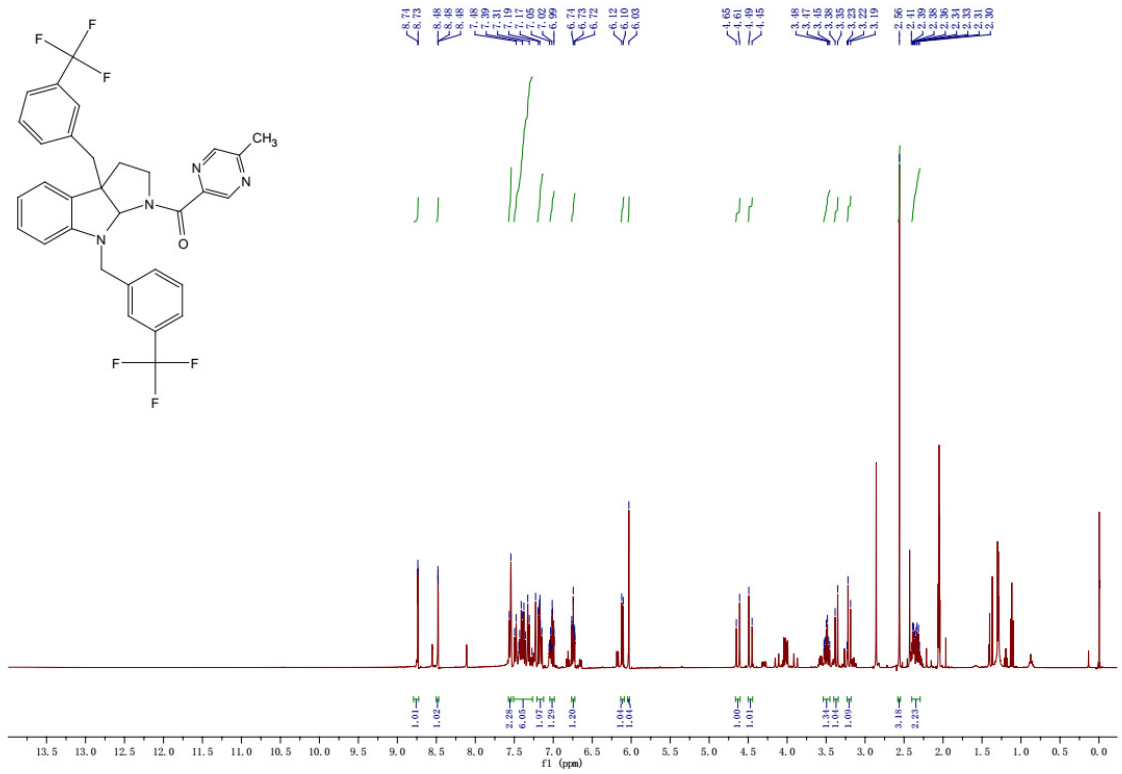

Figure S49 ^{1}H-NMR spectroscopic data of compound **b5**

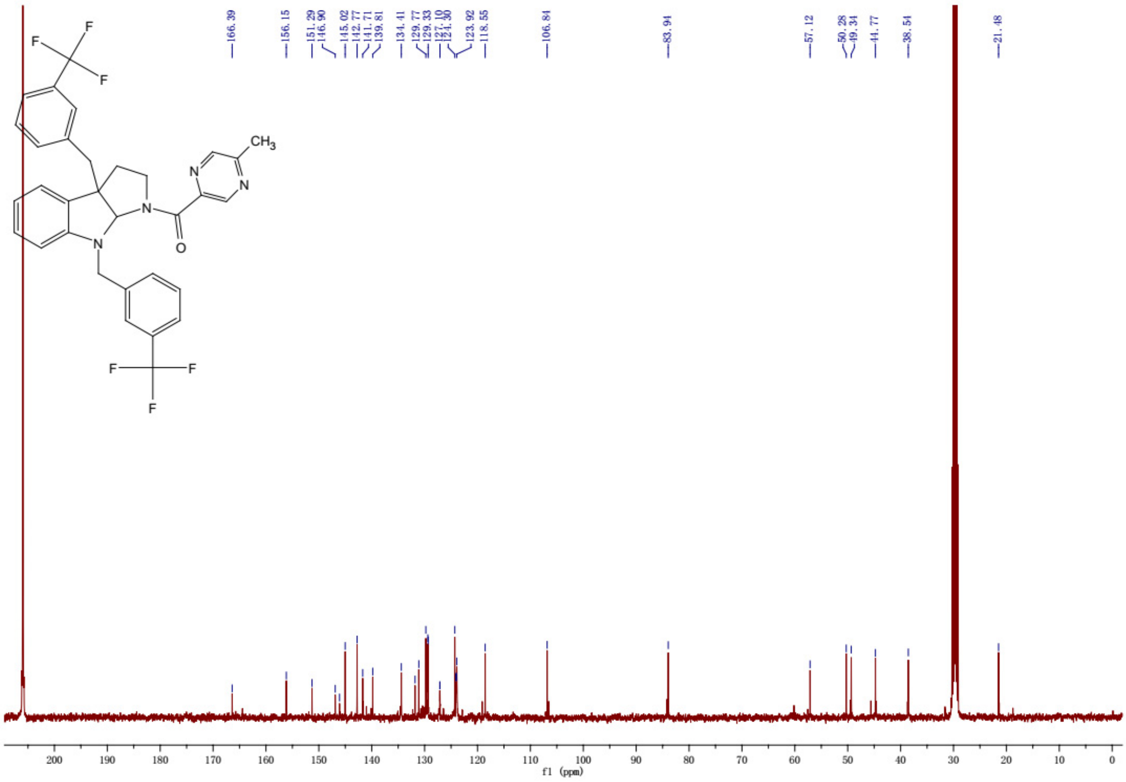

Figure S50 ^{13}C-NMR spectroscopic data of compound **b5**

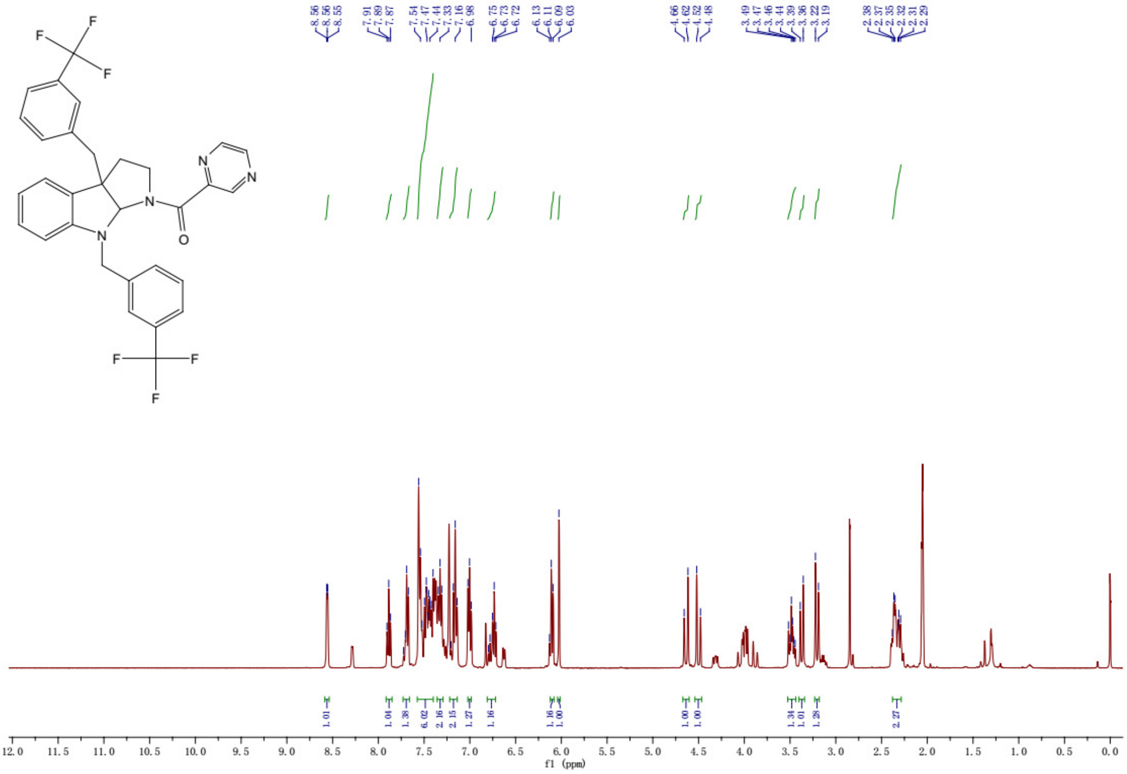

Figure S51 ^{1}H-NMR spectroscopic data of compound **b6**

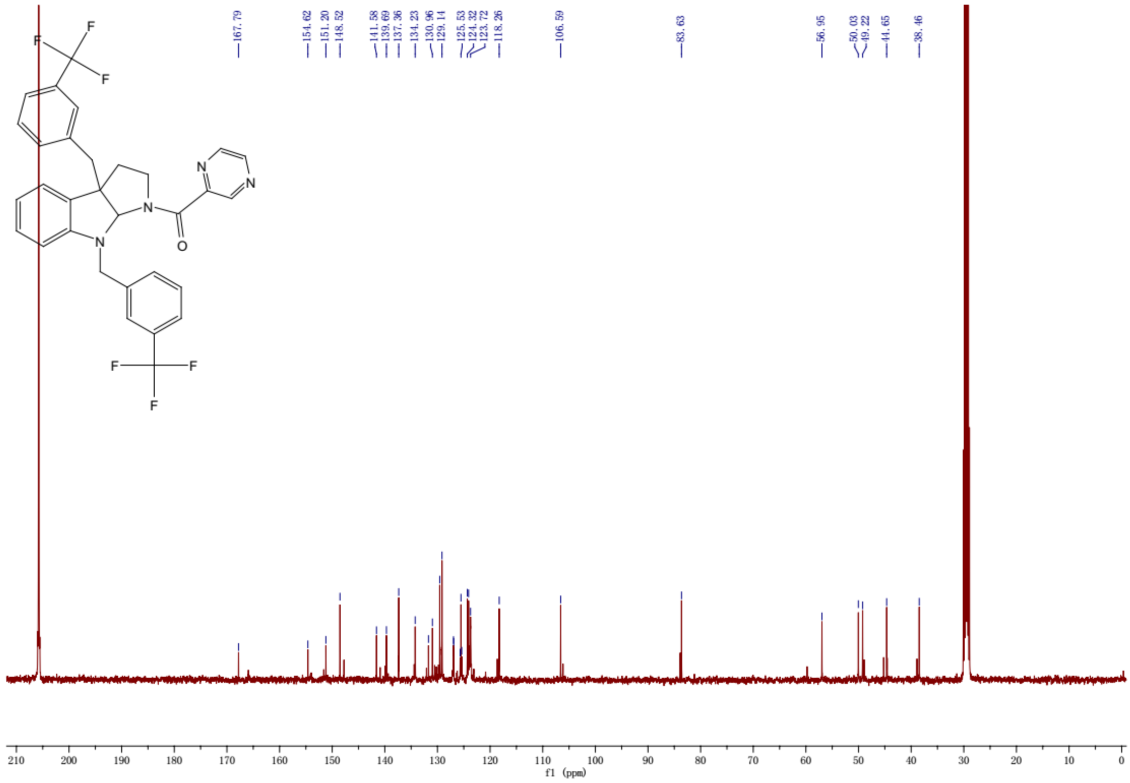

Figure S52 ^{13}C-NMR spectroscopic data of compound **b6**

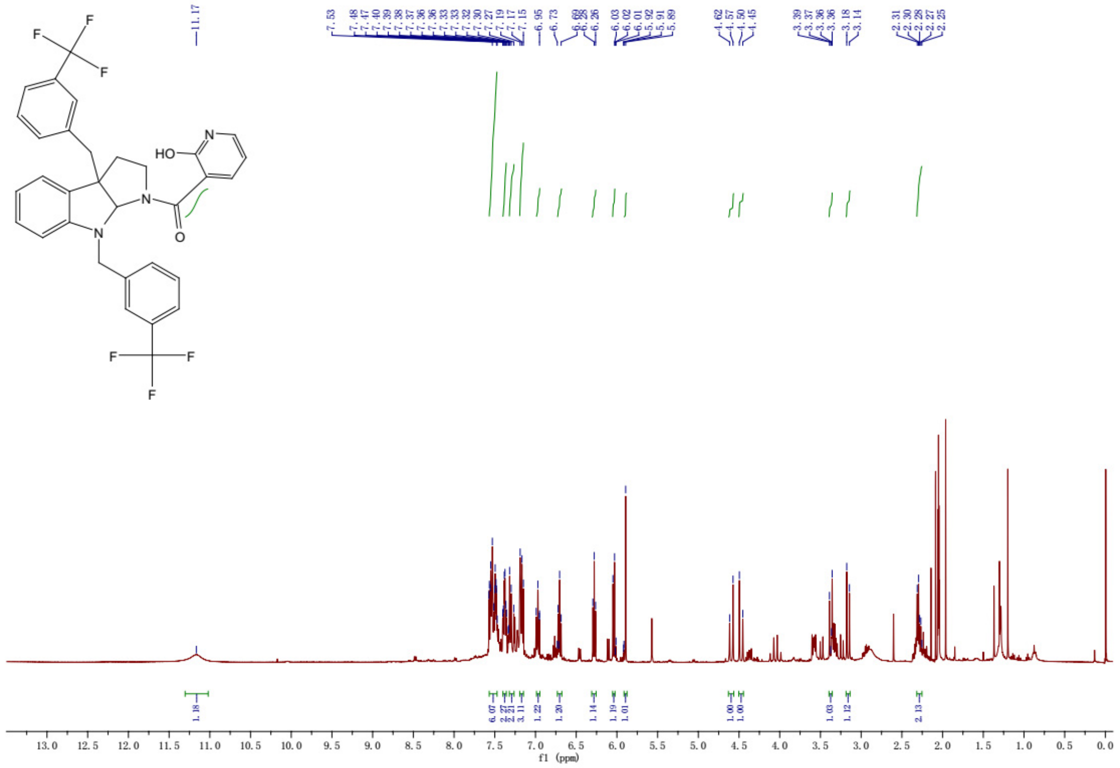

Figure S53 ^{1}H-NMR spectroscopic data of compound **b7**

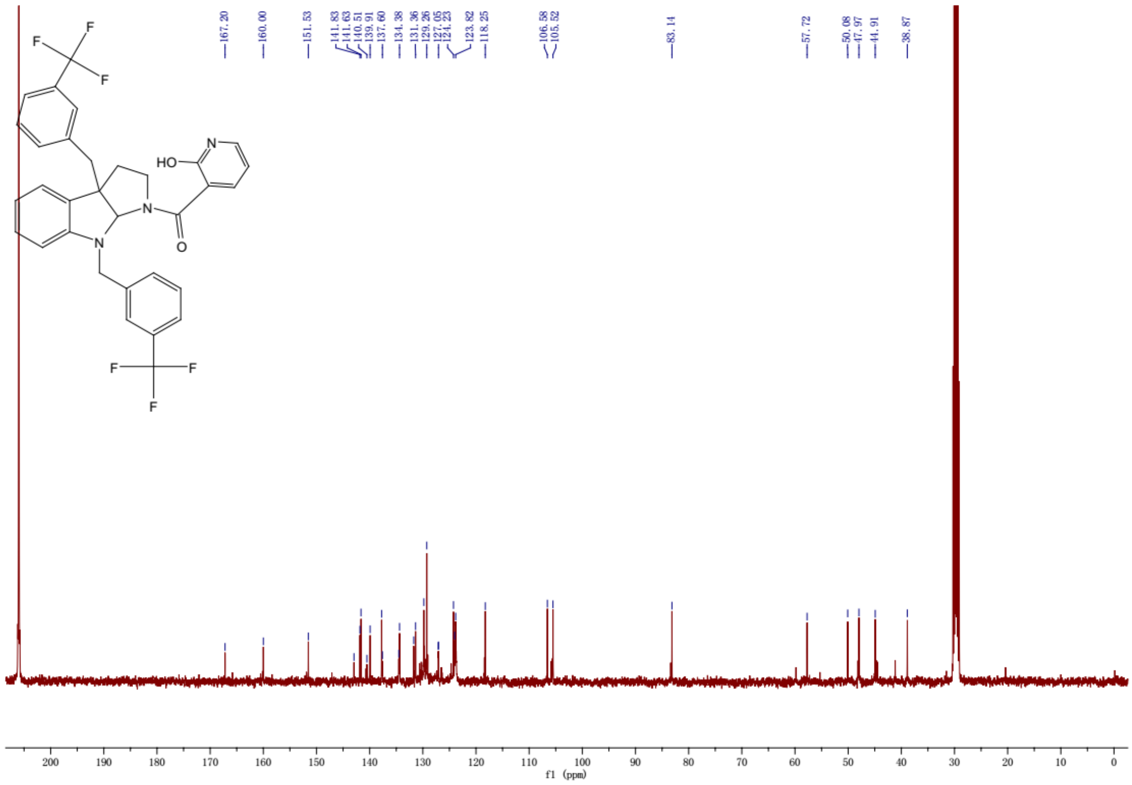

Figure S54 ^{13}C-NMR spectroscopic data of compound **b7**

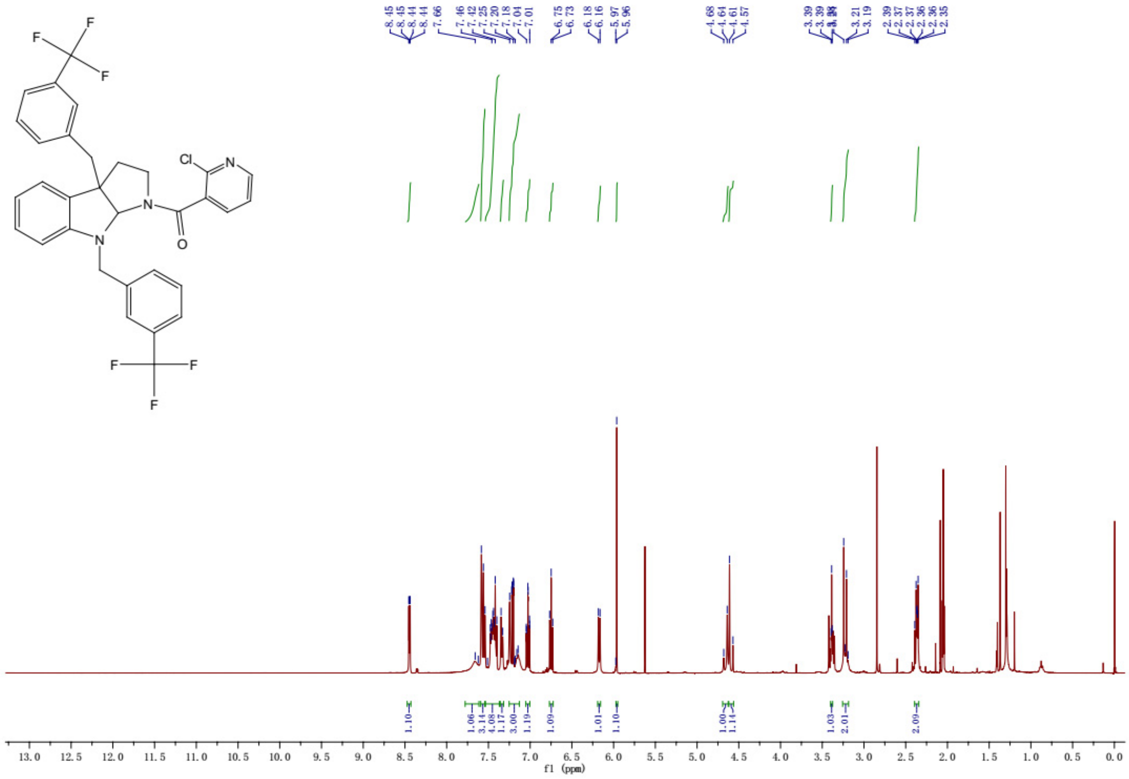

Figure S55 ^{1}H-NMR spectroscopic data of compound **b8**

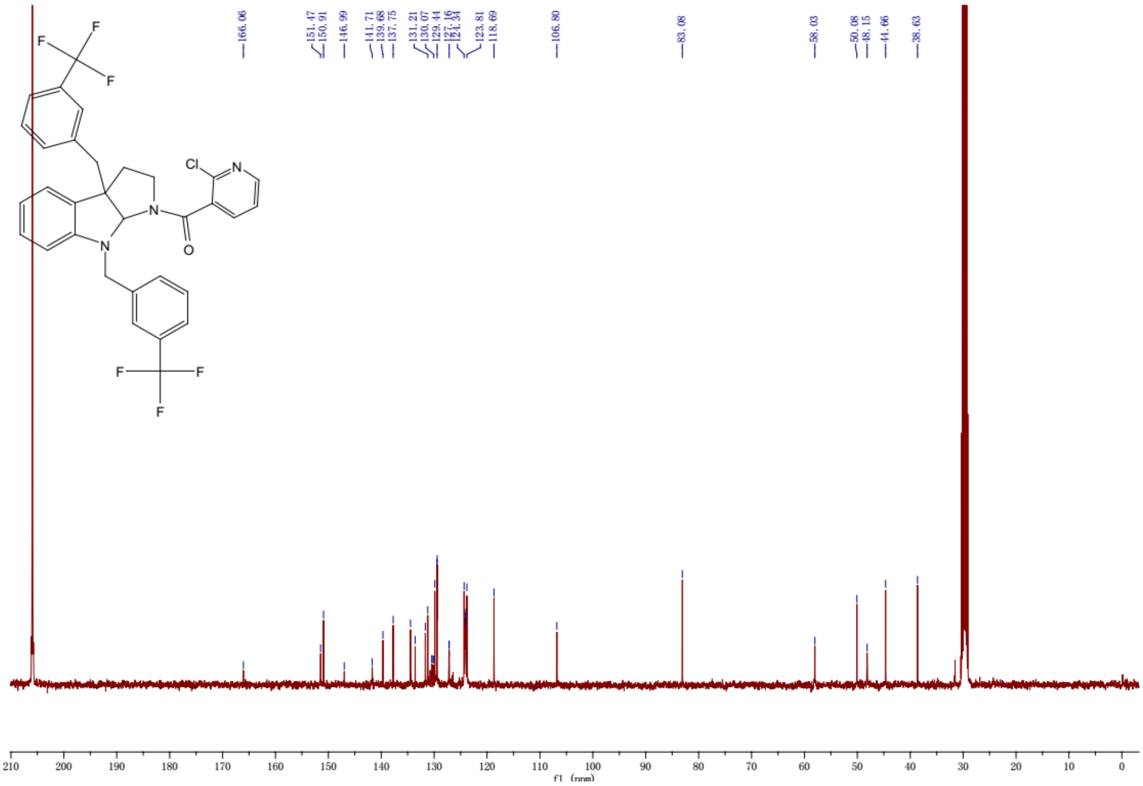

Figure S56 ^{13}C-NMR spectroscopic data of compound **b8**

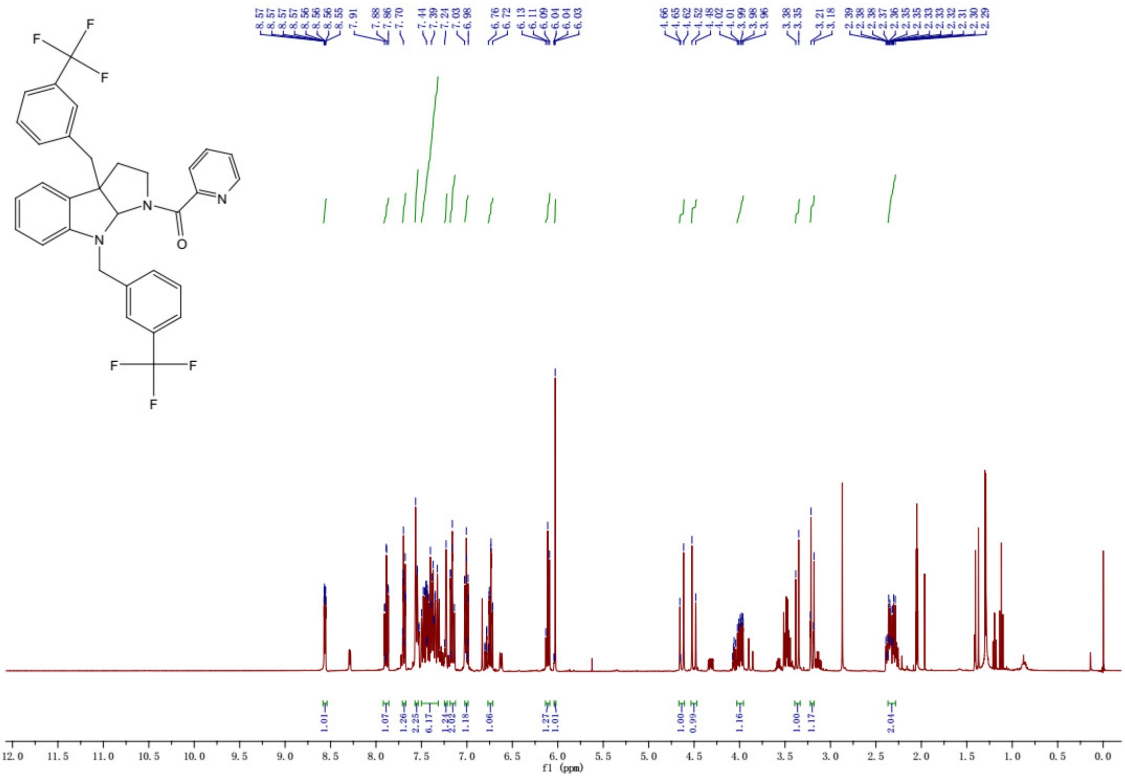

Figure S57 ^{1}H-NMR spectroscopic data of compound **b9**

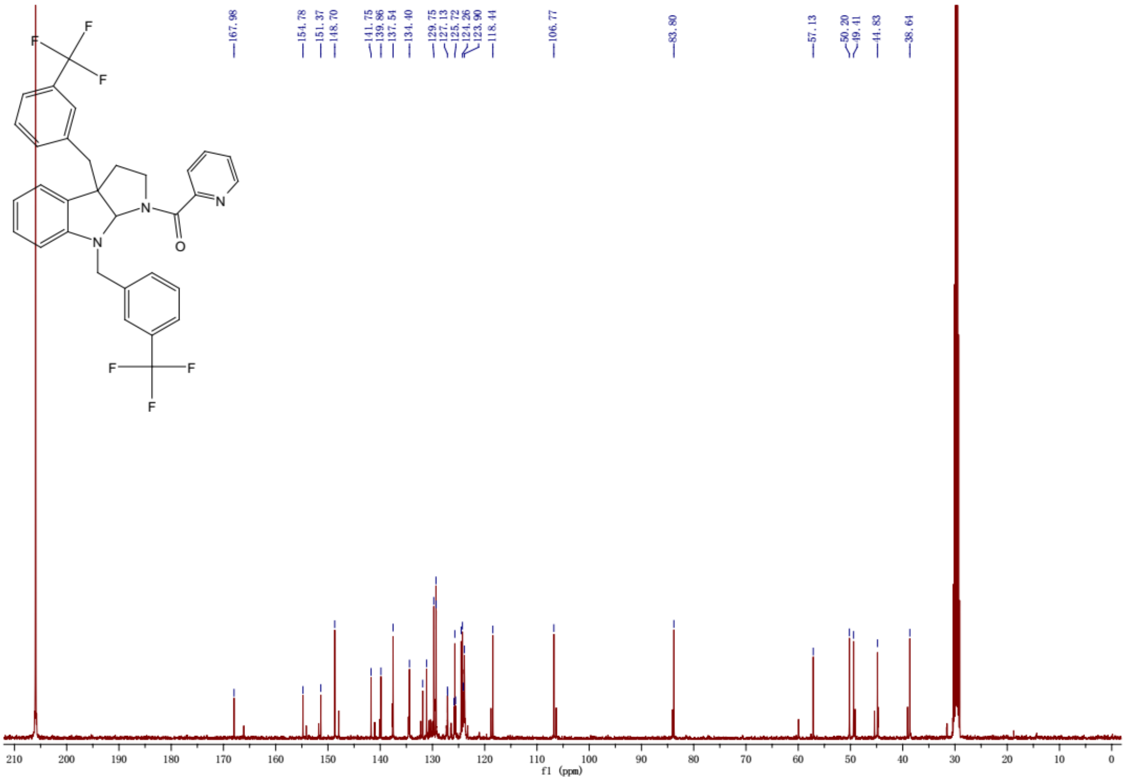

Figure S58 ^{13}C-NMR spectroscopic data of compound **b9**

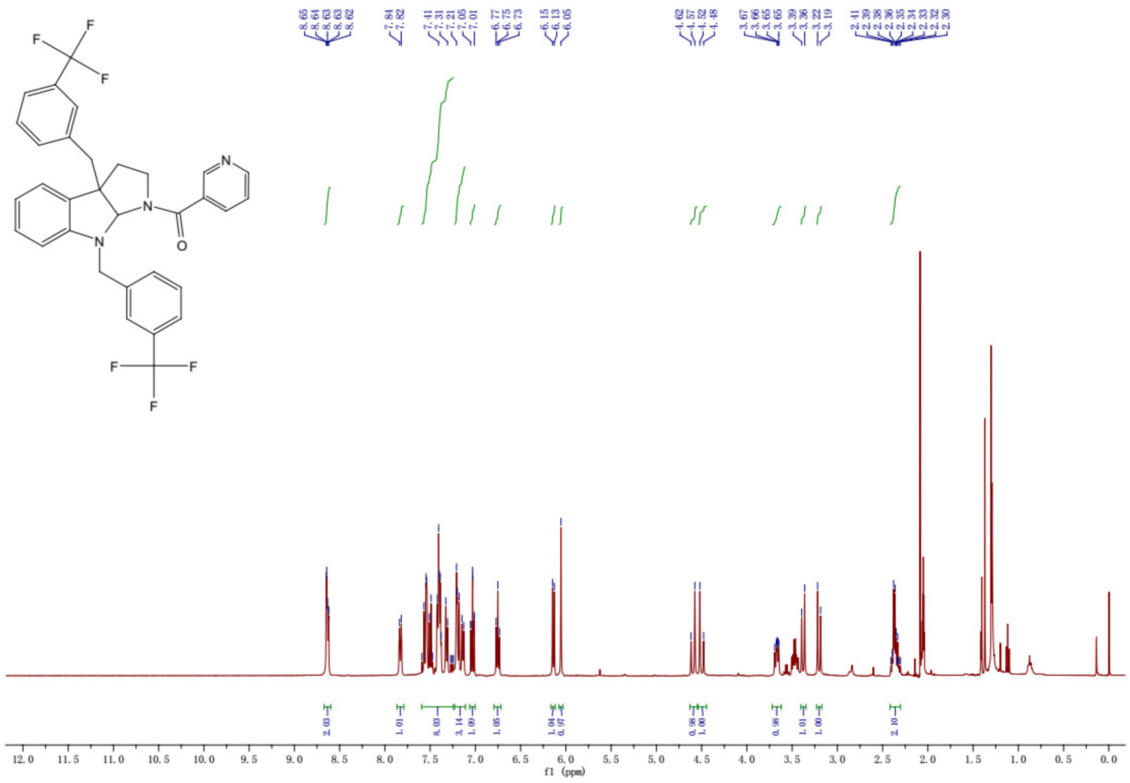

Figure S59 ^{1}H-NMR spectroscopic data of compound **b10**

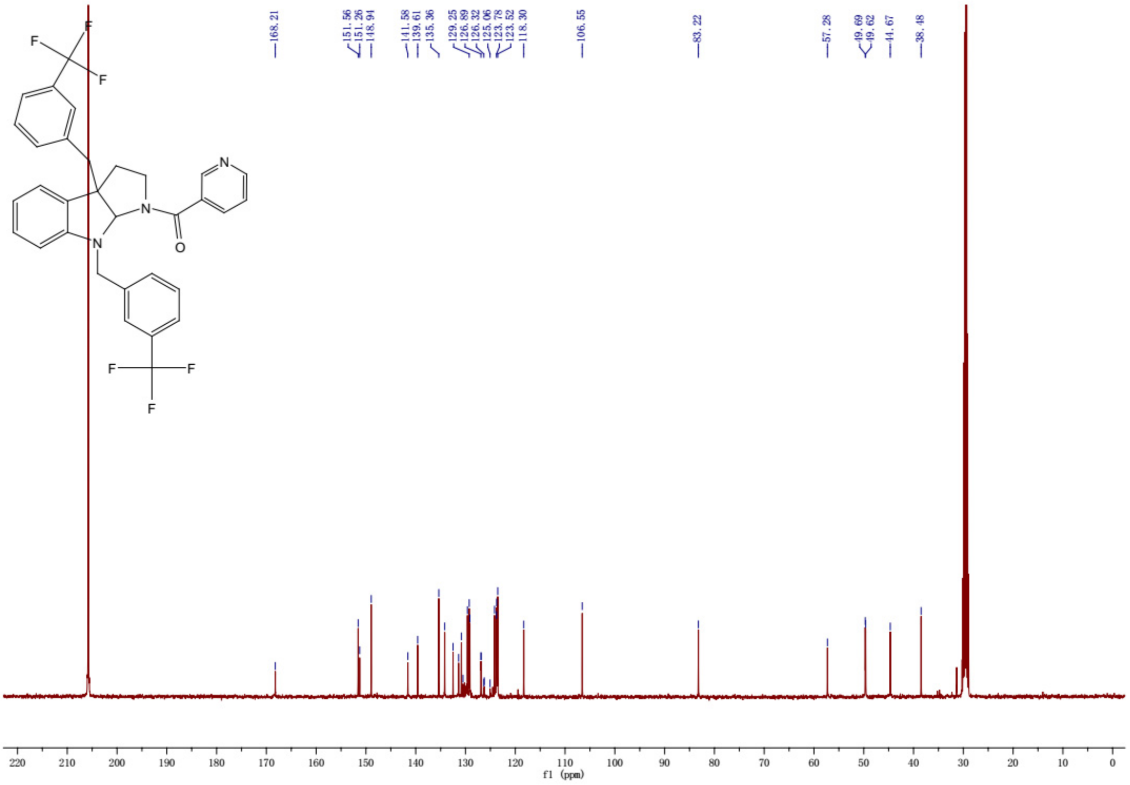

Figure S60 ^{13}C-NMR spectroscopic data of compound **b10**

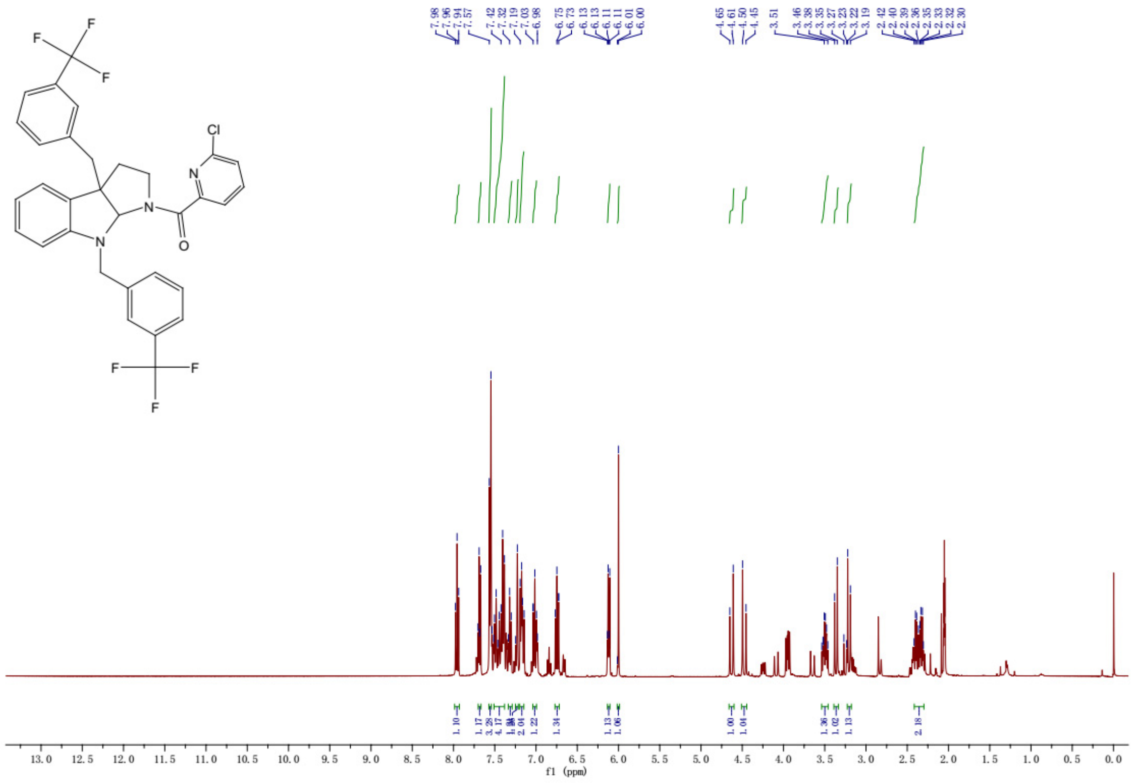

Figure S61 ^{1}H-NMR spectroscopic data of compound **b11**

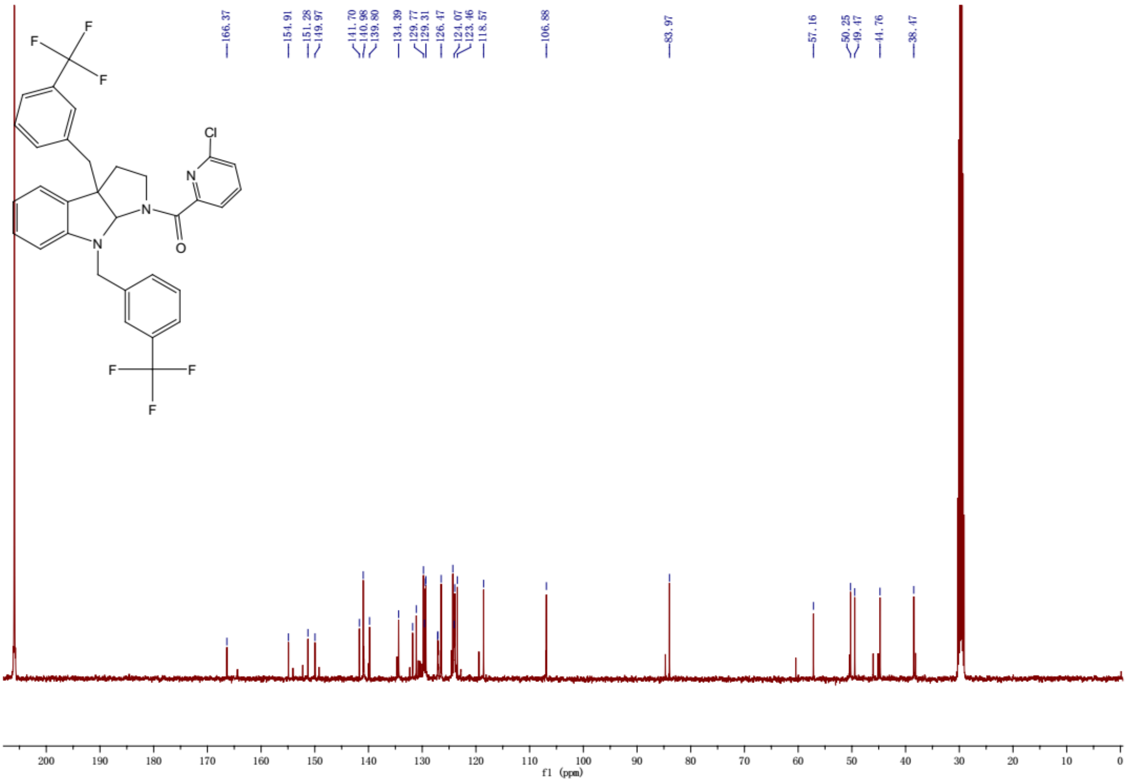

Figure S62 ^{13}C-NMR spectroscopic data of compound **b11**

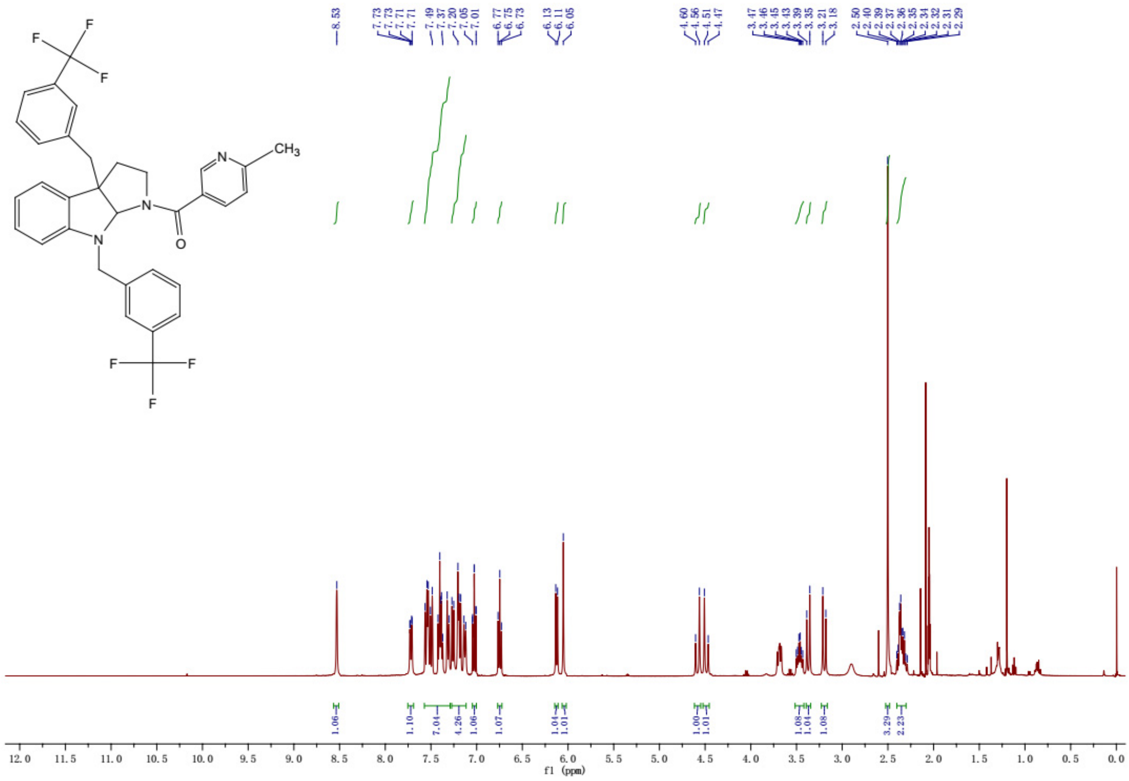

Figure S63 ^{1}H-NMR spectroscopic data of compound **b12**

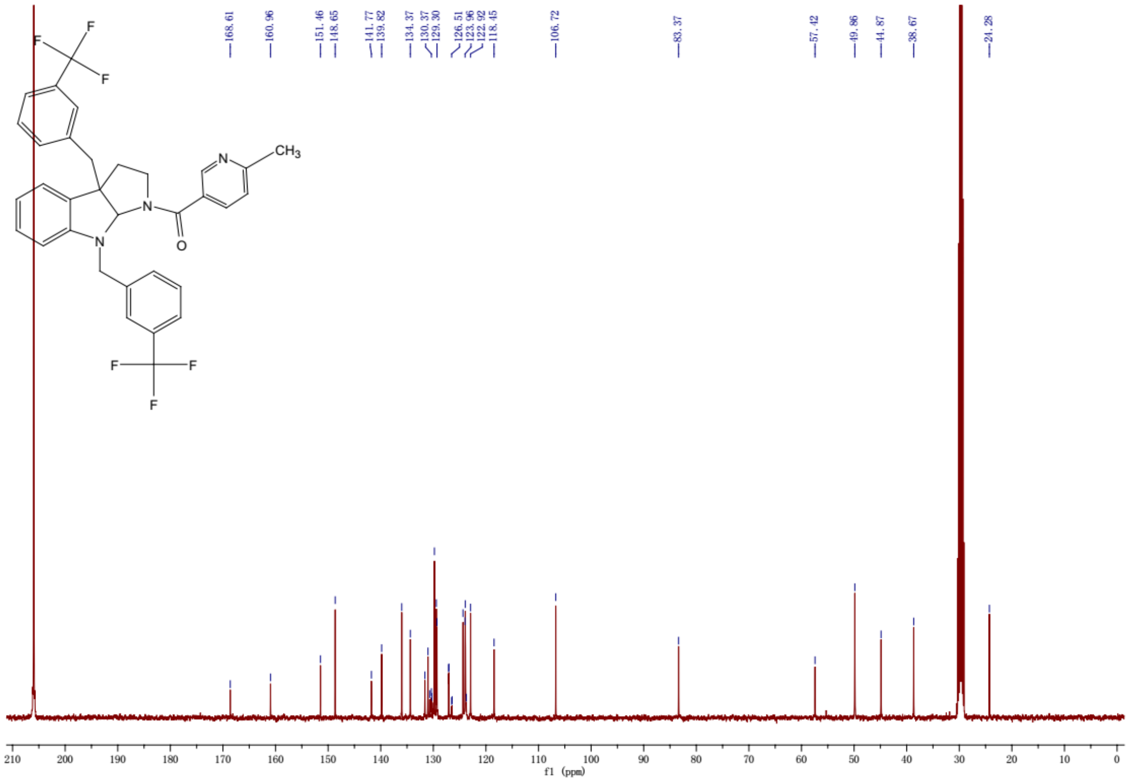

Figure S64 ^{13}C-NMR spectroscopic data of compound **b12**

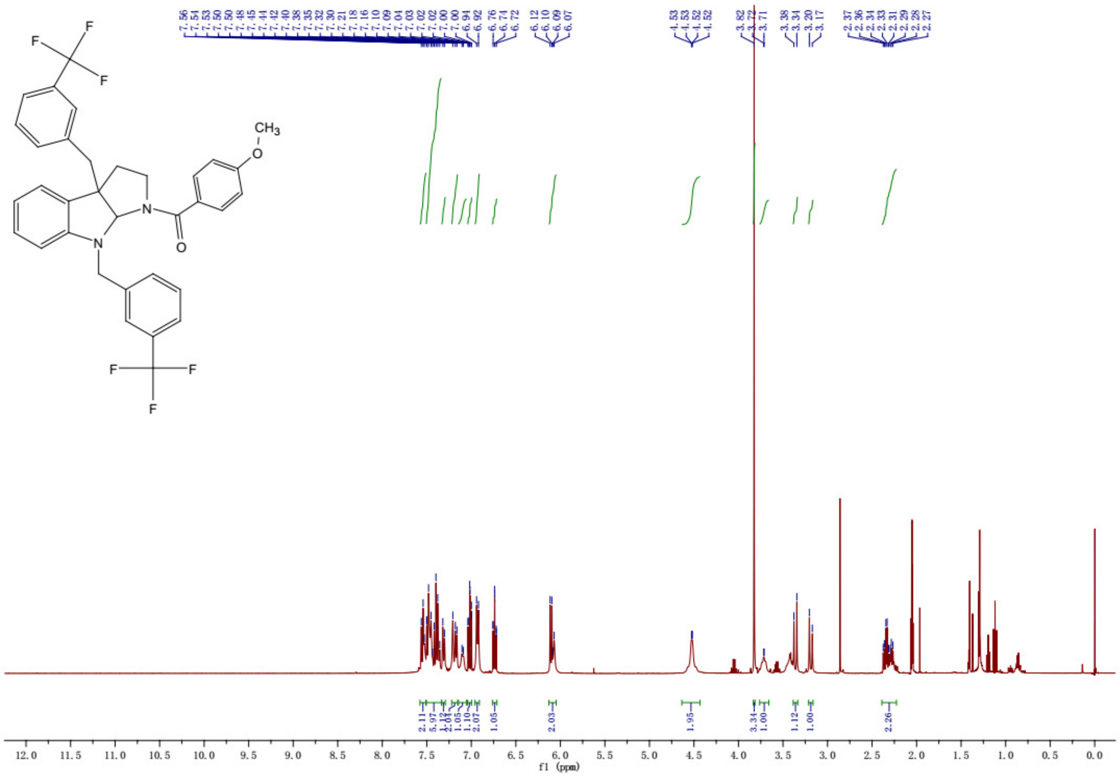

Figure S65 ^{1}H-NMR spectroscopic data of compound **b13**

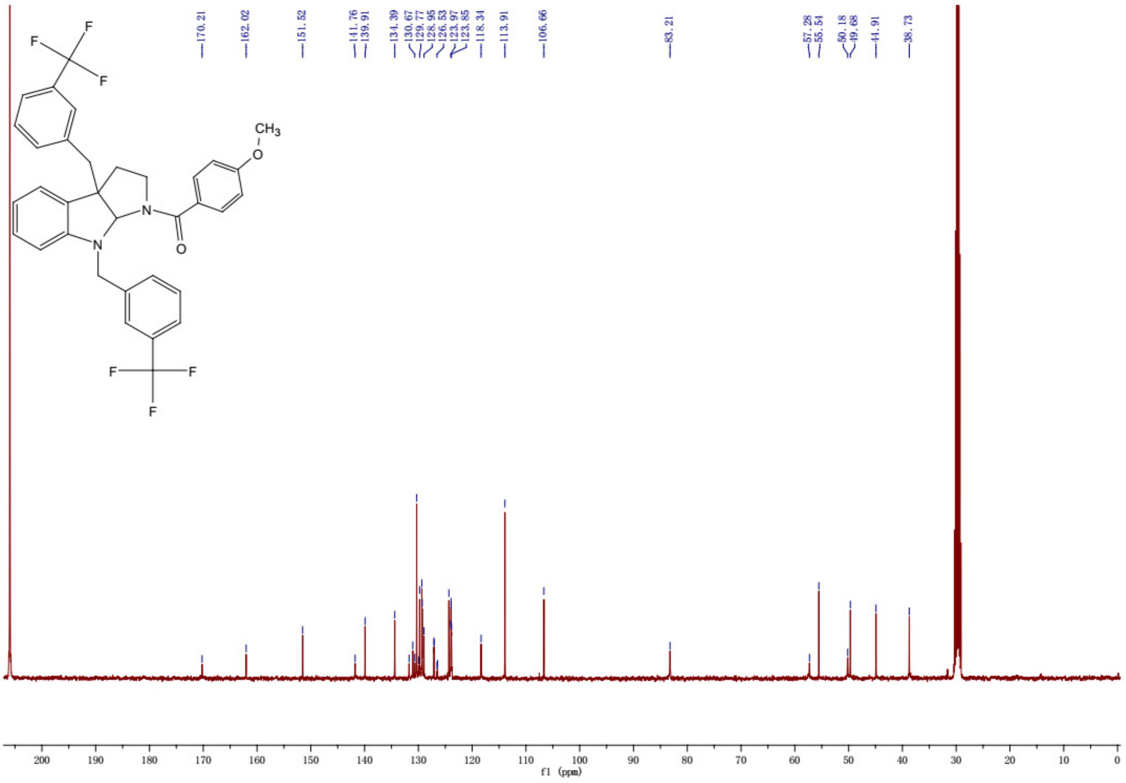

Figure S66 ^{13}C-NMR spectroscopic data of compound **b13**

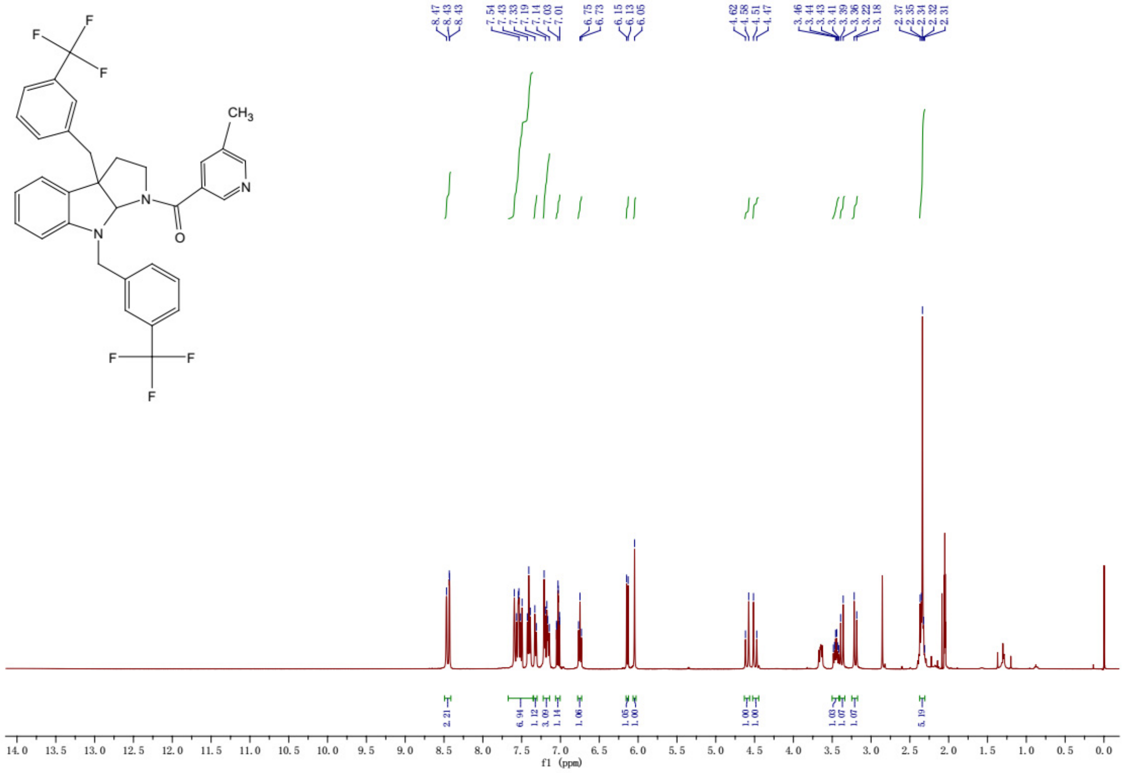

Figure S67 ^{1}H-NMR spectroscopic data of compound **b14**

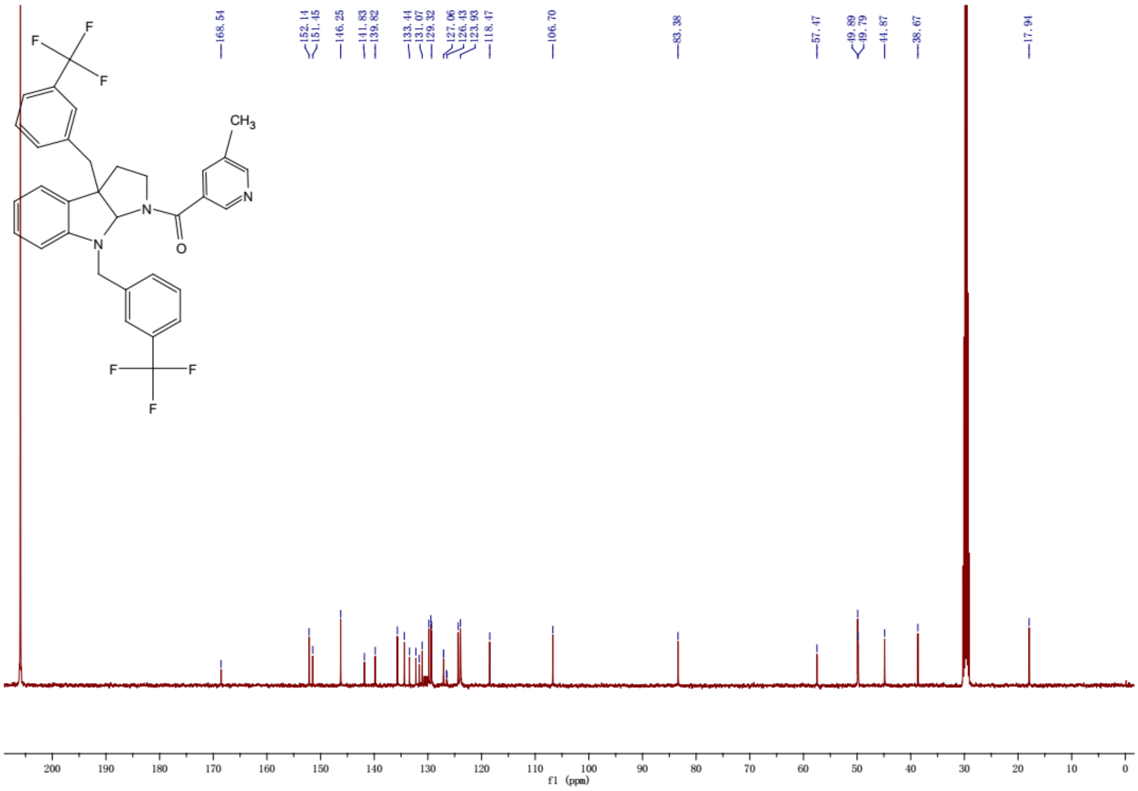

Figure S68 ^{13}C-NMR spectroscopic data of compound **b14**

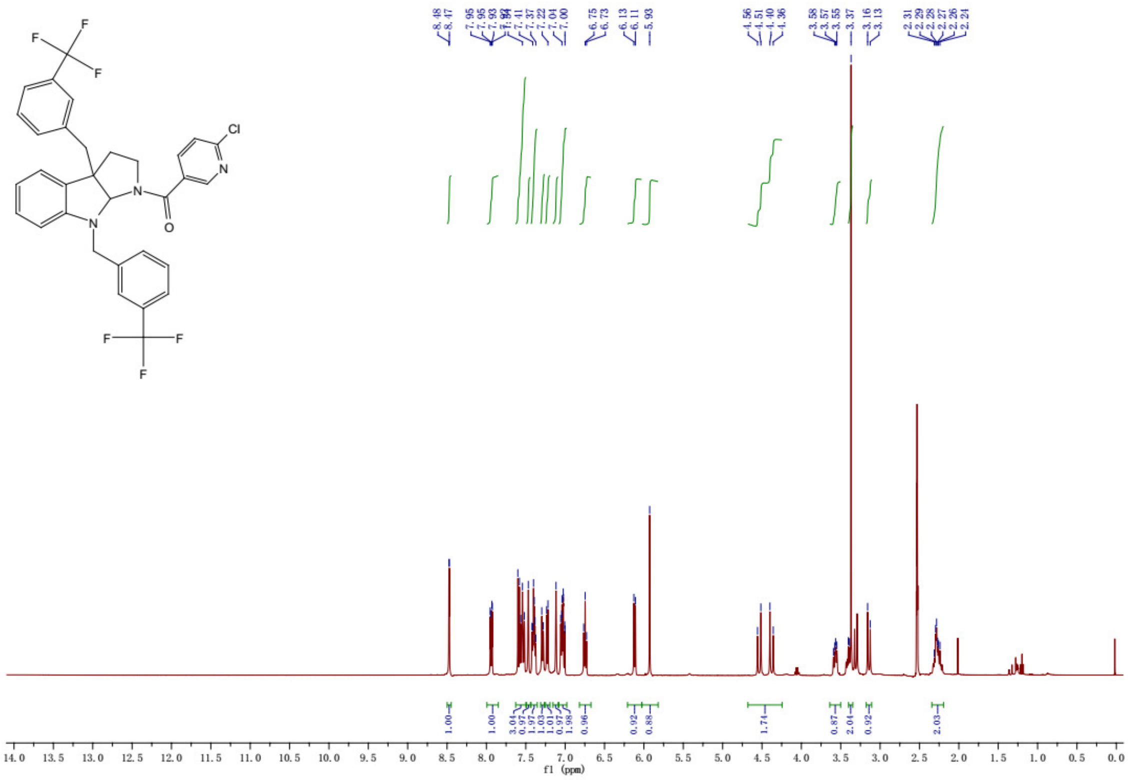

Figure S69 ^{1}H-NMR spectroscopic data of compound **b15**

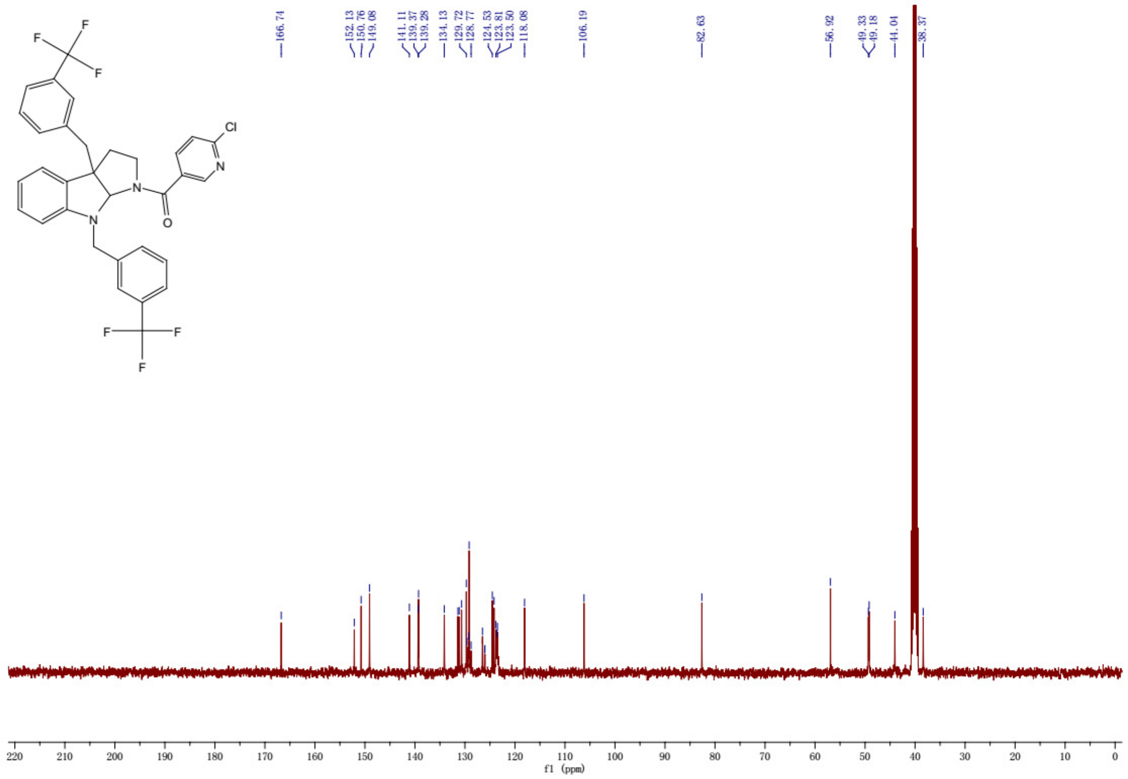

Figure S70 ^{13}C-NMR spectroscopic data of compound **b15**

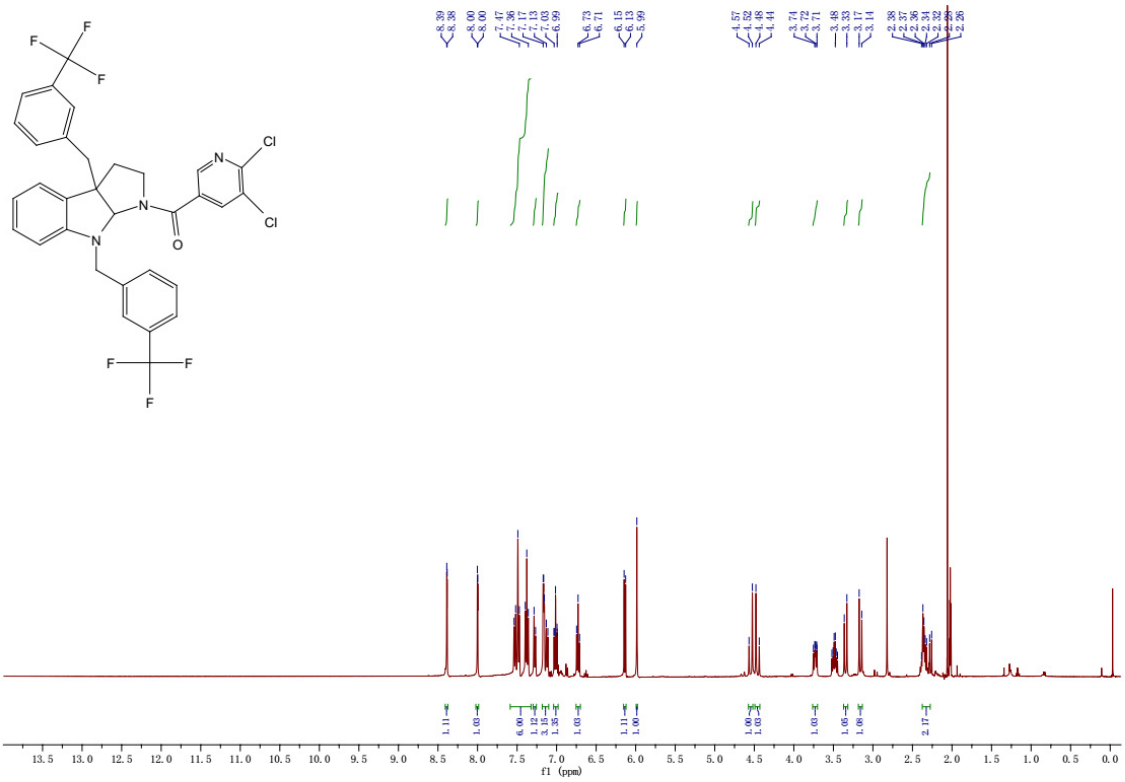

Figure S71 ^{1}H-NMR spectroscopic data of compound **b16**

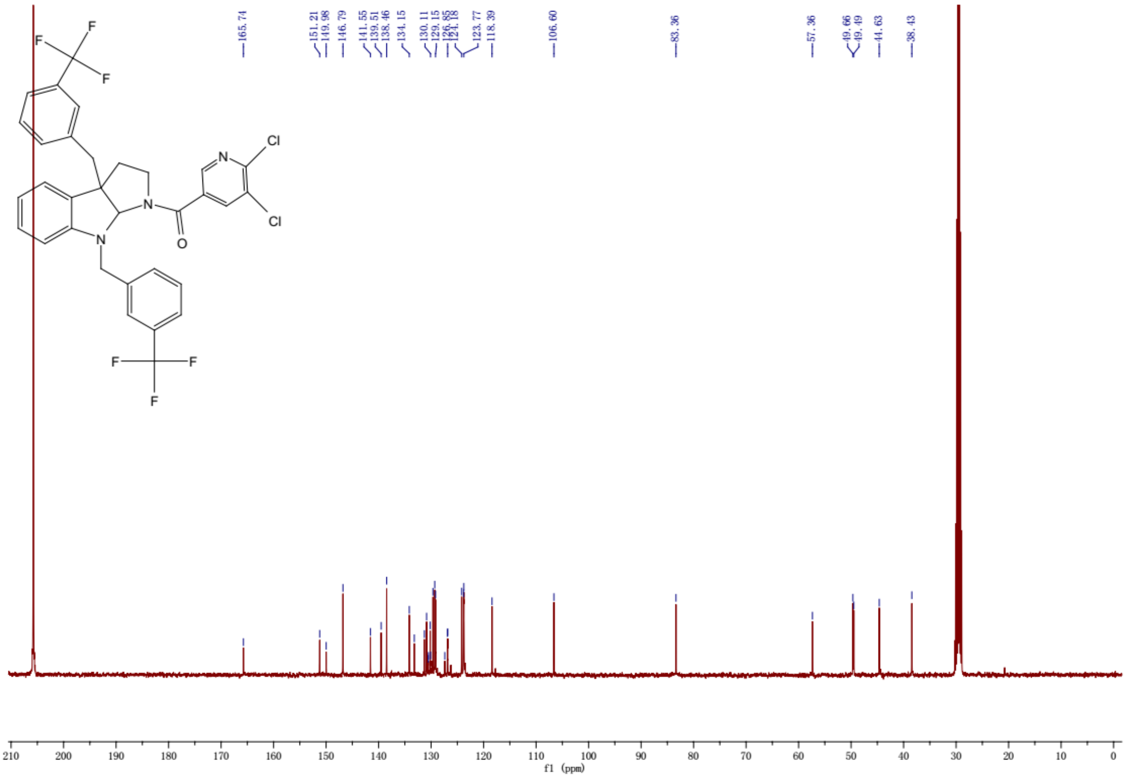

Figure S72 ^{13}C-NMR spectroscopic data of compound **b16**

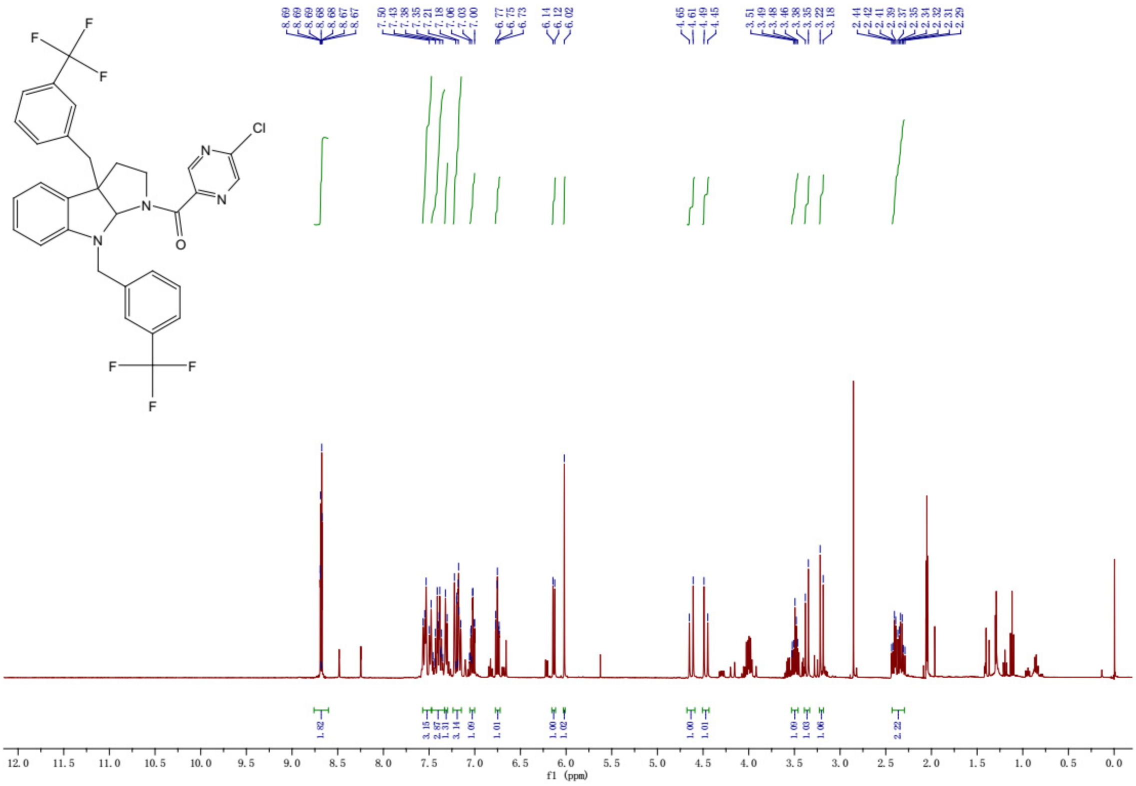

Figure S73 ^{1}H-NMR spectroscopic data of compound **b17**

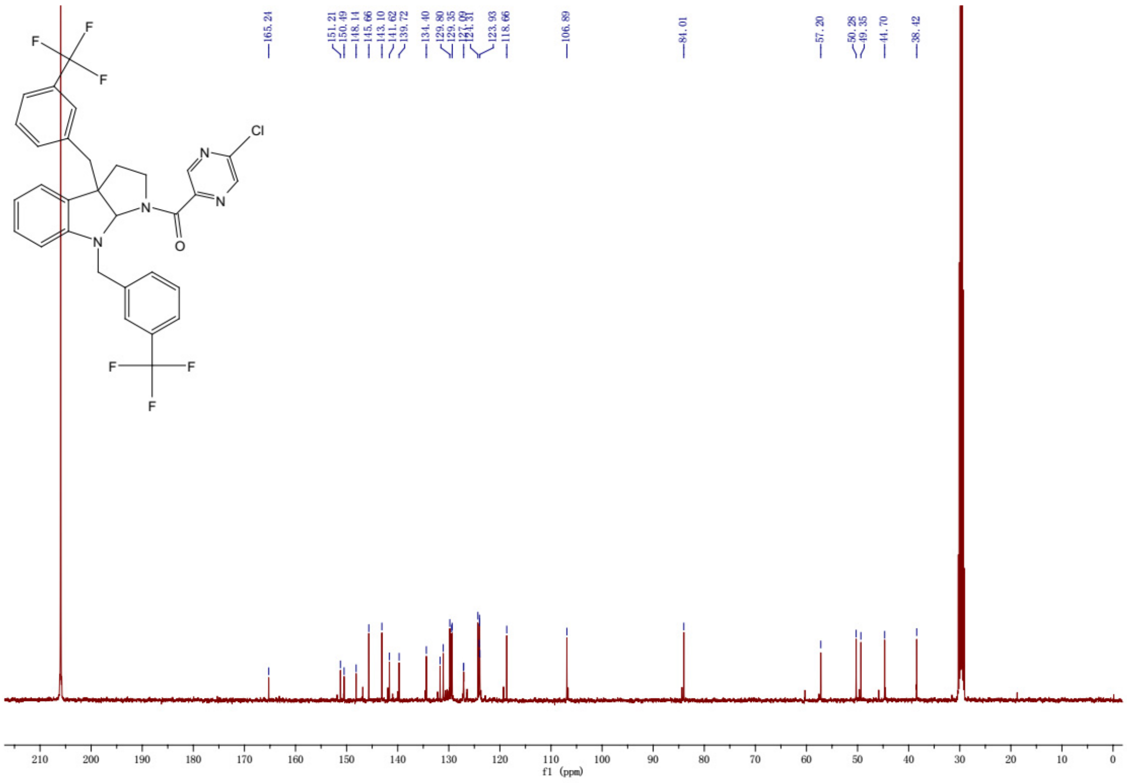

Figure S74 ^{13}C-NMR spectroscopic data of compound **b17**

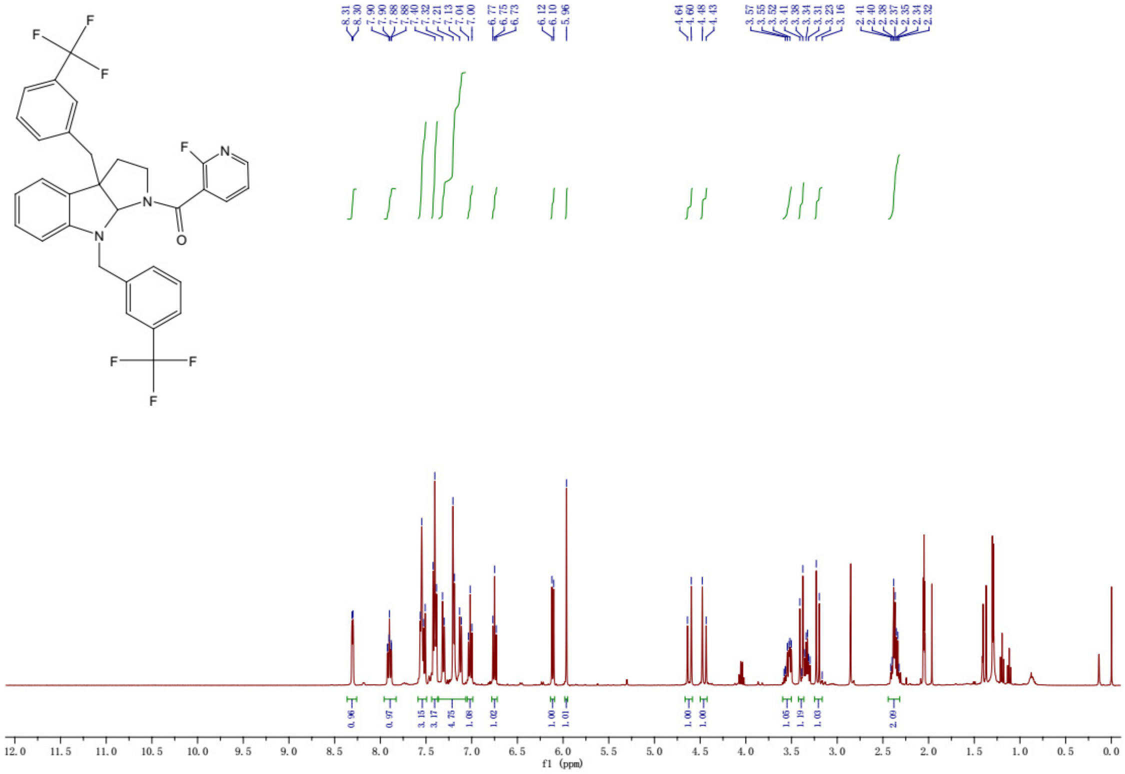

Figure S75 ^{1}H-NMR spectroscopic data of compound **b18**

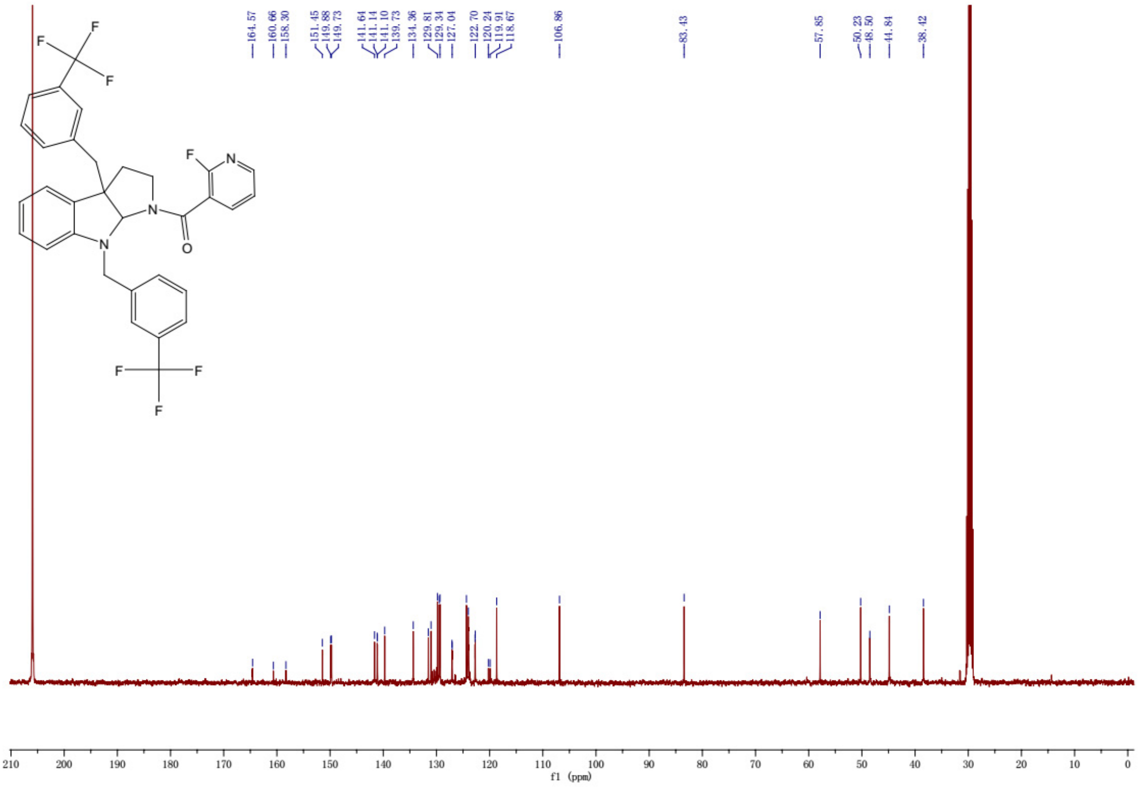

Figure S76 ^{13}C-NMR spectroscopic data of compound **b18**

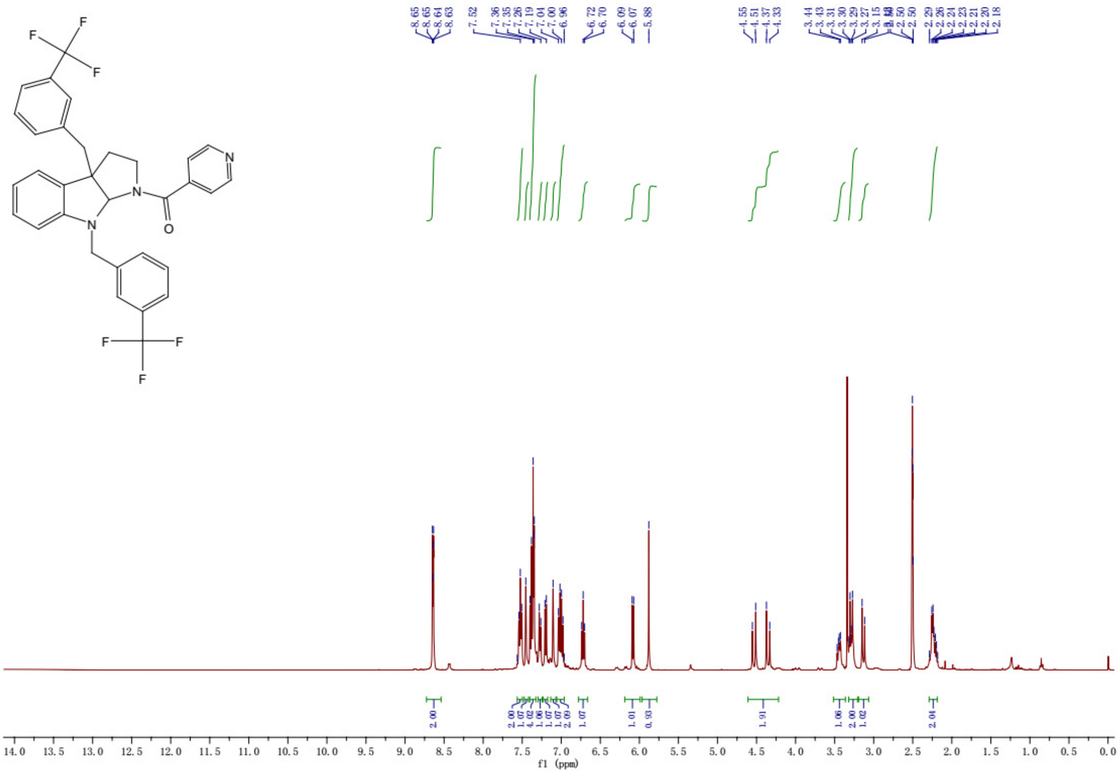

Figure S77 ^{1}H-NMR spectroscopic data of compound **b19**

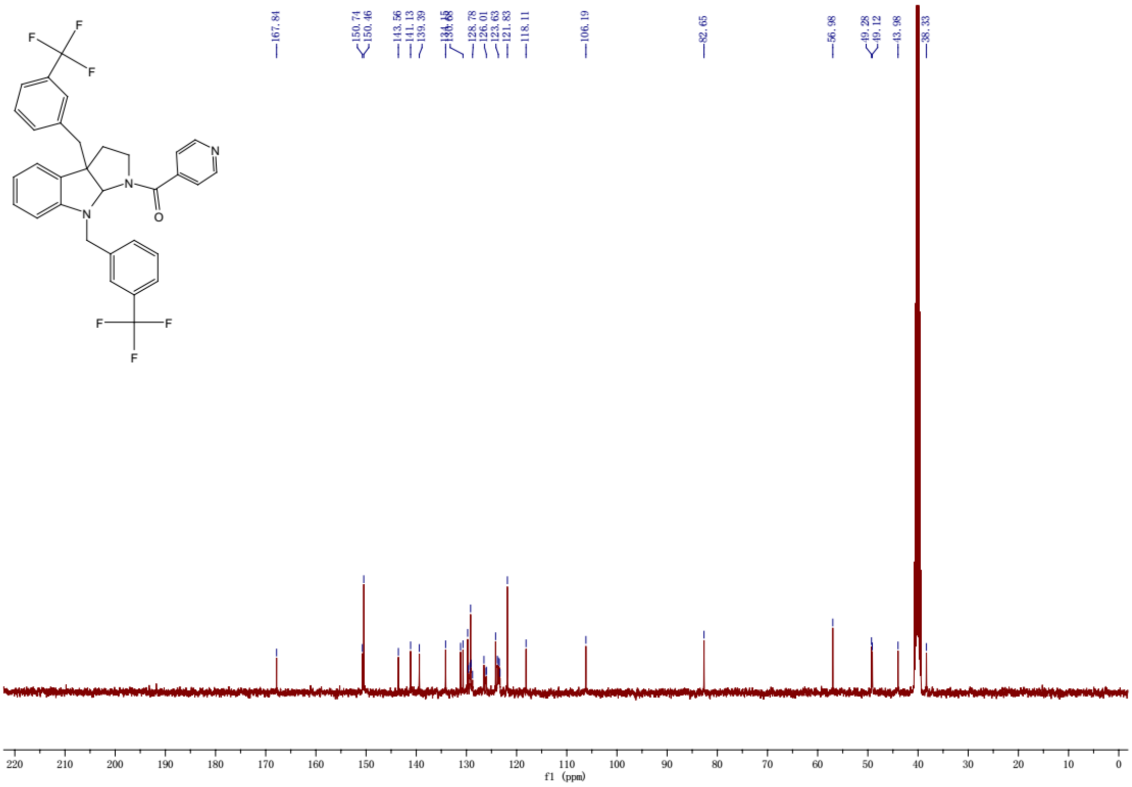

Figure S78 ^{13}C-NMR spectroscopic data of compound **b19**
